# Extracellular Vesicle‐Delivered tRF‐His‐GTG‐1 Reprograms Neutrophil Lipophagy and Triggers Inflammation in COVID‐19

**DOI:** 10.1002/advs.202508695

**Published:** 2026-01-14

**Authors:** Tsai‐Ling Liao, Po‐Yu Liu, Yi‐Ming Chen, Kuo‐Tung Tang, Hung‐Jen Liu, Der‐Yuan Chen

**Affiliations:** ^1^ Department of Medical Research Taichung Veterans General Hospital Taichung Taiwan; ^2^ Rong Hsing Research Center for Translational Medicine National Chung Hsing University Taichung Taiwan; ^3^ Ph.D. Program in Translational Medicine National Chung Hsing University Taichung Taiwan; ^4^ Division of Infection Taichung Veterans General Hospital Taichung Taiwan; ^5^ Department of Post‐Baccalaureate Medicine College of Medicine National Chung Hsing University Taichung Taiwan; ^6^ Division of Allergy Immunology and Rheumatology Department of Internal Medicine Taichung Veterans General Hospital Taichung Taiwan; ^7^ Institute of Molecular Biology National Chung Hsing University Taichung Taiwan; ^8^ The iEGG and Animal Biotechnology Center National Chung Hsing University Taichung Taiwan; ^9^ Translational Medicine Laboratory Rheumatology and Immunology Center China Medical University Hospital Taichung Taiwan; ^10^ College of Medicine China Medical University Taichung Taiwan

**Keywords:** COVID‐19, extracellular vesicles, lipophagy, neutrophil extracellular traps, transfer RNA‐derived small RNAs

## Abstract

Immunometabolism and neutrophil extracellular traps (NETs) play pivotal roles in the pathogenesis of coronavirus disease 2019 (COVID‐19) and its postacute sequelae. However, the upstream regulators that reprogram neutrophil lipid metabolism and trigger excessive NET formation remain largely undefined. This study identifies a transfer RNA‐derived fragment, tRF‐His‐GTG‐1, enriched in platelet‐derived extracellular vesicles, as a key driver of neutrophil lipophagy dysfunction and inflammation in COVID‐19. The use on neutrophils from 60 patients and 20 healthy controls, a severe acute respiratory syndrome coronavirus 2 (SARS‐CoV‐2)–infected hamster model, and multiple in vitro assays shows that severe COVID‐19 and long COVID are characterized by increased lipid droplet (LD) accumulation and NET release. Mechanistically, tRF‐His‐GTG‐1 activates Toll‐like receptor 8 (TLR8)–mammalian target of rapamycin (mTOR) signaling and suppresses RAB7A expression, changes that impair lipophagic flux. This dual pathway impairs lipophagy and promotes NET formation and proinflammatory cytokine secretion. Importantly, ex vivo treatment with a tRF‐His‐GTG‐1 inhibitor restores lipophagy, reduces LD and NET levels, and suppresses interleukin 1beta (IL‐1β)/IL‐8 production in patient‐derived neutrophils. These findings reveal a novel EV‐mediated immunometabolic axis linking platelets to neutrophil dysfunction, and position tRF‐His‐GTG‐1 as a promising RNA‐based therapeutic target for COVID‐19‐associated hyperinflammation.

## Graphical abstract

This study identifies platelet‐derived extracellular vesicles as key immunometabolic regulators in COVID‐19. The delivery of tRF‐His‐GTG‐1 to neutrophils activates TLR8mTOR signaling, disrupts lipophagy, and amplifies NET‐mediated inflammation. Importantly, targeting this axis restores neutrophil homeostasis, offering a potential therapeutic strategy for acute and long COVID.

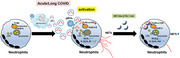

## 1 Introduction

Neutrophils are the most abundant innate immune cells and serve as the first line of defense against pathogens. One of their key antimicrobial strategies is the release of neutrophil extracellular traps (NETs), which capture and eliminate invading microbes [1]. However, excessive NET formation contributes to coagulopathy, immunothrombosis, and tissue‐damaging inflammation, particularly in severe cases of coronavirus disease 2019 (COVID‐19) [2, 3, 4]. Recent studies have implicated NETs in sustaining chronic inflammation in patients with long COVID [5], but the mechanisms driving their overproduction and persistence remain poorly understood.

Lipid homeostasis is essential for neutrophil function [6], but its contribution to NET formation has been largely overlooked. Lipid droplets (LDs) are highly dynamic organelles involved in lipid metabolism [7] and have emerged as key regulators of inflammation and immunity during viral infections [8, 9]. Increasing evidence suggests that LD plasticity is a hallmark of severe acute respiratory syndrome coronavirus 2 (SARS‐CoV‐2) infection [10, 11, 12]. However, the precise contribution of LDs to neutrophil function and NET induction remains poorly defined.

Extracellular vesicles (EVs) mediate intercellular communication by delivering bioactive molecules and modulating immune responses and metabolic processes [13]. Among them, platelets are a major source of circulating EVs [14], which have been implicated in the pathogenesis of thrombosis [15]. Platelet‐derived extracellular vesicles (pEVs) are elevated in patients with severe COVID‐19 and are associated with enhanced NET formation [16, 17, 18]. Meanwhile, small noncoding RNAs, including transfer RNA‐derived small RNAs (tsRNAs), have emerged as important regulators of immunometabolic signaling [19, 20]. Recent transcriptomic analyses have identified several tsRNAs enriched in EVs derived from patients with COVID‐19 [21, 22], but their functional role in immune‐metabolic dysregulation remains unexplored.

In this study, we investigated the role of SARS‐CoV‐2‐triggered pEVs in modulating LD accumulation in neutrophils and their impact on NET formation. Using clinical specimens, a COVID‐19 hamster model [23], and in vitro cell‐based assays, we aimed to elucidate the molecular mechanisms by which pEVs modulate lipid metabolism and drive excessive NET formation and persistence in the pathogenesis of both acute COVID‐19 and long COVID.

## 2 Results

### 2.1 Clinical Characteristics of the Study Participants

Table 1 presents the demographic and clinical information for the study cohort. The patients with moderate to severe COVID‐19 were older and had markedly higher C‐reactive protein (CRP) levels and neutrophil‐to‐lymphocyte ratios than those with mild COVID‐19. Comorbidities, particularly hypertension and diabetes, were more frequent in patients with severe COVID‐19, all of whom required intensive care unit (ICU) admission. In contrast, patients who had recovered from COVID‐19 and healthy controls exhibited near‐normal inflammatory profiles, whereas individuals with long COVID showed mildly elevated CRP levels without requiring ICU care.

**TABLE 1:** Demographic and clinical characteristics of the study participants.

Variable	Severe COVID‐19	Moderate COVID‐19	Mild COVID‐19	Long COVID	Recovered COVID‐19	Healthy control
	(*n* = 6)	(*n* = 10)	(*n* = 24)	(*n* = 10)	(*n* = 10)	(*n* = 20)
Age (years)	63.8±9.1	58.7±12.6	53.6±10.2	48.2±7.5	43.8±9.1	40.5±6.8
Gender (female, %)	3 (50.0%)	6 (60.0%)	12 (50.0%)	5 (50.0%)	5 (50.0%)	11 (55.0%)
Comorbidities	6 (100.0%)	7 (70.0%)	16 (66.7%)	8 (80.0%)	3 (30.0%)	7 (35.0%)
Hypertension	4 (66.7%)	4 (40.0%)	4 (16.7%)	3 (30.0%)	1 (10.0%)	2 (10.0%)
Diabetes mellitus	3 (50.0%)	4 (40.0%)	3 (12.5%)	2 (20.0%)	1 (10.0%)	1 (5.0%)
CRP (mg/L)	140.2±35.6	81.2±28.7	25.6±12.3	18.9±25.1	3.6±5.1	NA
Neutrophil‐to‐lymphocyte	11.3±5.2	7.1±3.8	3.2±2.1	2.9±3.0	1.2±1.8	NA
Hospitalization days	17.6±3.8	12.3±2.5	0	0	0	0
ICU admission	6 (100.0%)	4 (40.0%)	0 (0.0%)	0 (0.0%)	0 (0.0%)	0 (0.0%)

*Note*: Laboratory data were collected at the time of blood sampling. Values are presented as mean ± SD or n (%). CRP, C‐reactive protein; ICU, intensive care unit; NA, not available.

### 2.2 LD Accumulation and NET Formation are Enhanced in COVID‐19 Neutrophils

**FIGURE 1:**
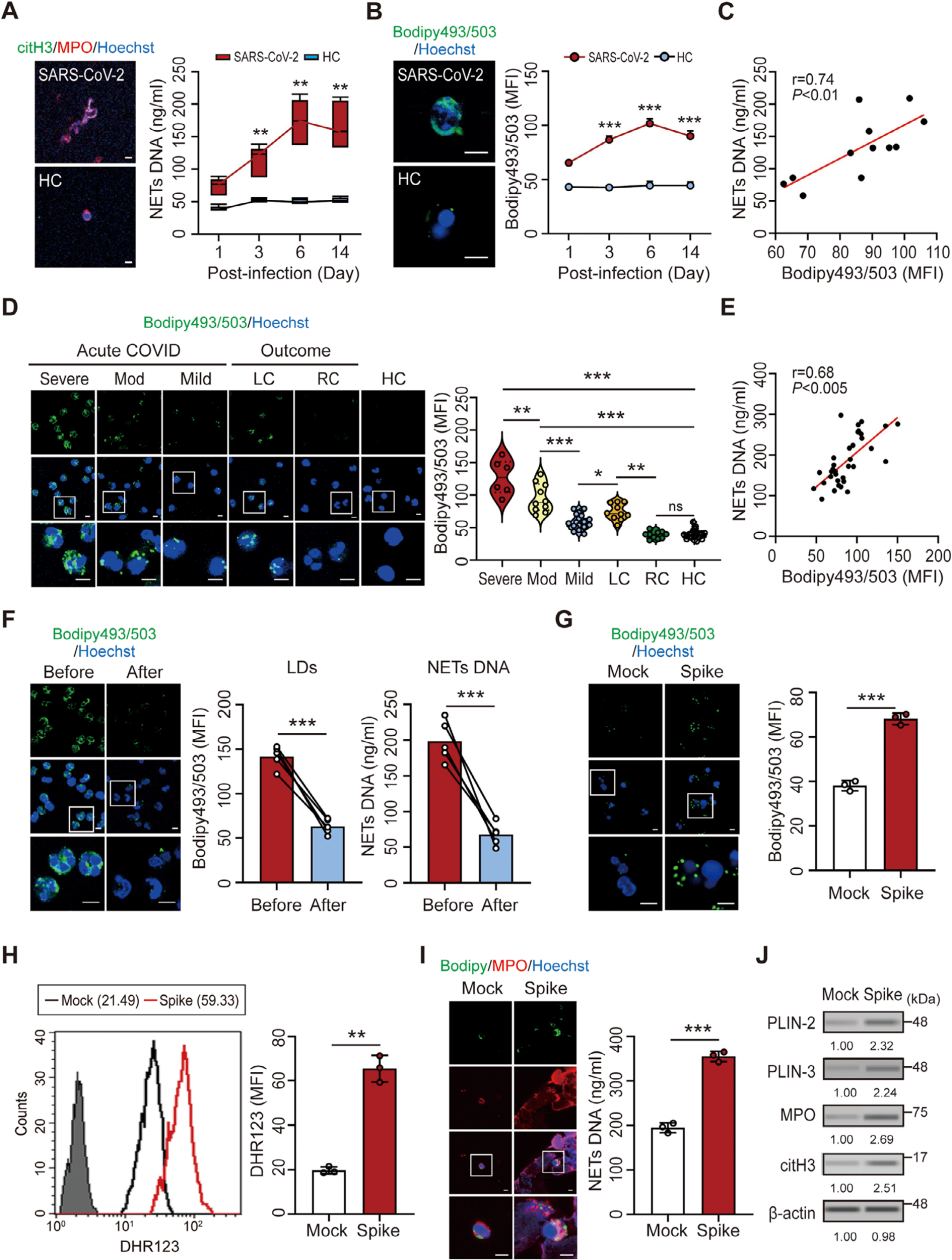
Increased LD accumulation in neutrophils correlates with NET formation in SARS‐CoV‐2‐infected hamsters and patients with COVID‐19. (A–C) Quantification of (A) NET DNA release and (B) LD accumulation in neutrophils from SARS‐CoV‐2‐infected hamsters at 1, 3, 6, and 14 dpi, and from HC. Representative immunofluorescence images show NETs (citH3/MPO) and LDs (BODIPY 493/503) in neutrophils from infected hamsters (top panels) and HC (bottom panels) at 14 dpi. (C) Positive correlation between NET formation and LD levels in neutrophils from infected hamsters. (D) LD levels in neutrophils from patients with different severities of acute COVID‐19 and outcomes, and from HC. (E) Correlation analysis of NET DNA release and LD levels in neutrophils from patients with COVID‐19. (F) LD accumulation and NET DNA release were reduced in patients with acute COVID‐19 following anti‐COVID‐19 therapy. (G–J) Treatment of human neutrophils with recombinant SARS‐CoV‐2 spike protein (4 µg, 4 h) induced (G) LD accumulation, (H) ROS production, and (I, J) NET formation. The scale bars in the immunofluorescence images represent 5 µm. All experiments were performed in triplicate. The data are presented as mean ± SD. Statistical significance was assessed using the Kruskal–Wallis test with Dunn's post hoc correction for multiple groups, and the Mann–Whitney *U* test or unpaired two‐tailed Student's *t*‐test for two‐group comparisons, as described in the Experimental Section. *p < 0.05, **p < 0.01, ***p < 0.005; ns, not significant. Densitometric analysis of immunoblotting is provided in Supporting Information S2. Abbreviations: HC, healthy controls; LC, patients with long COVID; Mod, patients with moderate COVID‐19; RC, patients who recovered from COVID‐19.

To assess neutrophil activation, we measured neutrophil elastase (NE) expression. It was significantly elevated in lung tissues of infected hamsters compared with healthy controls (*p* < 0.005; Figure S1A). We next evaluated NET formation and LD accumulation in neutrophils from infected hamsters (*n* = 6) at multiple time points (1, 3, 6, and 14 days postinfection [dpi]) compared with healthy controls (*n* = 4). Representative immunofluorescence images obtained at 14 dpi showed enhanced citrullinated histone H3 (citH3)/myeloperoxidase (MPO)‐positive NET structures and increased BODIPY 493/503‐positive LDs in infected animals relative to controls (Figure 1A,B, left panels). Quantitative analyses confirmed that both NET levels (*p* < 0.05; Figure 1A, right panel) and LD levels (*p* < 0.005; Figure 1B, right panel) peaked at 6 dpi and declined by 14 dpi. There was a strong positive correlation between LD accumulation and NET formation (*r* = 0.74, *p* < 0.01; Figure 1C).

We further analyzed LD levels in neutrophils from 60 patients who differed regarding the severity of COVID‐19 and outcome (31 females and 29 males), as well as from 20 healthy subjects (11 females and 9 males). There was elevated LD accumulation in patients with acute COVID‐19 with different severities (mean fluorescence intensity [MFI]: severe: *n* = 6, 127.70 ± 26.88, *p* < 0.005; moderate: *n* = 10, 95.62 ± 21.83, *p* < 0.005; mild: *n* = 24, 58.73 ± 11.10, *p* < 0.005; Figure 1D), compared with healthy controls (*n* = 20, 39.88 ± 7.04). Moreover, patients with long COVID (e.g., chronic cough, fatigue, and joint pain; *n* = 10) had significantly higher LD levels (74.35 ± 12.61, *p* < 0.005) than patients who had recovered from COVID‐19 (*n* = 10, 38.77 ± 5.50), while there was no significant difference between patients who had recovered from COVID‐19 and healthy controls. Consistent with the hamster model, LD levels in patients with COVID‐19 correlated positively with NET DNA release (*r* = 0.68, *p* < 0.005, Figure 1E). In patients with acute COVID‐19, both LD accumulation (MFI: 141.81 ± 12.40 vs. 63.29 ± 8.38, *p* < 0.005) and NET DNA release (198.40 ± 28.41 vs. 67.87 ± 15.66, *p* < 0.005) decreased after anti‐COVID‐19 treatment (Figure 1F). Furthermore, treatment of human neutrophils with recombinant SARS‐CoV‐2 spike protein induced LD accumulation (*p* < 0.005, Figure 1G), accompanied by increased reactive oxygen species (ROS) production (*p* < 0.01, Figure 1H) and enhanced NET formation (*p* < 0.005, Figure 1I). These effects were dose‐ and time‐dependent (Figure S2A,B).

Additionally, immunoblotting confirmed upregulation of the LD‐associated proteins perilipin 2 (PLIN2) and PLIN3 in spike protein‐treated neutrophils, together with enhanced MPO and citH3 expression (Figure 1J), indicating that SARS‐CoV‐2 spike protein promotes LD accumulation and NET formation. Consistently, pharmacological inhibition of LD biogenesis using the diacylglycerol *O*‐acyltransferase 1 (DGAT1) inhibitor A922500 or inhibition of ROS generation using diphenyleneiodonium (DPI) markedly reduced spike protein‐induced NET release (Figure S2C–G). Importantly, A922500 suppressed both LD accumulation and NET DNA release in a dose‐dependent manner (Figure S2D,E), providing additional evidence that LD biogenesis directly contributes to NET formation. Together, these results suggest that LD accumulation promotes NET formation through an ROS‐dependent pathway.

### 2.3 pEVs Derived from Patients with COVID‐19 Induce LD Accumulation in Neutrophils through Toll‐Like Receptor 8 (TLR8) Activation

**FIGURE 2:**
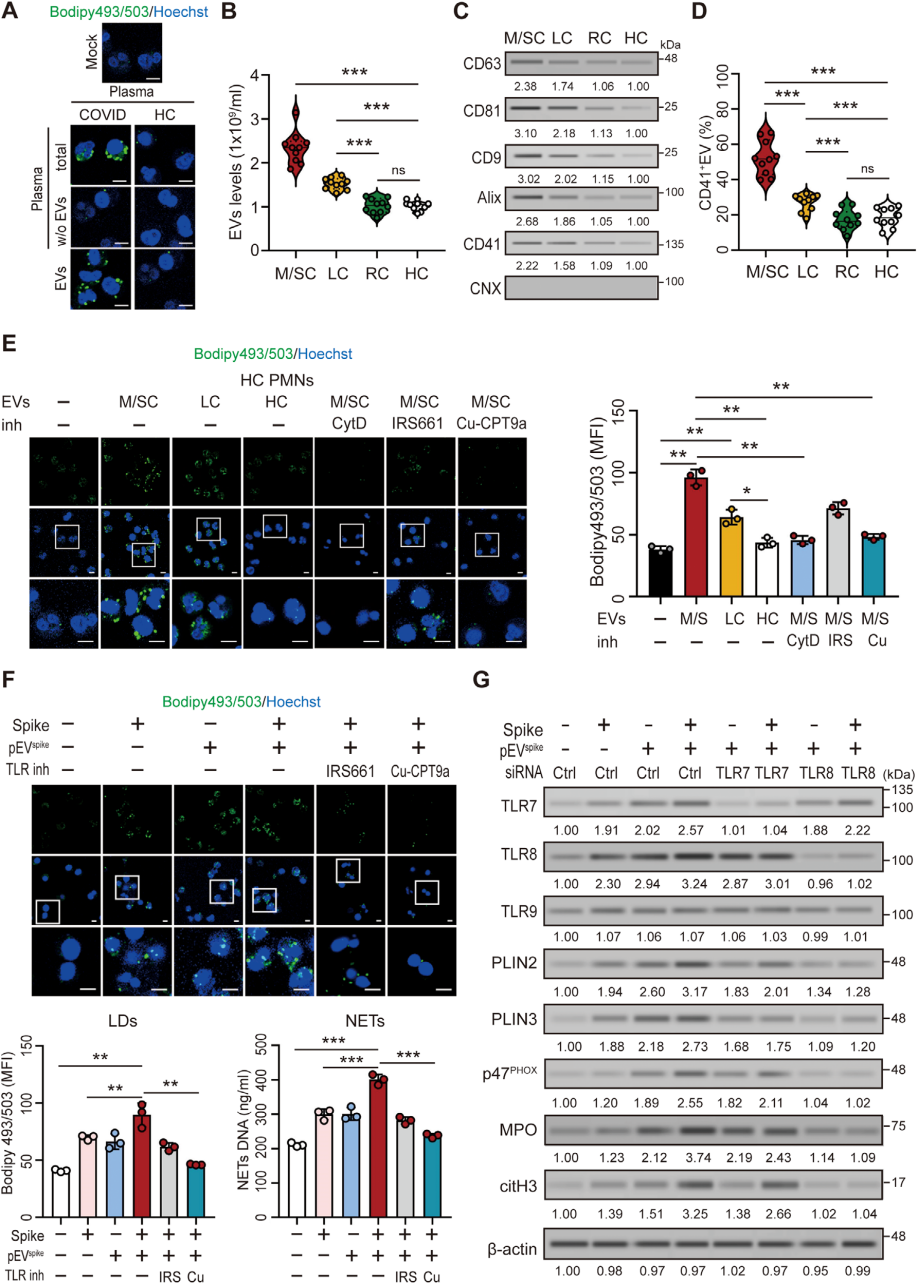
SARS‐CoV‐2‐associated pEVs induce LD accumulation and NET formation in neutrophils via TLR8 activation. (A) Representative images of LD accumulation (BODIPY 493/503, green) in neutrophils treated with plasma, EV‐depleted plasma, or isolated plasma EVs from patients with COVID‐19 and HC. Quantification of LD levels is shown in Figure S3B. (B) Plasma EV concentrations were measured by ELISA in M/SC, RC, and HC. (C) Immunoblotting of EVs showed elevated exosomal (CD63, CD81, CD9, and Alix) and platelet (CD41) markers in M/SC and LC compared with RC and HC. (D) Flow cytometry showed increased CD41⁺CD63⁺ pEVs in plasma from M/SC or LC compared with RC and HC. (E) EVs derived from patients with COVID‐19 increased LD accumulation in human neutrophils, primarily through cellular uptake and TLR8 activation. (F) Recombinant SARS‐CoV‐2 spike protein and pEV^spike^ synergistically induced LD accumulation and NET formation in neutrophils via TLR8 activation. (G) TLR8 knockdown abolished spike protein– and pEV^spike^‐ induced LD accumulation and NET formation in neutrophils. The scale bars in the immunofluorescence images represent 5 µm. All experiments were performed in triplicate. The data are presented as mean ± SD. Statistical significance was assessed using the Kruskal–Wallis test with Dunn's post hoc correction for multiple groups, and the Mann–Whitney *U*‐test or unpaired two‐tailed Student's *t*‐test for two‐group comparisons, as described in the Experimental Section. *p < 0.05, **p < 0.01, ***p < 0.005. Densitometric analysis of immunoblotting is provided in Supporting Information S2. Abbreviations: CNX, calnexin; Cu, Cu‐CPT9a; Cyt D, cytochalasin D; HC, healthy controls; IRS, IRS661; LC, patients with long COVID; M/SC, patients with moderate/severe COVID‐19; RC, patients who had recovered from COVID‐19.

We previously demonstrated that circulating EVs from patients with COVID‐19 enhance NET formation [18]. Negative‐staining transmission electron microscopy (TEM) and nanoparticle tracking analysis (NTA) confirmed that the isolated plasma EVs exhibited the typical cup‐shaped morphology and a predominant size range of 100–150 nm (Figure S3A). To further define their functional contribution, we incubated neutrophils from healthy donors with plasma, EV‐depleted plasma, or isolated EVs from either patients with COVID‐19 or healthy controls for 4 h. Neutrophils treated with COVID‐19 plasma or its isolated EVs displayed pronounced LD accumulation (Figure 2A and Figure S3B) and increased NET DNA release (Figure S3C), whereas EV depletion markedly reduced this effect. In contrast, neither healthy control plasma nor EVs derived from healthy controls induced appreciable LD accumulation or NET formation.

We next analyzed plasma EV levels across patients with COVID‐19 with varying disease severities and clinical outcomes. Using enzyme‐linked immunosorbent assay (ELISA), we observed that patients with moderate to severe COVID‐19 had significantly elevated EV levels (2.34 ± 0.36 × 10^9^ EV particles/mL, *p* < 0.005, Figure 2B) compared with healthy controls (1.02 ± 0.10 × 10^9^ EV particles/mL), consistent with previous findings [18]. Additionally, patients with long COVID also exhibited elevated EV levels (1.53 ± 0.13 × 10^9^ EV particles/mL, *p* < 0.005) relative to patients who had recovered from COVID‐19 (1.03 ± 0.17 × 10^9^ EV particles/mL) and healthy controls. Immunoblotting revealed that EVs from patients with moderate to severe COVID‐19 or long COVID expressed higher levels of exosome markers (CD63, CD81, CD9, and Alix) and the platelet‐specific marker CD41 compared with samples from patients who had recovered from COVID‐19 or healthy controls, whereas the endoplasmic reticulum marker calnexin (CNX) was absent in all EV preparations, confirming the purity of EV isolates (Figure 2C). Flow cytometry confirmed a significant increase in the percentage of CD41^+^CD63^+^ pEVs in patients with moderate to severe COVID‐19 (52.36% ± 9.76%, *p* < 0.005, Figure 2D) or long COVID (27.10% ± 4.77%, *p* < 0.005) compared with healthy controls (18.60% ± 5.48%) and patients who had recovered from COVID‐19 (17.27% ± 5.78%).

Human neutrophils incubated with EVs from patients with moderate to severe COVID‐19 or long COVID exhibited increased LD accumulation (Figure 2E), accompanied by elevated ROS production (Figure S3D) and NET DNA release (*p* < 0.05; Figure S3E). These effects were absent in neutrophils exposed to EVs from healthy controls. Notably, EVs from patients with moderate to severe COVID‐19 induced greater LD accumulation than those from patients with long COVID. Because the endocytosis inhibitor cytochalasin D (CytD) abolished these effects, EV uptake was required, suggesting that COVID‐19 pEVs deliver intracellular signals that regulate LD metabolism. Based on our previous findings that pEVs activate neutrophil TLR7/8 signaling to promote NET formation [18], we next examined whether the same pathway mediates LD accumulation. Treatment with the TLR7 inhibitor IRS661 partially reduced pEV‐induced LD accumulation, whereas the TLR8‐specific inhibitor Cu‐CPT9a almost completely abolished this effect (Figure 2E and Figure S3B,C), indicating that TLR8 plays a predominant role in mediating EV‐induced LD accumulation, ROS generation, and NET release in neutrophils.

We further investigated whether spike protein‐triggered platelet EVs (pEV^spike^) could elicit similar effects. pEV^spike^ refers to EVs secreted by spike protein‐stimulated platelets and lacking surface spike protein, as confirmed by immunoblotting [18]. As shown in Figure 2F, both spike protein and pEV^spike^ independently induced LD accumulation and NET formation. Remarkably, their combination synergistically enhanced both responses, which was nearly abolished by TLR8 inhibition with Cu‐CPT9a (Figure 2F) or TLR8 knockdown (Figure 2G). These findings suggest that pEV^spike^ may carry single‐stranded RNAs, such as tsRNAs, that activate TLR8 in neutrophils, thereby promoting LD accumulation and NET formation.

### 2.4 Spike Protein and pEV^spike^ Induce Mammalian Target of Rapamycin (mTOR) Activation to Inhibit Lipophagy in Neutrophils

**FIGURE 3:**
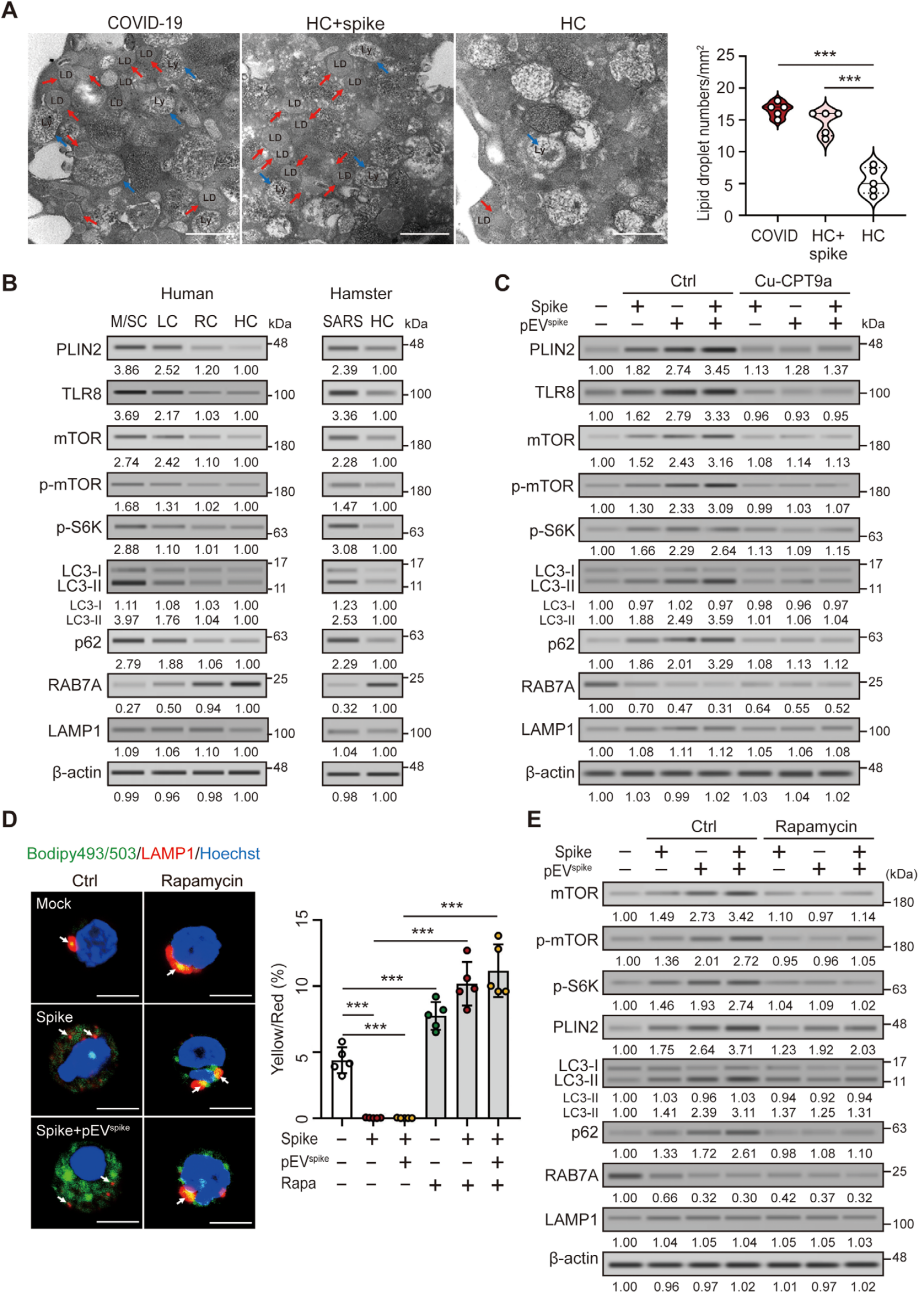
Spike protein and pEV^spike^ impair lipophagy in neutrophils via TLR8–mTOR activation. (A) TEM images showing markedly increased lipid droplets (LDs; red arrows) in neutrophils from patients with COVID‐19 and in spike protein‐treated healthy neutrophils, with LDs frequently adjacent to lysosomes (Ly; blue arrows), indicative of impaired lipophagic processing. Scale bar: 500 nm. (B) Immunoblot analysis of neutrophils from patients with moderate/severe COVID‐19, long COVID, recovered patients, and SARS‐CoV‐2‐infected hamsters showing increased expression of TLR8, total and phosphorylated mTOR (Ser2448), phosphorylated S6K (Thr389), LC3‐II, p62, and PLIN2, together with reduced RAB7A, consistent with impaired lipophagy. (C) Spike protein or pEV^spike^ alone induced similar autophagy defects, whereas cotreatment synergistically enhanced TLR8 activation, mTOR signaling, LC3‐II/p62 accumulation, and RAB7A suppression. These effects were largely reversed by the TLR8‐specific inhibitor Cu‐CPT9a. (D) Immunofluorescence analysis showing LD accumulation and reduced LD–lysosome (LAMP1⁺) colocalization in spike‐treated neutrophils, further exacerbated by pEV^spike^ co‐treatment. Rapamycin restored LD–lysosome fusion, as reflected by increased yellow puncta. Scale bar: 5 µm. (E) Immunoblotting revealed that rapamycin reversed spike and pEV^spike^‐induced increases in mTOR activation, LC3‐II/p62 accumulation, but had no effect on RAB7A expression. All experiments were performed in triplicate. The data are presented as mean ± SD. Statistical significance was assessed using the Kruskal–Wallis test with Dunn's post hoc correction for multiple groups, and the Mann–Whitney *U*‐test or unpaired two‐tailed Student's *t*‐test for two‐group comparisons, as described in the Experimental Section. ***p < 0.005. Densitometric analysis of immunoblotting is provided in Supporting Information S2. Abbreviations: HC, healthy controls; LC, patients with long COVID; M/SC, patients with moderate/severe COVID‐19; RC, patients who had recovered from COVID‐19.

To dissect the regulatory mechanisms underlying LD accumulation in neutrophils from patients with COVID‐19, we first performed TEM, which revealed an increase in the number and size of LDs in patient‐derived and spike protein‐treated neutrophils, with many LDs located near lysosomes—suggestive of impaired lipophagic flux (Figure 3A). Gene set enrichment analysis (GSEA) revealed altered cellular catabolism, while Kyoto Encyclopedia of Genes and Genomes (KEGG) pathway analysis indicated dysregulation of macroautophagy/autophagy‐related signaling in spike‐treated cells (Figure S4).

To validate these findings, we analyzed lipophagy‐related proteins by immunoblotting. Neutrophils from patients with moderate to severe COVID‐19 or long COVID, as well as from SARS‐CoV‐2‐infected hamsters and spike protein– or pEV^spike^‐treated cells, showed increased expression of TLR8, total and phosphorylated mTOR (Ser2448), together with its downstream effector phosphorylated S6K (Thr389), LC3‐II, and p62, accompanied by elevated PLIN2 and reduced RAB7A levels (Figure 3B,C), consistent with impaired lipophagy and mTOR pathway activation. To further assess autophagic flux, we used THP‐1 cells stably expressing a GFP–RFP–LC3 fusion construct. Spike protein and pEV^spike^ treatments led to the accumulation of yellow puncta, indicating blocked autophagosome–lysosome fusion (Figure S5). Spike protein alone induced similar changes, including upregulation of TLR8, phospho‐mTOR, and phospho‐S6K, with concomitant LC3‐II and p62 accumulation and RAB7A reduction, whereas cotreatment with pEV^spike^ further amplified these effects, suggesting a synergistic inhibition of lipophagy (Figure 3C). Importantly, treatment with the TLR8‐specific inhibitor Cu‐CPT9a reversed most of these changes, except for only partial restoration of RAB7A and persistent PLIN2 elevation, suggesting a TLR8‐independent component. Immunofluorescence staining further demonstrated abundant LD accumulation and reduced LD–lysosome colocalization upon combined spike protein and pEV^spike^ treatment, both of which were rescued by rapamycin (Figure 3D). Immunoblotting confirmed that rapamycin suppressed phosphorylation of mTOR and S6K and reduced LC3‐II and p62 expression, while only modestly affecting RAB7A and PLIN2 (Figure 3E). Together, these results indicate that spike protein and pEV^spike^ impair lipophagy through both TLR8–mTOR activation and RAB7A suppression, promoting persistent LD accumulation in neutrophils.

### 2.5 pEV^spike^ Suppresses RAB7A Expression via tRF‐His‐GTG‐1

**FIGURE 4:**
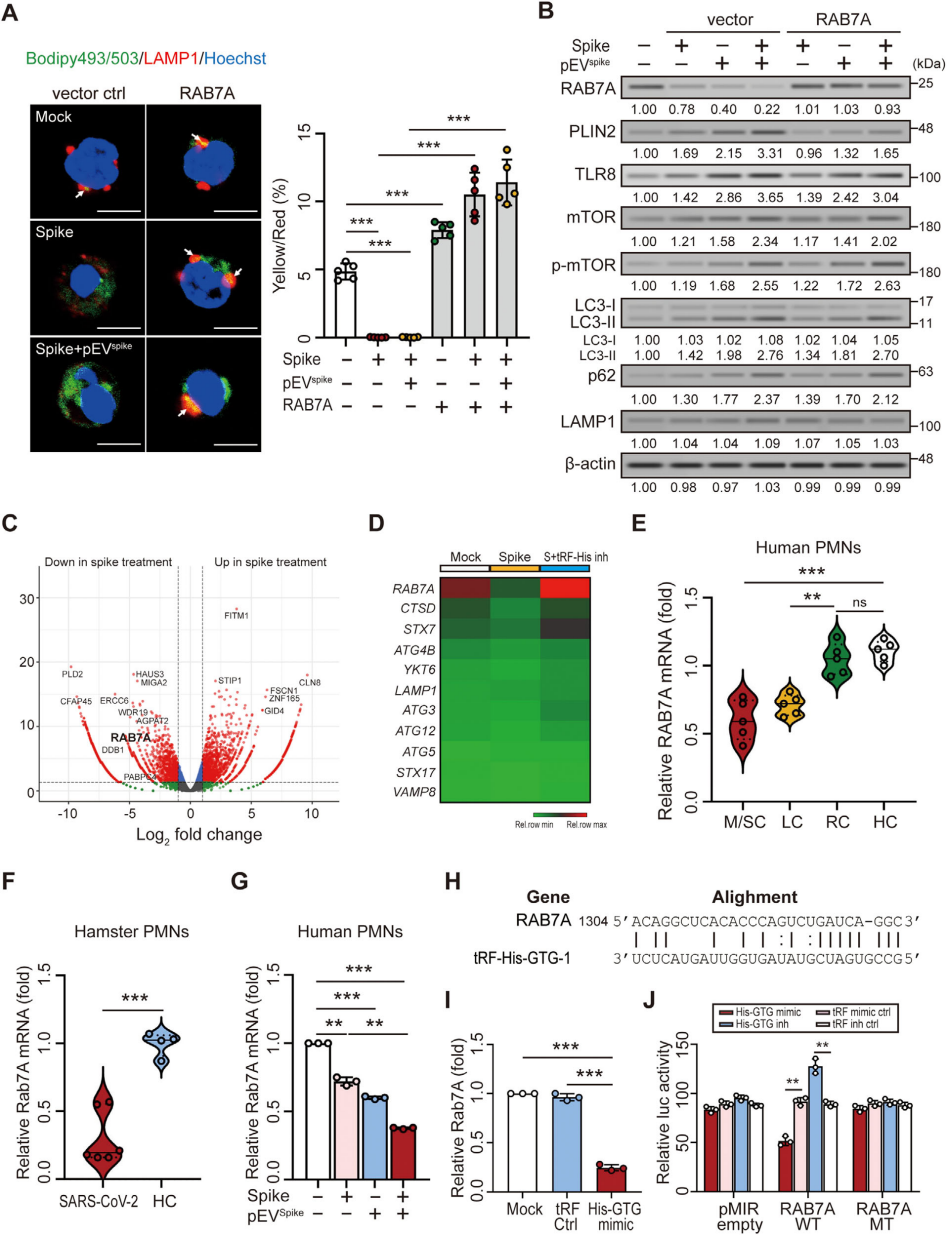
Spike protein and pEV^spike^ suppress RAB7A transcription in neutrophils through tRF‐His‐GTG‐1 targeting. (A) An immunofluorescence assay showed increased colocalization of LDs with lysosomes (LAMP1⁺) in RAB7A‐overexpressing neutrophils, indicating restored lipophagy. (B) Immunoblotting showed that RAB7A overexpression attenuated spike‐ and pEV^spike^‐induced PLIN2 upregulation, supporting a role for RAB7A in limiting LD accumulation. (C) RNA‐seq analysis revealed that spike protein suppressed RAB7A mRNA expression in neutrophils. (D) Heatmap of autophagy‐ and lysosome‐related gene expression in human neutrophils treated with SARS‐CoV‐2 spike protein in the absence or presence of a tRF‐His‐GTG‐1 inhibitor. (E,F) qRT‐PCR analysis confirmed reduced RAB7A expression in neutrophils from (E) patients with COVID‐19 and (F) SARS‐CoV‐2‐infected hamsters, relative to HC. (G) Spike protein and pEV^spike^ synergistically suppressed RAB7A transcription in human neutrophils. (H) Bioinformatic analysis predicted a tRF‐His‐GTG‐1 binding site in the 3′ UTR of RAB7A mRNA. (I) Transfection of a tRF‐His‐GTG‐1 mimic into human neutrophils reduced endogenous RAB7A mRNA expression. (J) Luciferase reporter assays confirmed that tRF‐His‐GTG‐1 directly targets the RAB7A 3′ UTR; mutation of the predicted binding site (MT) abolished this suppressive effect. Scale bars in immunofluorescence images represent 5 µm. All experiments were performed in triplicate. The data are presented as mean ± SD. Statistical significance was assessed using the Kruskal–Wallis test with Dunn's post hoc correction for multiple groups, and the Mann–Whitney *U*‐test or unpaired two‐tailed Student's *t*‐test for two‐group comparisons, as described in the Experimental Section. **p < 0.01, ***p < 0.005; ns, not significant. Densitometric analysis of immunoblotting is provided in Supporting Information S2. Abbreviations: HC, healthy controls; LC, patients with long COVID; M/SC, patients with moderate/severe COVID‐19; MT, mutant; RC, patients who had recovered from COVID‐19; WT, wild type.

To assess the role of RAB7A in spike protein– and pEV^spike^‐induced LD accumulation, we overexpressed RAB7A in neutrophils via plasmid transfection, followed by stimulation with spike protein and pEV^spike^. RAB7A overexpression restored LD–lysosome colocalization and reduced PLIN2 levels, without altering TLR8 or mTOR signaling, indicating a TLR8‐independent function (Figure 4A,B). RNA sequencing (RNA‐seq) analysis revealed that spike protein markedly decreased RAB7A messenger RNA (mRNA) expression in human neutrophils (Figure 4C), consistent with the protein‐level reduction. Heatmap analysis (Figure 4D) showed preferential downregulation of RAB7A, while other lysosome‐ and autophagy‐related genes exhibited relatively minor changes. Quantitative reverse transcription polymerase chain reaction (qRT‐PCR) further confirmed that RAB7A expression was significantly reduced in neutrophils from patients with moderate to severe COVID‐19 or long COVID compared with patients who had recovered from COVID‐19 or healthy controls (Figure 4E), and there was a similar decrease in SARS‐CoV‐2–infected hamsters (Figure 4F). In a cell‐based model, pEV^spike^ synergistically enhanced spike protein‐induced RAB7A suppression (Figure 4G). Consistently, across clinical samples, the hamster model, and primary neutrophils stimulated with spike protein or pEV^spike^, vesicle‐associated membrane protein 8 (VAMP8), lysosome‐associated membrane protein 1 (LAMP1), and RAB5A remained unchanged at both the mRNA and protein levels (Figure S6A–C), highlighting a late‐stage fusion defect rather than broad autophagy‐related gene repression.

Given that COVID‐19‐associated pEVs induce LD accumulation via TLR8 (Figure 2E–G), we hypothesized that pEV^spike^ also suppresses RAB7A through its small RNA cargo. Among candidate tsRNAs, tRF‐His‐GTG‐1 emerged as a highly enriched species in COVID‐19 [21] and systemic lupus erythematosus (SLE) [24, 25]. It promotes NET formation in SLE and binds TLR8 [25]. Because both SLE and COVID‐19 share excessive NET formation and increased pEV release, we reasoned that tRF‐His‐GTG‐1 might also contribute to neutrophil activation in COVID‐19 through a similar pEV–TLR8 signaling mechanism. Bioinformatic analysis further identified a putative seed match for tRF‐His‐GTG‐1 in the 3′ untranslated region (UTR) of human RAB7A mRNA (Figure 4H; GenBank NM_004637). Transfection of a tRF‐His‐GTG‐1 mimic into neutrophils significantly reduced RAB7A mRNA levels (Figure 4I). To validate direct binding, we generated luciferase reporters carrying the wild‐type or a seed‐mutation construct of the RAB7A 3′ UTR. tRF‐His‐GTG‐1 mimicked suppressed luciferase activity in the wild‐type but not the mutant reporter, whereas inhibitors increased activity (Figure 4J). These results confirm RAB7A as a direct post‐transcriptional target of pEV‐delivered tRF‐His‐GTG‐1, acting independently of the TLR8–mTOR axis.

### 2.6 pEVs Derived from Patients with COVID‐19 Carry tRF‐His‐GTG‐1 to Induce LD Accumulation

**FIGURE 5:**
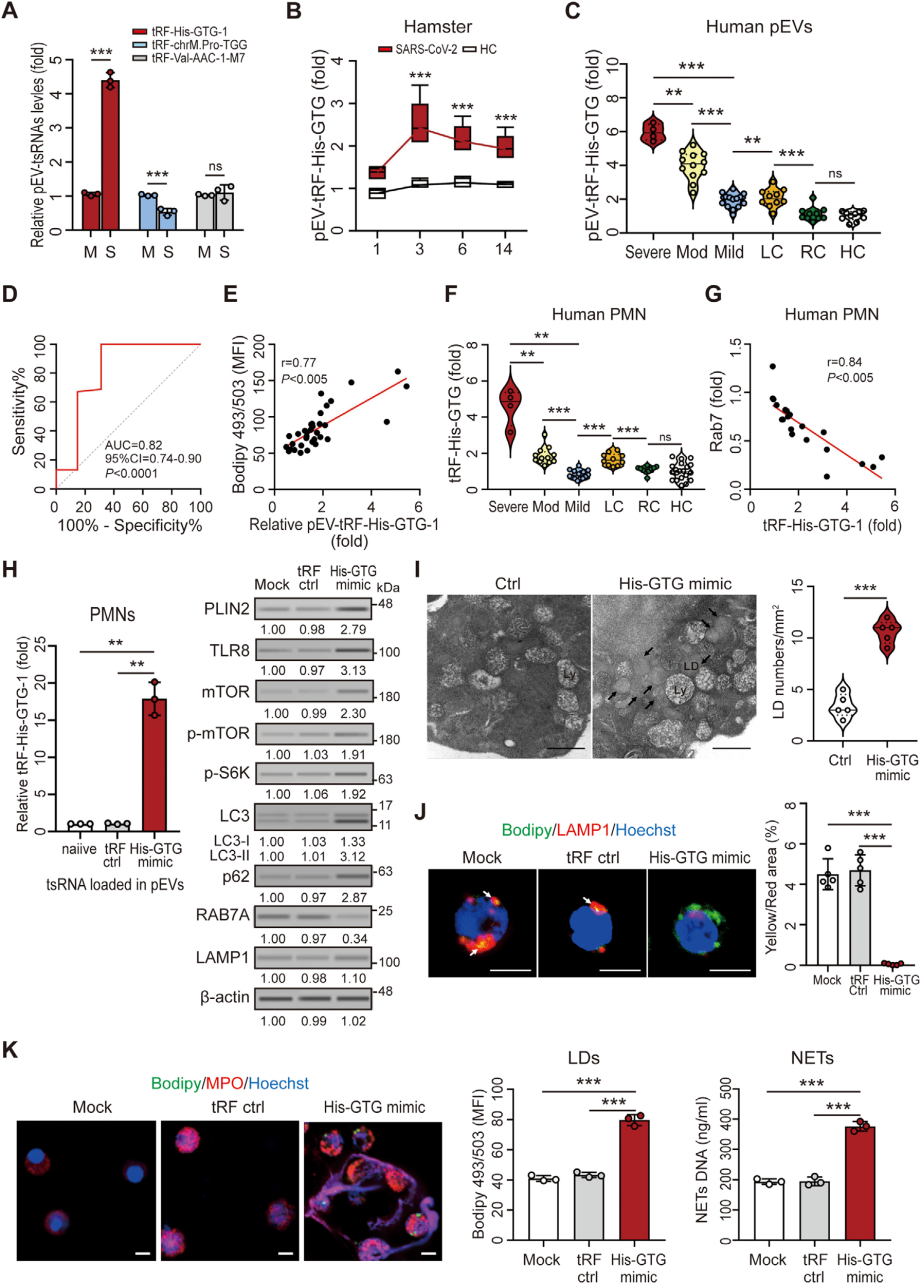
COVID‐19‐associated pEVs carry abundant tRF‐His‐GTG‐1 that impairs lipophagy in neutrophils via TLR8–mTOR activation and RAB7A suppression. (A,B) qRT‐PCR analysis showed elevated tRF‐His‐GTG‐1 levels in pEVs derived from (A) recombinant spike protein‐treated human platelets and (B) SARS‐CoV‐2‐infected hamsters. (C) tRF‐His‐GTG‐1 levels in pEVs from patients with acute COVID‐19 (with different disease severities and clinical outcomes) and HC. (D) Receiver operating characteristic (ROC) curve analysis of tRF‐His‐GTG‐1 levels in pEVs from patients with acute COVID‐19 (with different disease severities and clinical outcomes). (E) tRF‐His‐GTG‐1 levels in pEVs positively correlated with LD accumulation in neutrophils from patients with COVID‐19. (F) tRF‐His‐GTG‐1 levels were elevated in neutrophils from patients with acute COVID‐19 (with different disease severities and clinical outcomes) and LC. (G) tRF‐His‐GTG‐1 levels in human neutrophils negatively correlated with RAB7A expression. (H,I) Electroporation‐mediated loading of tRF‐His‐GTG‐1 mimics into pEVs followed by coculture with human neutrophils resulted in increased intracellular tRF‐His‐GTG‐1 levels (left panel) and altered expression of lipophagy‐associated proteins as assessed by immunoblotting (right panel). (I) TEM showed that tRF‐His‐GTG‐1–overexpressing neutrophils accumulated abundant LDs clustered around lysosomes. (J) Immunofluorescence assay showed that tRF‐His‐GTG‐1 overexpression induced LD accumulation with reduced colocalization with lysosomes (LAMP1⁺), consistent with lipophagy impairment. Scale bars: 5 µm (immunofluorescence), 500 nm (TEM). All experiments were performed in triplicate. The data are presented as mean ± SD. Statistical significance was assessed using the Kruskal–Wallis test with Dunn's post hoc correction for multiple groups, and the Mann–Whitney *U*‐test or unpaired two‐tailed Student's *t*‐test for two‐group comparisons, as described in the Experimental Section. **p < 0.01, ***p < 0.005. ns, nonsignificant. Densitometric analysis of immunoblotting is provided in Supporting Information S2. Abbreviations: HC, healthy controls; LC, patients with long COVID; Mod, patients with moderate COVID‐19; RC, patients who recovered from COVID‐19.

We first examined tRF‐His‐GTG‐1 levels in pEVs from spike protein‐treated human platelets (pEV^spike^) and observed a significant increase compared with mock controls (fold change: 4.41 ± 0.22 vs. 1.05 ± 0.05, *p* < 0.005; Figure 5A), with dose‐ and time‐dependent effects (Figure S7A,B). In SARS‐CoV‐2‐infected hamsters, tRF‐His‐GTG‐1 levels peaked at 3 dpi and declined by 6 dpi (Figure 5B). qRT‐PCR in clinical samples confirmed elevated pEV–tRF‐His‐GTG‐1 in patients with COVID‐19, correlating with disease severity and distinguishing patients with long COVID from patients who had recovered from COVID‐19 (Figure 5C,D). tRF‐His‐GTG‐1 levels in pEVs correlated positively with LD accumulation (*r*  =  0.77, *p* < 0.005; Figure 5E). In addition, there was a consistent increase in tRF‐His‐GTG‐1 in neutrophils from patients with acute COVID‐19 or long COVID (Figure 5F), suggesting active RNA transfer via EVs. Notably, neutrophil tRF‐His‐GTG‐1 levels correlated inversely with RAB7A expression (*r*  =  –0.84; Figure 5G), implicating this tsRNA in post‐transcriptional regulation.

Building on our previous finding that tRF‐His‐GTG‐1 binds TLR8 [25], we tested whether this tsRNA delivered via pEVs could activate TLR8–mTOR signaling and suppress RAB7A expression in neutrophils, thereby inhibiting lipophagy. Specifically, we electroporated tRF‐His‐GTG‐1 or control mimics into purified pEVs and cocultured them with human neutrophils. Mimic‐loaded pEVs increased intracellular tRF‐His‐GTG‐1 levels and induced upregulation of TLR8, phospho‐mTOR, phospho‐S6K, LC3‐II, and p62, alongside RAB7A suppression and PLIN2 elevation (Figure 5H). This dual mechanism—TLR8–mTOR activation and RAB7A repression—was further supported by TEM and an immunofluorescence assay, which showed an increase in LDs, reduced LD–lysosome colocalization (Figure 5I,J), and enhanced NET formation (Figure 5K). These findings demonstrate that tRF‐His‐GTG‐1 impairs lipophagy and promotes neutrophil activation in COVID‐19.

### 2.7 The tRF‐His‐GTG‐1 Inhibitor Restores Lipophagic Flux and Suppresses Neutrophil Activation in COVID‐19

To evaluate the biosafety and target selectivity of the tRF‐His‐GTG‐1 inhibitor, we assessed its cytotoxicity using the CellTiter‐Glo assay (Promega, G7570). We treated neutrophils with increasing concentrations of the inhibitor (50–300 µm) and measured cell viability after 4 h (Figure S7C). There was minimal cytotoxicity at the working concentration (200 µm). We extended the cytotoxicity assay to 24 and 48 h in primary neutrophils, THP‐1 monocytes, and A549 epithelial cells treated with 200 µm inhibitor. There was no significant cytotoxicity in any cell type up to 48 h (Figure S7D). Neutrophils and THP‐1 monocytes showed a time‐dependent decline in viability due to their short ex vivo lifespan, but treatment with the inhibitor had no additional effect, confirming that it has a wide safety margin. Additionally, selectivity profiling by qRT‐PCR revealed that the inhibitor selectively restored RAB7A expression in spike protein‐treated neutrophils without affecting unrelated autophagy regulators (e.g., ATG5), other small GTPases (e.g., RAB5), or nonautophagic regulators, such as phospholipase D2 (PLD2; Figure S7E). Furthermore, RNA‐seq analysis confirmed selective rescue of RAB7A downregulation (Figure 4D).

To exclude off‐target effects, we assessed whether the inhibitor broadly affected TLR8 signaling or other infection‐related tsRNAs. The inhibitor specifically blocked nuclear factor kappa‐light‐chain‐enhancer of activated B cells (NF‐κB) activation induced by tRF‐His‐GTG‐1 and pEV^spike^, without altering responses to canonical TLR8 agonists (ssRNA40 and R848; Figure S7F). qPCR of representative infection‐associated tsRNAs [21] showed no significant changes except for selective reduction of tRF‐His‐GTG‐1 (Figure S7G), confirming its sequence‐specific and pathway‐restricted activity on the TLR8–mTOR–RAB7A axis. These findings indicate that the observed rescue effects are not attributable to global transcriptional changes or off‐target effects; rather, they reflect the specific action of the tRF‐His‐GTG‐1 inhibitor on RAB7A and the TLR8–mTOR axis.

**FIGURE 6:**
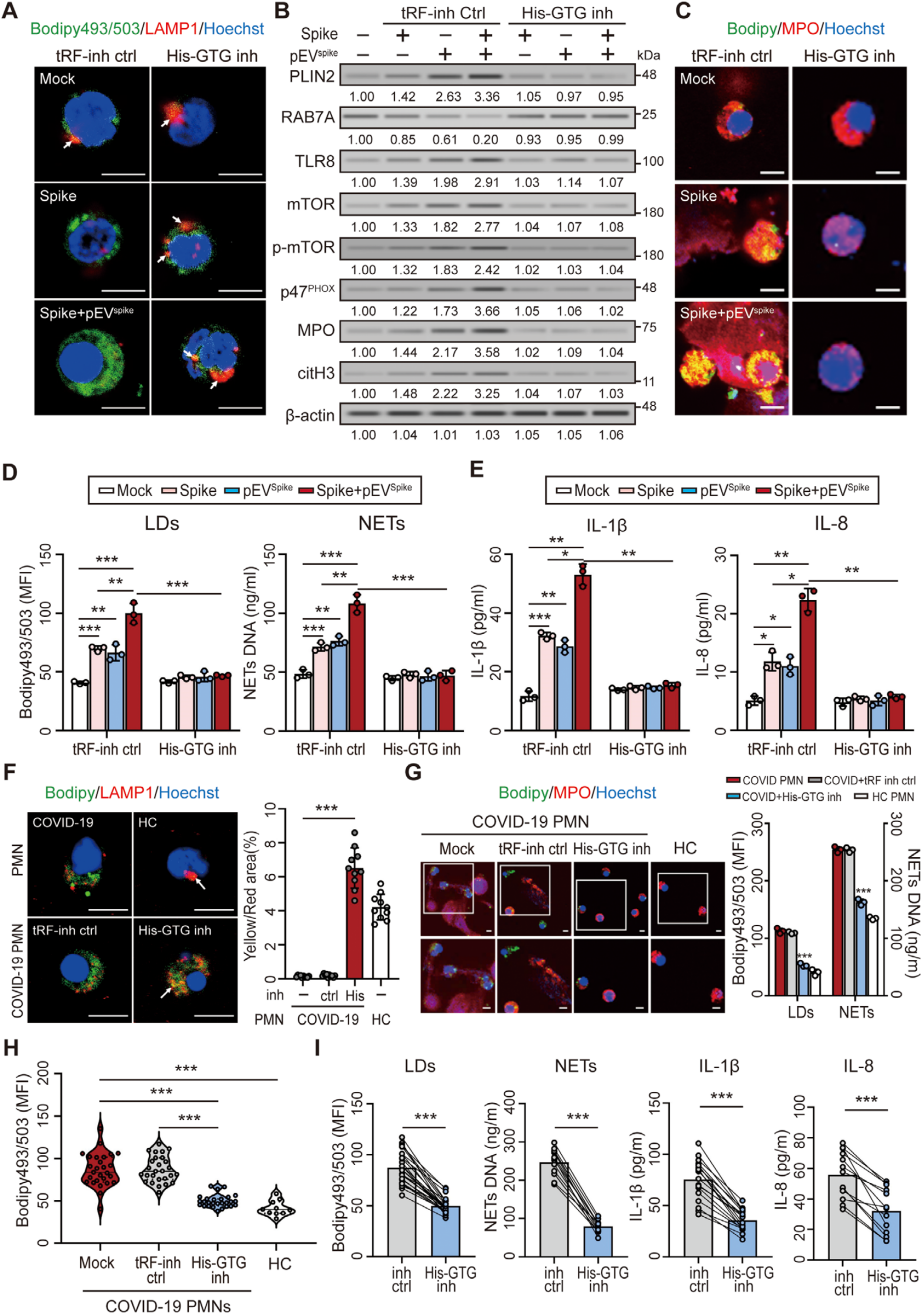
tRF‐His‐GTG‐1 inhibition restores lipophagy and suppresses NET formation and inflammation in COVID‐19 neutrophils. (A) Immunofluorescence analysis showed that treatment with a tRF‐His‐GTG‐1 inhibitor restored LD–lysosome (LAMP1⁺) colocalization, which was impaired by spike protein and pEV^spike^. (B) Immunoblotting revealed that the tRF‐His‐GTG‐1 inhibitor reversed spike protein– and pEV^spike^‐induced RAB7A suppression and TLR8‐mTOR activation, leading to reduced expression of PLIN2, phosphorylated p47^PHOX^, MPO, and citH3. (C–E) The tRF‐His‐GTG‐1 inhibitor suppressed LD accumulation, NET formation, and proinflammatory cytokine production (IL‐1β and IL‐8) induced by spike protein and pEV^spike^, as shown by (C) immunofluorescence and (D,E) quantification by flow cytometry and ELISA. (F–I) Neutrophils isolated from patients with COVID‐19 were treated ex vivo with the tRF‐His‐GTG‐1 inhibitor (200 µm), resulting in (F) enhanced colocalization of LDs and lysosomes (LAMP1⁺), (G) decreased NET DNA release, and (H) reduced LD accumulation. (I) Paired analysis of neutrophils from patients with COVID‐19 (*n* = 27) treated ex vivo with a tRF‐His‐GTG‐1 inhibitor or a control tsRNA inhibitor showed significantly reduced LD levels, NET DNA release, and secretion of IL‐1β and IL‐8. Scale bars in immunofluorescence images represent 5 µm. All experiments were performed in triplicate, and data are presented as mean ± SD. Statistical significance was assessed using the Kruskal–Wallis test with Dunn's post hoc correction for multiple groups, and the Mann–Whitney *U*‐test or unpaired two‐tailed Student's *t*‐test for two‐group comparisons, as described in the Experimental Section. *p < 0.05, **p < 0.01, ***p < 0.005. Densitometric analysis of immunoblotting is provided in Supporting Information S2.

To assess whether the tRF‐His‐GTG‐1 inhibitor restores lipophagy, we analyzed LD–LC3 and LC3–LAMP1 colocalization. Spike protein alone or in combination with pEV^spike^ impaired lipophagic flux, with the combined condition inducing more pronounced suppression—reflecting an elevated pEV burden in severe COVID‐19 and long COVID. Treatment with the inhibitor markedly reversed these effects (Figure S8A,B). Consistently, there was an increase in LD‐containing lysosomes (Figure 6A and Figure S8C). Lysosomal acidity was unaffected (Figure S8D), excluding pH‐related mechanisms. Immunoblotting confirmed that the inhibitor reduced LC3‐II and p62 accumulation, indicating restored autophagic flux (Figure S8E). In contrast, lysosomal inhibition with bafilomycin A1 (BafA1; Figure S8E) or chloroquine (CQ; Figure S9E) did not further increase LC3‐II or p62, confirming that spike protein and pEV^spike^ had already impaired autophagosome–lysosome fusion (Figure S9A–D). These results demonstrate that the inhibitor rescues spike protein– and pEV^spike^‐induced lipophagy defects by restoring autophagosome–lysosome fusion rather than affecting autophagy initiation. Moreover, the tRF‐His‐GTG‐1 inhibitor decreased the levels of PLIN2, phosphorylated p47^PHOX^, and NET‐associated proteins including MPO and citH3, as well as the proinflammatory cytokines interleukin 1beta (IL‐1β) and IL‐8 (Figure 6B–E). Consistent with our previous findings that tRF‐His‐GTG‐1 modulates TLR8‐dependent NF‐κB activation [25], these results suggest that the inhibitor exerts its effects through suppression of TLR8–mTOR and NF‐κB signaling.

We next tested the ex vivo efficacy of the inhibitor in neutrophils from patients with COVID‐19. We incubated neutrophils with either the tRF‐His‐GTG‐1 inhibitor or a control tsRNA inhibitor (both at 200 µm) for 4 h. Compared with healthy controls, patient‐derived neutrophils showed an increase in LDs and impaired LD–lysosome colocalization, both of which were restored by the inhibitor (Figure 6F and Figure S10A). This was accompanied by reduced NET release and cytokine production (Figure 6G–I and Figure S10B–E). These findings support the therapeutic potential of the tRF‐His‐GTG‐1 inhibitor in restoring lipophagy and reducing neutrophil‐driven inflammation in COVID‐19.

## 3 Discussion

SARS‐CoV‐2 spike protein is a central driver of COVID‐19 pathogenesis and long COVID [26, 27], while NETs contribute to tissue injury in acute and chronic inflammation [28] and correlate with COVID‐19 severity [2, 3, 4, 5]. Persistent NET activity has recently been linked to long COVID, with circulating NET markers remaining elevated for months after viral clearance and associating with fatigue, dyspnea, and microvascular injury [5, 29, 30, 31]. Using a long COVID hamster model [23], we observed that although TCID_50_ results indicated viral clearance by 14 dpi, spike protein remained detectable in lung tissue, accompanied by heightened neutrophil activation, perivascular mixed‐cellular infiltration, and type II alveolar epithelial hyperplasia (Figure S1). These findings suggest that residual spike protein may persist after viral RNA clearance and drive sustained NET overactivation, contributing to long COVID‐related chronic inflammation. Mechanistically, NET proteases and histones induce endothelial damage and immunothrombosis, NET‐derived IL‐8 and TGF‐β activate fibroblasts [32, 33], and NET DNA–protein complexes serve as autoantigens [34], collectively establishing a feedback loop that perpetuates chronic inflammation.

Recent evidence indicates that lipid homeostasis is critical for neutrophil function [6]. We observed a positive correlation between LD levels and NET DNA release in COVID‐19 patients and infected hamsters. Spike protein induced LD accumulation, ROS production, and NET formation, all of which were attenuated by pharmacological inhibition of LD biogenesis or ROS generation, suggesting that LD accumulation promotes NET formation through a ROS‐dependent pathway. We previously showed that spike‐activated platelets release pEV^spike^ that enhance NET formation [18], and here we demonstrate that pEV^spike^ synergistically amplifies LD accumulation and NET release, effects almost completely abolished by the TLR8 inhibitor Cu‐CPT9a or TLR8 knockdown.

TLR8, a cytoplasmic ssRNA sensor highly expressed in neutrophils, mediates antiviral innate responses [35]. In this study, pEV^spike^ acted synergistically with spike protein to promote LD accumulation via pEV uptake and TLR8 activation, suggesting that specific ssRNAs within pEVs modulate LD dynamics. Several tsRNAs are upregulated in severe SARS‐CoV‐2 infection [21, 22], and we detected abundant tRF‐His‐GTG‐1 in pEVs—previously shown to bind TLR8 and regulate NET formation in SLE [25]. tRF‐His‐GTG‐1 levels were elevated in pEVs from spike‐treated platelets, SARS‐CoV‐2–infected hamsters, and patients with COVID‐19 or long COVID. Spike protein induced dose‐ and time‐dependent loading of tRF‐His‐GTG‐1 into pEVs, and tRF‐His‐GTG‐1‐enriched pEVs triggered LD accumulation in neutrophils.

Lipophagy selectively degrades intracellular LDs. Neutrophils from COVID‐19 patients or those exposed to spike protein or tRF‐His‐GTG‐1‐carrying pEVs exhibited increased LD–lysosome proximity by TEM, suggesting that spike protein or tRF‐His‐GTG‐1 impairs lipophagy and thereby drives LD accumulation. Elevated tRF‐His‐GTG‐1 levels in patient‐derived pEVs correlated with disease severity, indicating its potential as both a biomarker of immunometabolic dysregulation and a candidate for nucleic acid‐based intervention. Additional studies are required to validate these observations.

SARS‐CoV‐2‐associated ssRNAs activate neutrophils through TLR8 [36]. Our results confirmed that spike protein, pEV^spike^, and tRF‐His‐GTG‐1‐carrying pEVs upregulate mTOR via TLR8, leading to LC3‐II and p62 accumulation. This effect was abolished by Cu‐CPT9a or rapamycin, indicating that spike protein or pEV^spike^ may elevate specific ssRNAs (e.g., tRF‐His‐GTG‐1) to activate TLR8 and drive mTOR expression. mTOR suppresses autophagy through ULK1 phosphorylation and impairs autophagosome formation and lysosomal function, thereby promoting LD accumulation [37, 38, 39, 40]. SARS‐CoV‐2 ORF7a further activates autophagy via the AKT–mTOR–ULK1 pathway and blocks autophagosome–lysosome fusion to enhance viral replication [41], and mTOR hyperactivation facilitates SARS‐CoV‐2 replication [42, 43, 44]. tRF‐His‐GTG‐1 binds TLR8 and modulates NF‐κB [25], and NF‐κB directly regulates mTOR [45], supporting a network linking pEV‐borne tsRNAs to autophagy disruption. Consistently, pEV^spike^ augmented spike‐induced LC3‐II and p62 accumulation, suggesting synergistic inhibition of autophagic flux. Notably, BafA1 and CQ did not further increase LC3‐II or p62 in cotreated cells, indicating maximal blockade of autophagosome–lysosome fusion. Further work is needed to define how TLR8–mTOR signaling regulates lipophagy during SARS‐CoV‐2 infection.

In addition to TLR8–mTOR‐dependent signaling, we found that spike protein, pEV^spike^, and tRF‐His‐GTG‐1‐enriched pEVs suppress RAB7A in neutrophils. This downregulation was not reversed by TLR8 or mTOR antagonists, indicating a TLR8–mTOR‐independent mechanism. Reduced RAB7A mRNA and protein levels were confirmed in neutrophils from SARS‐CoV‐2–infected hamsters and patients with COVID‐19, consistent with a recent clinical report [46]. Spike‐induced tRF‐His‐GTG‐1 directly targets the RAB7A 3′ UTR, thereby repressing its expression. This repression impairs lipophagy and promotes LD accumulation, contributing to increased NET formation. RAB7 is essential for autophagosome and endosome maturation [47] and a central mediator of lipophagy and lipid homeostasis [48, 49]. Walia et al. reported that SARS‐CoV‐2 ORF3a inhibits RAB7 GTP hydrolysis, blocks endosome–lysosome fusion, and enhances viral production [50], suggesting that viral proteins may differentially modulate RAB7 activity. Additional studies are required to elucidate how SARS‐CoV‐2 alters RAB7‐dependent lipophagy.

Collectively, these results demonstrate a dual mechanism through which tRF‐His‐GTG‐1 regulates neutrophil lipophagy and NET formation via TLR8–mTOR activation and RAB7A suppression. tRF‐His‐GTG‐1 functions upstream to activate TLR8–mTOR while concurrently repressing RAB7A. The TLR8–mTOR pathway drives LD biogenesis and NET formation, whereas RAB7A loss further impairs lipophagy. Inhibition of tRF‐His‐GTG‐1 restores RAB7A and reduces TLR8 and mTOR phosphorylation, supporting a dual regulatory loop. Importantly, the inhibitor provides greater functional rescue than TLR8/mTOR blockade or RAB7A overexpression, underscoring its integrative upstream role. Thus, TLR8–mTOR initiates LD accumulation and NET formation, while tRF‐His‐GTG‐1–mediated RAB7A suppression sustains lipophagy blockade and neutrophil hyperactivation.

In addition to pEVs, complement activation, proinflammatory cytokines, and direct viral interaction have also been implicated in NET formation during COVID‐19 [3, 4, 51]. Growing evidence indicates that SARS‐CoV‐2 infection, IL‐6, TNF‐α, and complement C5a converge on platelet activation to trigger pEV release, thereby amplifying neutrophil activation [52]. Consistently, EV depletion from patient plasma markedly reduced LD accumulation and NET formation, while purified pEV fully recapitulated the effects of COVID‐19 plasma or spike stimulation. Moreover, inhibition of the pEV–TLR8–mTOR pathway largely abolished LD accumulation and NET formation, supporting the conclusion that although cytokine and complement pathways contribute to baseline activation, pEV‐mediated tsRNA delivery is the dominant driver of immunometabolic dysregulation and excessive NET formation in COVID‐19.

The high mutation rate of SARS‐CoV‐2 limits the durability of direct‐acting antivirals, increasing interest in host‐directed therapies [53]. Nucleic acid therapeutics offer precise in vivo modulation and rapid development [54, 55]. We showed that the tRF‐His‐GTG‐1 inhibitor suppresses spike‐ and pEV^spike^‐induced TLR8–mTOR signaling and restores RAB7A, rescuing lipophagy, reducing LD accumulation, and limiting NET release. As tRF‐His‐GTG‐1 activates NF‐κB through TLR8 [25], the inhibitor also reduced IL‐1β and IL‐8 secretion in neutrophils from COVID‐19 patients, supporting its potential as an RNA‐based immunotherapeutic.

While this pilot study offers novel mechanistic insights, it has several limitations that warrant further investigation. First, the number of enrolled patients with COVID‐19 or long COVID was relatively small. Additional larger, longitudinal studies are required to validate our findings. Nevertheless, the statistical analyses were robust, as both parametric and nonparametric tests yielded consistent results, and post hoc power analysis confirmed sufficient power to detect meaningful differences despite the small cohort size. The limited number of severe COVID‐19 cases reflects restricted ICU admissions during the study period; however, consistent clinical and molecular trends across severity groups, together with supportive time‐course data from the hamster infection model and acute patient follow‐up, reinforce the validity of our conclusions. Second, the human cohort was cross‐sectional, but we partially mitigated this limitation by incorporating dynamic data from the hamster model and longitudinal monitoring in patients with acute COVID‐19. These data revealed consistent temporal trends in LD accumulation, NET formation, and tRF‐His‐GTG‐1 expression. Future large‐scale longitudinal cohorts are warranted to confirm these temporal relationships. Third, we did not perform in vivo validation of tRF‐His‐GTG‐1 inhibitor efficacy, although we demonstrated potent ex vivo inhibitory effects in neutrophils from patients with COVID‐19. Despite these limitations, we uncovered a molecular mechanism linking pEVs, aberrant LD accumulation, and excessive NET formation to the pathogenesis of severe COVID‐19 and long COVID. These findings suggest the potential therapeutic value of targeting EV‐mediated RNA signaling to modulate neutrophil‐driven inflammation in COVID‐19.

**FIGURE 7:**
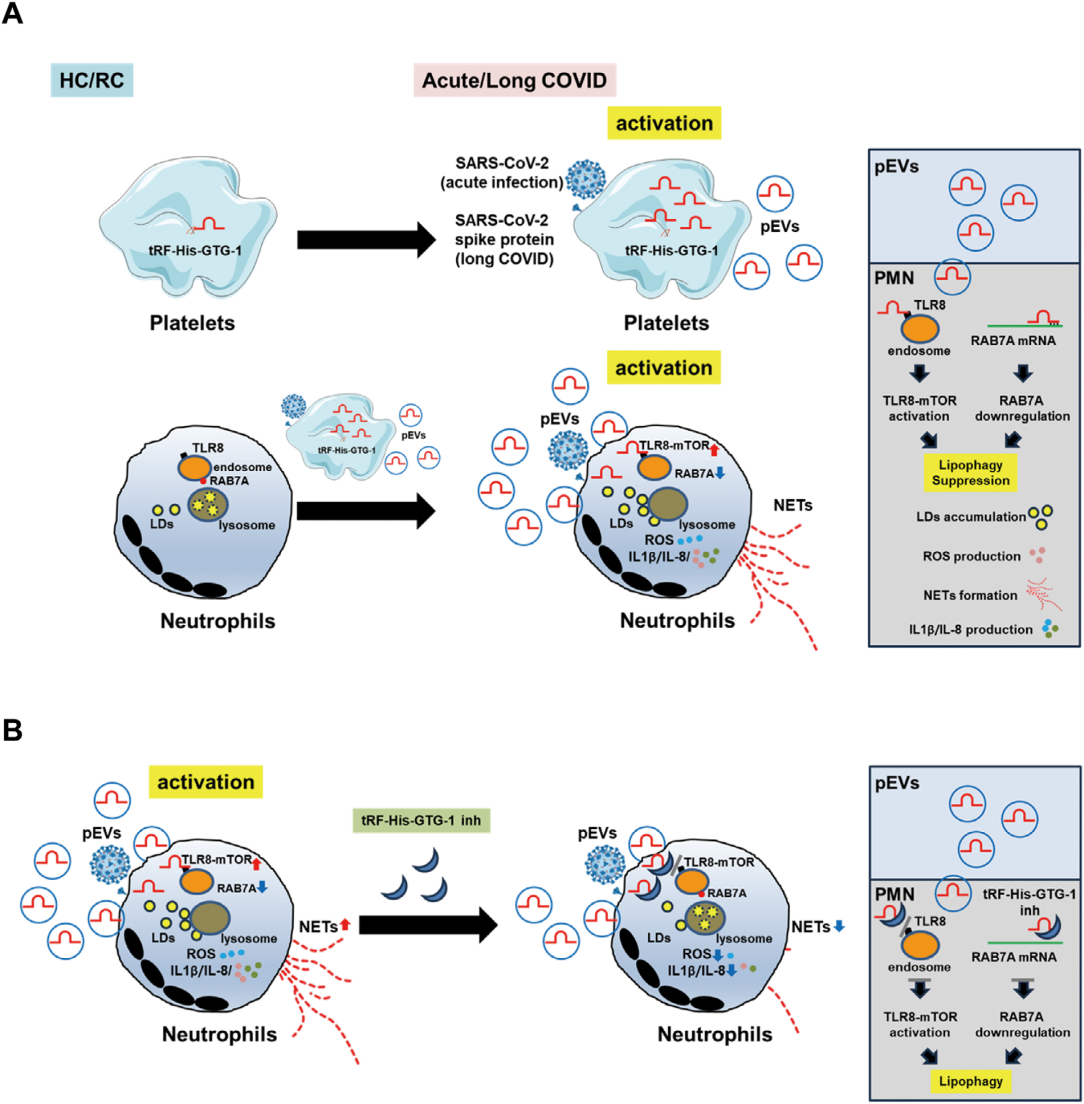
Proposed model illustrating how pEVs and tRF‐His‐GTG‐1 regulate LD metabolism, NET formation, and proinflammatory cytokine production in neutrophils during acute COVID‐19 and long COVID. (A) In HC and RC, resting platelets release minimal pEVs containing tRF‐His‐GTG‐1. During acute and long COVID, SARS‐CoV‐2 spike protein induces platelet activation and promotes the release of tRF‐His‐GTG‐1‐enriched pEVs. These pEVs are taken up by neutrophils, wherein tRF‐His‐GTG‐1 activates endosomal TLR8 signaling and directly suppresses RAB7A mRNA expression. TLR8 activation upregulates mTOR and inhibits lipophagy, while RAB7A downregulation impairs LD–lysosome fusion. These effects collectively promote LD accumulation, ROS production, NET release, and increased secretion of IL‐1β and IL‐8. (B) Treatment with a tRF‐His‐GTG‐1 inhibitor restores RAB7A expression and suppresses TLR8–mTOR signaling, thereby reactivating lipophagy, facilitating LD clearance, and reducing NET formation and inflammatory cytokine production in neutrophils. Abbreviations: HC, healthy controls; LDs, lipid droplets; NETs, neutrophil extracellular traps; pEVs, platelet‐derived extracellular vesicles; RC, patients who had recovered from COVID‐19.

Our study highlights the interplay between lipophagy, lipid metabolism, and neutrophil immune responses during SARS‐CoV‐2 infection. We observed significantly elevated levels of pEVs, LDs, and NETs in patients with severe COVID‐19 or long COVID compared with those with mild disease. We showed that pEV^spike^ and spike protein synergistically impair lipophagy, leading to LD accumulation and hyperactive NET formation, thereby exacerbating immune pathology (Figure 7A). We identified tRF‐His‐GTG‐1 carried by pEV^spike^ as a key regulator that activates TLR8–mTOR signaling and suppresses RAB7A expression, thereby impairing lipophagy. Inhibition of tRF‐His‐GTG‐1 restored RAB7A levels, suppressed TLR8–mTOR signaling, and reduced LD accumulation, NET formation, and proinflammatory cytokine production (Figure 7B). These findings establish lipophagy as a critical immunometabolic checkpoint and suggest that targeting the TLR8–mTOR–RAB7A–lipophagy axis with nucleic acid therapeutics may represent a promising strategy against COVID‐19. Further validation in larger, longitudinal cohorts is warranted to confirm these findings and to support clinical translation.

## 4 Experimental Section

### 4.1 Subjects

In this study, a total of 60 patients with COVID‐19 and 20 healthy controls were enrolled at Taichung Veterans General Hospital. COVID‐19 diagnosis was confirmed by RT‐PCR detection of SARS‐CoV‐2 RNA. Patients with acute infection were classified according to disease severity during hospitalization: (1) severe, requiring ICU admission, invasive mechanical ventilation, or showing bilateral pulmonary infiltrates; (2) moderate, hospitalized patients not meeting severe criteria; and (3) mild, individuals with minor symptoms not requiring hospitalization. Recovered COVID‐19 patients were defined as individuals who had fully recovered for more than 30 days, exhibited no residual symptoms, and tested negative for viral RNA at the time of sampling. Long COVID was defined as persistent, relapsing, or new symptoms lasting ≥30 days after infection [56]. Healthy controls were age‐ and sex‐matched volunteers with no history of SARS‐CoV‐2 infection, autoimmune or metabolic diseases, and not receiving immunomodulatory therapy. Exclusion criteria included autoimmune diseases, malignancy, bleeding disorders, current anticoagulant or immunomodulatory treatment, and pregnancy. This study was conducted in accordance with the Declaration of Helsinki and was approved by the Institutional Review Board of Taichung Veterans General Hospital (CE21403B). All procedures followed institutional guidelines, and written informed consent was obtained from all participants.

### 4.2 Animals

Six‐ to eight‐week‐old male and female Syrian golden hamsters (*Mesocricetus auratus*) were purchased from the National Laboratory Animal Center (Taiwan) and housed under ABSL‐3 conditions for 2 weeks prior to experiments for acclimatization. Ten hamsters were randomly assigned to two groups: SARS‐CoV‐2 infection (*n* = 6; 3 male, 3 female) and noninfection (*n* = 4; 2 male, 2 female). Animals were housed in individually ventilated cages under standard conditions (21°C ± 2°C, 40%–50% relative humidity, 12:12 h light–dark cycle) with ad libitum access to food and water. Infection was performed by intranasal inoculation with 30 µL of SARS‐CoV‐2/JN‐1 (1 × 10⁴ TCID_50_/mL) or phosphate‐buffered saline (PBS, Sigma‐Aldrich, P2272) under general anesthesia [23]. Blood samples were collected at 1, 3, and 6 dpi. At 14 dpi, animals were euthanized and lungs were harvested and fixed in 4% paraformaldehyde (Sigma‐Aldrich, 47608) for histological analysis. All procedures adhered to the Taiwan Council of Agriculture Guide for the Care and Use of Laboratory Animals and were approved by the Institutional Animal Care and Use Committee of Taichung Veterans General Hospital (La‐1132078).

### 4.3 Cell Culture

HEK293T cells (ATCC, CRL‐3216, RRID: CVCL_0063) were obtained from the American Type Culture Collection and maintained in Dulbecco's modified Eagle's medium (DMEM; Invitrogen) supplemented with 10% fetal bovine serum (FBS) at 37°C in 5% CO_2_. The THP‐1 RFP–GFP–LC3 stable cell line (THP1‐Difluo hLC3 cells, InvivoGen, thpdf‐hlc3) was cultured in RPMI‐1640 medium at 37°C in 5% CO_2_, according to the manufacturer's instructions. Mycoplasma contamination was routinely screened using the MycoAlert Mycoplasma Detection Kit (Lonza, LT07‐418). All cell lines used in this study were maintained for fewer than 20 passages.

An RRID for THP1‐Difluo hLC3 is currently unavailable. This cell line, commonly used for LC3‐based autophagic flux assays, does not impact data interpretation or study conclusions.

### 4.4 Neutrophils Isolation

Neutrophils were isolated from venous blood using Polymorphprep (Axis‐Shield, 1895) according to the manufacturer's instructions. Whole blood was centrifuged at 500 *× g* for 30 min at 25°C with slow deceleration. The polymorphonuclear leukocyte layer was collected and incubated with ammonium‐chloride‐potassium (ACK) lysing buffer (Thermo Fisher Scientific, A10492) for 5 min, followed by centrifugation at 500 *× g* for 5 min at 25°C. The resulting cell pellets were resuspended in 10 mL of Hank's Balanced Salt Solution (HBSS; Sigma‐Aldrich, H6648), centrifuged again, and finally resuspended in 1 mL HBSS for downstream assays.

### 4.5 Transmission Electron Microscopy

Human neutrophils were prepared for transmission electron microscopy (TEM) as previously described [57]. Images were acquired from three independent samples per group, with 5–7 fields imaged per sample, using a transmission electron microscope (HT7700; Hitachi, Japan).

### 4.6 EVs Isolation and Quantification

Plasma or culture supernatants were centrifuged at 350 *× g* for 10 min at 4°C to remove cell debris. For EV enrichment, 2.5 mL of supernatant was diluted with 7.5 mL of PBS (Thermo Fisher Scientific, 70011044) and concentrated using Amicon Ultra‐0.5 centrifugal filters (100 kDa cutoff; Millipore, UFC5030) at 3,000 *× g* for 30 min at 4°C. The 100 µL retentate was then diluted in 1.4 mL PBS, centrifuged at 10,000 *× g* for 30 min at 4°C, and ultracentrifuged at 120,000 *× g* for 90 min at 4°C. Pellets were resuspended in 50 µL PBS and stored at −80 °C until further use. EV identity and purity were confirmed by TEM [18], immunoblotting, and flow cytometry. EV concentration was quantified using a CD63‐direct ELISA kit (System Biosciences, EXOEL‐CD63A‐1) according to the manufacturer's protocol.

### 4.7 Platelets Isolation and pEV^spike^ Preparation

Platelets were isolated from peripheral blood by centrifugation at 230 *× g* for 15 min at 24°C, followed by 1000 *× g* for 10 min. Pellets were resuspended in Tyrode's buffer (Sigma‐Aldrich, T2397) containing acid citrate dextrose (1:6 vol/vol; Sigma‐Aldrich, C3821) and 1 µm prostaglandin I_2_ (PGI_2_; Sigma‐Aldrich, P6188), then centrifuged again at 1000 *× g* for 10 min. The final platelet suspension was prepared in Tyrode's buffer supplemented with 1 µm PGI_2_ and 0.04 U/mL apyrase (Sigma‐Aldrich, A6535) and incubated on a shaker at 24°C. Prior to stimulation, platelets were centrifuged again at 1000 *× g* for 10 min and resuspended in fresh Tyrode's buffer. To prepare pEV^spike^, recombinant spike protein (4 µg; MyBioSource, MBS434283) was added to 2.5 × 10⁶ platelets and incubated for 4 h. Supernatants were collected, and EVs were isolated and quantified as described above.

### 4.8 Quantitative PCR for EV‐Carried tsRNAs

Total tsRNAs were extracted from EVs using the rtStar tRF&tiRNA Pretreatment Kit (Arraystar, AS‐FS‐005) to remove interfering RNA modifications. Synthetic Caenorhabditis elegans miRNA (cel‐miR‐39; 25 fmol; Thermo Fisher Scientific, MC10956) was spiked in as an internal control. For cDNA synthesis, 250 ng of total tsRNA was reverse transcribed using the rtStar cDNA Synthesis Kit (Arraystar, AS‐FS‐003‐02). Quantitative real‐time PCR (qRT‐PCR) was performed using the LightCycler 480 SYBR Green I Master Mix (Roche, 04707516001) and specific primers (Table S1) on a LightCycler 96 system (Roche), following the manufacturer's instructions. Relative expression levels were calculated using the 2–^ΔCt^ method (ΔCt = Ct_target_—Ct_cel‐miR‐39_), and fold changes were derived using the ΔΔCt method for comparisons between patient and control samples.

### 4.9 Loading of tsRNA Mimic or Control into EVs

A total of 0.1 µmol of tRF‐His‐GTG‐1 mimic or control RNA [58] was mixed with 20 µL of purified pEVs (∼5 × 10⁸ particles) and electroporated using a Neon Transfection System (Thermo Fisher Scientific, MPK1025) at 500 V, 10 ms, 3 pulses. Following electroporation, samples were treated with RNase A (1 U; QIAGEN, 19101) for 30 min at room temperature, followed by incubation with RNase inhibitor (Thermo Fisher Scientific, N8080119) for 5 min. Loaded EVs were reisolated by ultracentrifugation as previously described [24].

### 4.10 RNA‐seq Analysis

Total RNA (1 µg) from neutrophils was used for library preparation with the TruSeq Stranded mRNA Library Prep Kit (Illumina, RS‐122‐2001/2002) according to the manufacturer's protocol. mRNA was enriched using oligo(dT) beads, fragmented, and reverse‐transcribed into cDNA. After adaptor ligation and PCR amplification, libraries were purified with the AMPure XP system (Beckman Coulter, USA), quality‐checked with the Qsep400 System (Bioptic Inc., Taiwan), and quantified using a Qubit 2.0 Fluorometer (Thermo Fisher Scientific, USA). Paired‐end sequencing (150 bp) was performed on an Illumina NovaSeq platform.

### 4.11 Transient Transfection

Human primary neutrophils (2 × 10⁶ cells per reaction) were transiently transfected with 500 ng DNA of the RAB7A expression plasmid (OriGene, RC201776) or an empty control vector using the Neon Transfection System Kit (Thermo Fisher Scientific, MPK1025) according to the manufacturer's instructions. Cells were then incubated at 37°C in RPMI‐1640 medium supplemented with 10% heat‐inactivated fetal bovine serum for 24 h. Transfection efficiency was confirmed by immunoblotting for RAB7A, showing approximately 40%–50% overexpression relative to the control. Cell viability after transfection remained above 85%, as determined using the CellTiter‐Glo Luminescent Cell Viability Assay (Promega, G7570).

### 4.12 Reporter Assay

To investigate whether tRF‐His‐GTG‐1 directly targets the 3′UTR of RAB7A, a dual‐luciferase reporter assay was performed. The wild‐type or mutant RAB7A 3′UTR containing the predicted tRF‐His‐GTG‐1–binding site was cloned downstream of the firefly luciferase gene in the pMIR‐REPORT Luciferase vector (Thermo Fisher Scientific, AM5795). The MT construct was generated by substituting the predicted tRF‐His‐GTG‐1 seed‐binding sequence (5′‐ACAGGCUCACACCCAGUCUGAUCAGG‐3′) with a scrambled sequence (5′‐ACUCGCUCUCAGCGAGACUCUAGUCCG‐3′), thereby disrupting complementarity with tRF‐His‐GTG‐1. HEK293T cells (1.5 × 10⁵ cells) were transfected with 200 ng of the reporter construct and 50 nm of either tRF‐His‐GTG‐1 mimic or negative control mimic using Lipofectamine RNAiMAX (Thermo Fisher Scientific, 13778150), according to the manufacturer's instructions. A *Renilla* luciferase vector (10 ng) was cotransfected as an internal control for normalization. After 48 h, cells were lysed and luciferase activities were measured using the Dual‐Luciferase Reporter Assay System (Promega, E1910). Firefly luciferase activity was normalized to *Renilla* luciferase activity, and results were expressed as relative luciferase activity compared to the control group.

### 4.13 Immunofluorescence Assay

Neutrophils were fixed in 4% paraformaldehyde (Sigma‐Aldrich, 47608) for 10 min at room temperature, washed in PBS (Thermo Fisher Scientific, 70011044), permeabilized in PBS containing 1% bovine serum albumin (BSA, Thermo Fisher Scientific, 15561020) and 0.2% saponin (Sigma‐Aldrich, 47036), and blocked in PBS containing 2% BSA for 1 h. Lysosomes were stained overnight at 4°C with anti‐LAMP1 antibody (1:200, Abcam, 25630), and lipid droplets were labeled with 2 µm BODIPY 493/503 (Thermo Fisher Scientific, D3922)for 30 min. Coverslips were mounted using SlowFade mounting medium (Thermo Fisher Scientific, S36967), and images were acquired using an Olympus FV1000 confocal microscope. Image analysis was performed using FV10‐ASW software version 4.2 (Olympus).

### 4.14 Immunohistochemistry

Formalin‐fixed, paraffin‐embedded lung tissue sections were incubated with either anti–SARS‐CoV‐2 spike antibody (Cell Signaling Technology, #99423) or antineutrophil elastase (NE) antibody (Santa Cruz Biotechnology, sc‐53388). Ten randomly selected fields per animal were analyzed at 100× or 200× magnification using an Olympus FV1000 confocal microscope and FV10‐ASW software version 4.2 (Olympus).

### 4.15 Immunoblotting

Cells or EVs were lysed in RIPA lysis and extraction buffer (Thermo Fisher Scientific, 89900) supplemented with protease inhibitors (Roche, 04693159001). Forty micrograms of total protein was separated by SDS–polyacrylamide gel electrophoresis (SDS‐PAGE), transferred to PVDF membranes (Millipore, IPVH00010), and probed with primary and HRP‐conjugated secondary antibodies (Supporting Information). Signals were detected using enhanced chemiluminescence (ECL; Millipore, WBULS0100) and visualized on a CCD imaging system (GE Healthcare, USA). Band intensities were quantified using ImageJ software, and β‐actin (Santa Cruz Biotechnology, sc‐47778) served as the loading control. All experiments were performed in triplicate. Data are presented as mean ± standard deviation (SD). Statistical comparisons were performed using a two‐tailed unpaired Student's *t*‐test (GraphPad Prism, version 8). Densitometric quantification results are provided in Supporting Information S2.

### 4.16 Flow Cytometry

EVs were analyzed using the Exo‐Flow Exosome Capture Kit (System Biosciences, CSFLOWBASICA‐1) according to the manufacturer's instructions. Briefly, purified EVs were incubated overnight at 4°C with magnetic beads coated with antihuman CD63 monoclonal antibodies (BioLegend, 303005). Beads were then washed and stained with Exo‐FITC for general EV detection and PE‐conjugated anti‐CD41 (BioLegend, 303706) for platelet‐specific EV identification. PE‐conjugated IgG1 (BioLegend, 400111) served as an isotype control. Samples were analyzed using a FACSCanto II flow cytometer (BD Biosciences, USA), and data were processed with CellQuest (version 6.0, BD Biosciences) or FlowJo (version 10.10.0, BD Biosciences) software. Flow cytometry data were analyzed using a standard doublet discrimination strategy. Side scatter width (SSC‐W) versus side scatter height (SSC‐H) and forward scatter width (FSC‐W) versus forward scatter height (FSC‐H) plots were used to exclude doublets. The main EV population was gated based on SSC‐A versus FSC‐A to eliminate debris and non‐EV events.

### 4.17 Quantification of Cytosolic ROS Production

Cytosolic ROS levels were measured using the fluorescent dye dihydrorhodamine 123 (DHR123; Thermo Fisher Scientific, D23806). Neutrophils were incubated with DHR123 according to the manufacturer's instructions, and fluorescence intensity was quantified by flow cytometry. Data were analyzed using CellQuest software (version 6.0, BD Biosciences) and expressed as mean fluorescence intensity (MFI).

### 4.18 Statistics

Data are presented as mean ± standard deviation (SD). Data normality was assessed using the Shapiro–Wilk test. Parametric tests (unpaired two‐tailed Student's *t*‐test for two‐group comparisons or one‐way ANOVA with Bonferroni's post hoc test for multiple‐group analyses) were applied to normally distributed data, whereas nonparametric tests (Mann–Whitney *U* or Kruskal–Wallis test with Dunn's post hoc correction) were used otherwise. Correlations were evaluated using Spearman's rank correlation coefficient. A *p*‐value < 0.05 was considered statistically significant. All statistical analyses were performed using GraphPad Prism software (version 8.0; GraphPad Software, USA).

## Author Contributions

All authors made substantive intellectual contributions to the present study and approved the final manuscript. T.L.L. conceived of the study and generated the original hypothesis. T.L.L. and P.Y.L. designed the study. P.Y.L., Y.M.C., K.T.T., and D.Y.C. acquired the clinical data. T.L.L. and H.J.L. performed research. T.L.L. and P.Y.L. analyzed data. T.L.L., P.Y.L., Y.M.C., K.T.T., H.J.L., and D.Y.C. drafted and revised the manuscript.

## Funding

This work was supported by grants from National Science and Technology Council (No. NSTC 112‐2314‐B‐075A‐002‐MY3), and Taichung Veterans General Hospital (Nos. TCVGH‐1137305C, TCVGH‐1133902E, and TCVGH‐1143918C).

## Conflicts of Interest

The authors declare no conflict of interest.

## Disclosure Statement

No potential conflict of interest was reported by the author(s).

## Supporting information




**Supporting File 1**: advs73502‐sup‐0001‐SuppMat.pdf.

**Supporting File 2**: advs73502‐sup‐0002‐Additional file 2_Densitometric analysis of immunoblot.pdf.

**Supporting File 3**: advs73502‐sup‐0003‐Additional file 3_Unedited blot and gel images.pdf.

## Data Availability

The data that support the findings of this study are available in the Supporting Information of this article.
