# Supplementary material for: Extracellular Vesicle‐Delivered tRF‐His‐GTG‐1 Reprograms Neutrophil Lipophagy and Triggers Inflammation in COVID‐19
Source: Adv Sci (Weinh). 2026 Jan 14;13(13):e08695. doi: 10.1002/advs.202508695 (PMC12955898; doi:10.1002/advs.202508695)
Supplement: Supplementary file 1 — Supporting File 1: advs73502‐sup‐0001‐SuppMat.pdf. [file ADVS-13-e08695-s002.pdf]

Supplementary information

Extracellular vesicle–delivered tRF-His-GTG-1 reprograms neutrophil lipophagy and triggers inflammation in COVID-19

Tsai-Ling Liao[1-3*], Po-Yu Liu[4*], Yi-Ming Chen[1-3,5,6], Kuo-Tung Tang[6], Hung-Jen Liu[2,3,7,8], Der-Yuan Chen[9,10]

[1]Department of Medical Research, Taichung Veterans General Hospital, Taichung 407, Taiwan

[2]Rong Hsing Research Center for Translational Medicine, National Chung Hsing University, Taichung 402, Taiwan

[3]Ph.D. Program in Translational Medicine, National Chung Hsing University, Taichung 402, Taiwan

[4]Division of Infection, Taichung Veterans General Hospital, Taichung 407, Taiwan

[5]Department of Post-Baccalaureate Medicine, College of Medicine, National Chung Hsing University, Taichung 402, Taiwan

[6]Division of Allergy, Immunology and Rheumatology, Department of Internal Medicine, Taichung Veterans General Hospital, Taichung 407, Taiwan

[7]Institute of Molecular Biology, National Chung Hsing University, Taichung 402, Taiwan

[8]The iEGG and Animal Biotechnology Center, National Chung Hsing University, Taichung 402, Taiwan

[9]Translational Medicine Laboratory, Rheumatology and Immunology Center, China Medical University Hospital, Taichung 404, Taiwan

[10]College of Medicine, China Medical University, Taichung 404, Taiwan

Corresponding author and address reprint requests:

Dr. Tsai-Ling Liao, Department of Medical Research, Taichung Veterans General Hospital, Taichung, Taiwan

Address: No.1650, Sec.4, Taiwan Boulevard, Xitun Dist., Taichung City 40705, Taiwan

Tel.: 886-4-23592525, extension 4020; Fax: 886-4-23592705

Email: tlliao@vghtc.gov.tw

***Co-corresponding author:**

Dr. Po-Yu Liu, Division of Infection, Department of Internal Medicine, Taichung Veterans General Hospital, Taichung, Taiwan

Address: No.1650, Sec.4, Taiwan Boulevard, Xitun Dist., Taichung City 40705, Taiwan

Tel.: 886-2-23592525; Fax: 886-2-23595046

Email: pyliu@vghtc.gov.tw

Supplementary Materials

Reagent or Resource	Source	Identifier (Cat No.)
**Cell line**		
HEK-Blue hTLR8 cell	InvivoGen	hkb-htlr8
THP1-Difluo hLC3 Cells	InvivoGen	thpdf-hlc3
**Antibodies**		
Mouse anti-Alix antibody	Santa Cruz Biotechnology	sc-53540
Mouse anti-β-actin antibody	Santa Cruz Biotechnology	sc-47778
Mouse anti-CD9 antibody	Abcam	ab58989
Mouse anti-CD63 antibody	Abcam	ab68418
Mouse anti-CD81 antibody	Abcam	ab79559
Mouse anti-citH3 antibodies	Abcam	ab5103
Mouse anti-GAPDH antibody	Santa Cruz Biotechnology	sc-32233
Mouse anti-IL-1β antibody	Santa Cruz Biotechnology	sc-52012
Mouse anti-IL-8 antibody	Santa Cruz Biotechnology	sc-8427
Mouse anti-LAMP-1 antibody	Abcam	ab25630
Mouse anti-MPO/Myeloperoxidase antibody (WB)	Santa Cruz Biotechnology	sc-52707
Mouse anti-MPO/Myeloperoxidase antibody (ELISA)	Bio-Rad	MCA1757
Mouse anti-neutrophil elastase antibody	Santa Cruz Biotechnology	sc-53388
Mouse anti-p47^{phox} antibody	Santa Cruz Biotechnology	sc-17845
Mouse anti-PLIN2 antibody	Santa Cruz Biotechnology	sc-377429
Mouse anti-PLIN3 antibody	Abcam	ab47639

Rabbit anti-Calnexin antibody	Cell Signaling Technology	#2679
Rabbit anti-integrin αIIb/CD41 antibody	Cell Signaling Technology	#13807
Rabbit anti-LC3B antibody	Cell Signaling Technology	#2775
Rabbit anti-mTOR antibody	Cell Signaling Technology	#2972
Rabbit anti-Phospho-mTOR (Ser2448) antibody	Cell Signaling Technology	#2971
Rabbit anti-Rab7A antibody	Cell Signaling Technology	#9367
Rabbit anti-SARS-CoV-2 spike protein antibody	Cell Signaling Technology	#99423
Rabbit anti-SQSTM1/p62 antibody	Cell Signaling Technology	#8025
Rabbit anti-VAMP8 antibody	Cell Signaling Technology	#13060
Mouse anti-TLR7 antibody	Santa Cruz Biotechnology	sc-57463
Mouse anti-TLR8 antibody	Santa Cruz Biotechnology	sc-373760
Mouse anti-TLR9 antibody	Santa Cruz Biotechnology	sc-515921
Anti-mouse IgG, HRP-linked antibody	Cell Signaling Technology	#7076
Anti-rabbit IgG, HRP-linked antibody	Cell Signaling Technology	#7074
**Chemicals and Assay kits**		
Apyrase	Sigma-Aldrich	A6535
BODIPY 493/503	Thermo Fisher Scientific	D3922
Bovine Serum Albumin (fatty acid free)	Sigma-Aldrich	A8806
cel-miR-39-3p mimic	Thermo Fisher Scientific	MC10956
Cytochalasin D	Sigma-Aldrich	C8273
CU-CPT9a	InvivoGen	inh-cc9a

Dihydrorhodamine 123	Thermo Fisher Scientific	D23806
Ficoll®-Paque Premium	GE Healthcare Biosciences	GE17-5442-02
Hank's balanced salt solution	Sigma-Aldrich	55021C
Hoechst 33342	Thermo Fisher Scientific	H3570
imiquimod(R837)	Invivogen	tlrl-imqs
LightCycler 480 SYBR Green I Master	Roche	04707516001
Percoll	Sigma-Aldrich	P1644
PKH67 Green Fluorescent Cell Linker Mini Kit	Sigma-Aldrich	MINI67
Polymorphprep	Axis-Shield	1895
Prostaglandin I2	Sigma-Aldrich	P6188
QIAamp DNA Blood Mini Kit	QIAGEN	51106
Recombinant COVID 19 Spike Protein	MyBioSource	MBS434283
Resiquimod(R848)	Invivogen	tlrl-r848
rtStar tRF&tiRNA Pretreatment Kit	Arraystar	AS-FS-005
rtStar First-Strand cDNA Synthesis Kit	Arraystar	AS-FS-003-02
Tyrode's buffer	Sigma-Aldrich	T2397
Trizol	Thermo Fisher Scientific	15596018
On TARGETplus SMARTpool siTLR7	Dharmacon	L-004714-00-0005
On TARGETplus SMARTpool siTLR8	Dharmacon	L-004715-00-0005
Human IL-1 beta/IL-1F2 Quantikine ELISA Kit	R&D	DLB50
Human IL-8 Quantikine ELISA Kit	R&D	D8000C
ExoELISA-ULTRA Complete Kit	System Biosciences	EXEL-ULTRA-CD63-1

Table S1. The sequence of tsRNAs primer sets, mimic, and inhibitor used in this study.

Name	Sequence	Ref.
tRF Universal Primer R	5′-AGTGCAGGGTCCGAGGTATT-3′	[24]
tRF-His-GTG-1_F	5′-CGCGGCCGGACGAAGG-3′	[24]
tRF-chrM.Pro-TGG _F	5′-CGCGCGCGCGAAGACC-3′	[24]
tRF-Val-AAC-1-M7_F	5′-CGCGCGCGCGGCCGAG-3′	[24]
tRF-Ala-TGC	5′-CGCGCGAAGGGCAGCA-3′	[24]
tRF-Gly-GCC	5′-CGCGCGCGCGCCGCCA-3′	[24]
tRF-His-GTG-1 mimic	5′-GCCGUGAUCGUAUAGUGGUUAGUACUCU-3′ (2'-O-Methyl modification)	[24]
tRF-His-GTG-1 inhibitor	5′-AACCACUAUACGAUCACGGC-3′ (2'-O-Methyl modification)	[24]
tRF mimic negative control	5′-UUGUACUACACAAAAGUACUG-3′ (2'-O-Methyl modification)	[58]
tRF inhibitor negative control	5′-CAGUACUUUUGUGUAGUACAA-3′ (2'-O-Methyl modification)	[58]

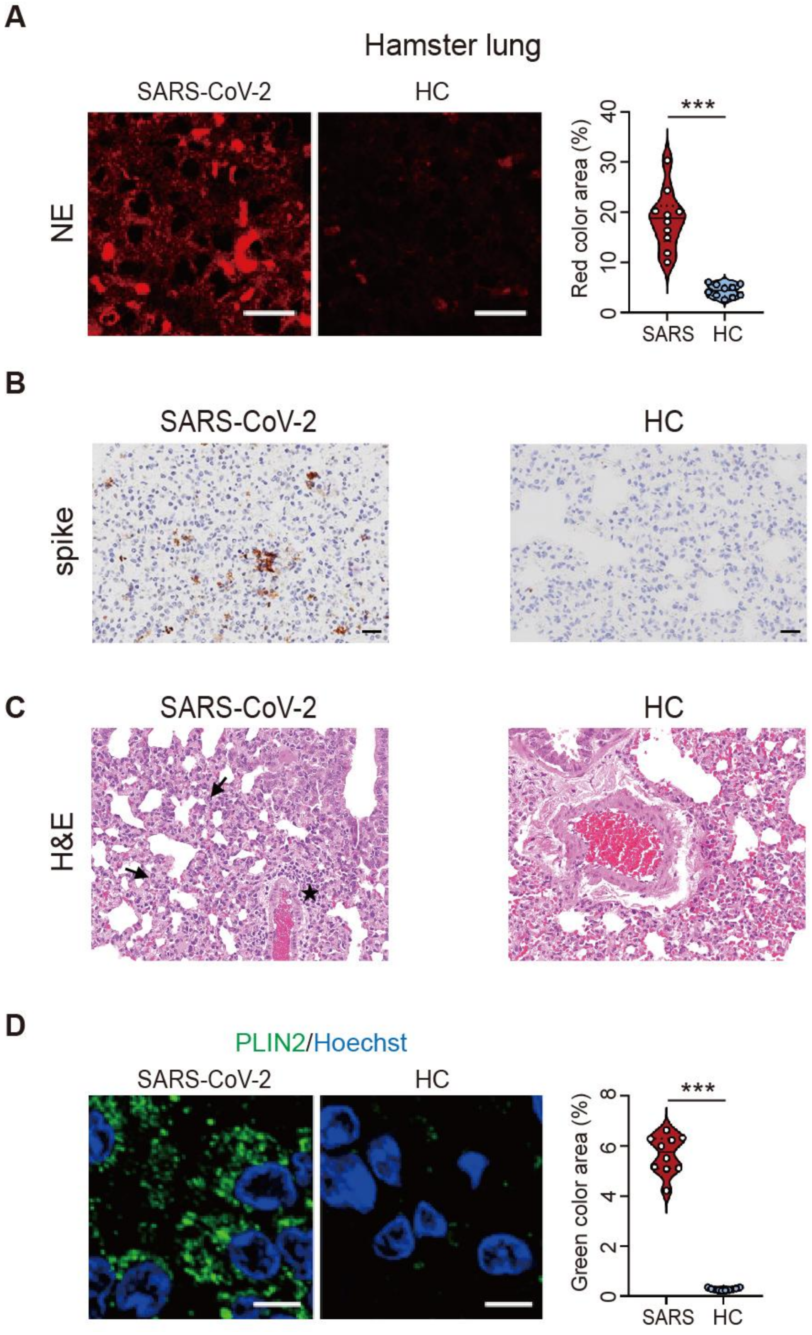

Figure S1. Lung pathology in hamsters 14 days after SARS-CoV-2 infection. Representative images of (A) neutrophil elastase (NE) immunostaining, (B) SARS-CoV-2 spike protein immunostaining, (C) hematoxylin and eosin (H&E) staining, and (D) perilipin 2 (PLIN2) immunostaining in lung sections from SARS-CoV-2-infected and healthy control (HC) hamsters. Quantification of NE staining (red color area) and PLIN2 staining (green color area) is shown at right. In H&E staining, the black asterisk indicates perivascular mixed-cellular inflammation, and black arrows highlight hyperplasia of type II alveolar epithelial cells. Scale bars: 5 μm (immunofluorescence), 20 μm (Immunohistochemistry). Statistical significance was assessed using the Mann–Whitney *U* test (two-tailed) for two-group comparisons. ***$P < 0.005$.

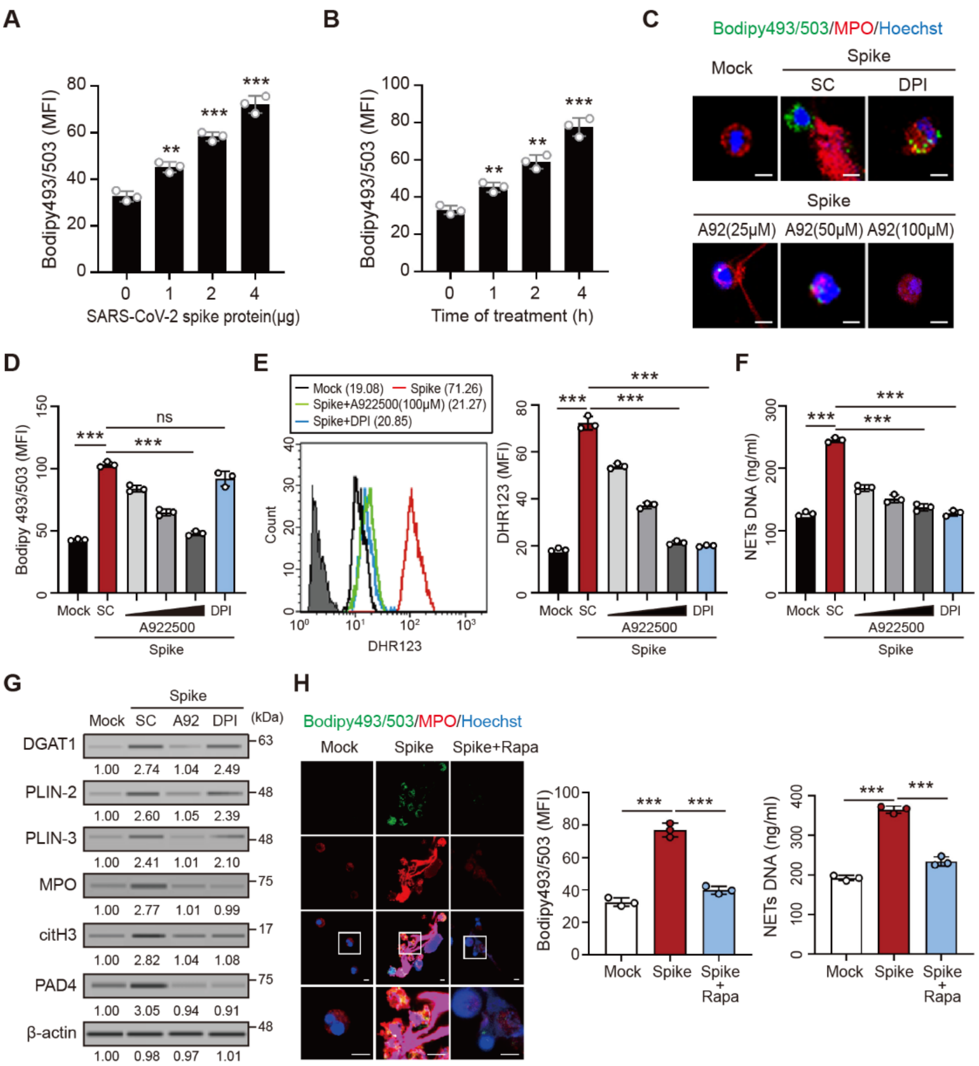

Figure S2. Recombinant SARS-CoV-2 spike protein induces lipid droplet (LD) accumulation in neutrophils. Human neutrophils were treated with spike protein at varying (A) concentrations and (B) time points. LD levels were analyzed by flow cytometry. (C) Immunofluorescence imaging showing that spike protein treatment induced intracellular LD accumulation (green, Bodipy493/503) and neutrophil extracellular trap (NET) formation (red, MPO), both of which were suppressed by the DGAT1 inhibitor A922500 or the ROS inhibitor diphenyleneiodonium (DPI). (D–F) Quantitative analysis of (D) LD accumulation, (E) ROS production, and (F) NET DNA release in spike-stimulated neutrophils treated with increasing concentrations of A922500 (25–100 μM) or DPI. (G) Immunoblot analysis showing increased expression of PLIN-2, PLIN-3, MPO, and citH3 in spike-treated neutrophils, which was attenuated by A922500 or DPI treatment. (H) Immunofluorescence imaging showing that spike protein induced intracellular LD accumulation (green, Bodipy493/503) and NET formation (red, MPO), both of which were suppressed by rapamycin treatment. Scale bars, 5 μm. All experiments were performed in triplicate, and data are presented as mean ± SD. Statistical significance was assessed using the Kruskal–Wallis test with Dunn's post hoc correction for multiple groups, or the Mann–Whitney *U* test or unpaired two-tailed Student's *t*-test for two-group comparisons, as described in the Methods. **$P < 0.01$, ***$P < 0.005$. Abbreviations: A92, A922500; Rapa, rapamycin.

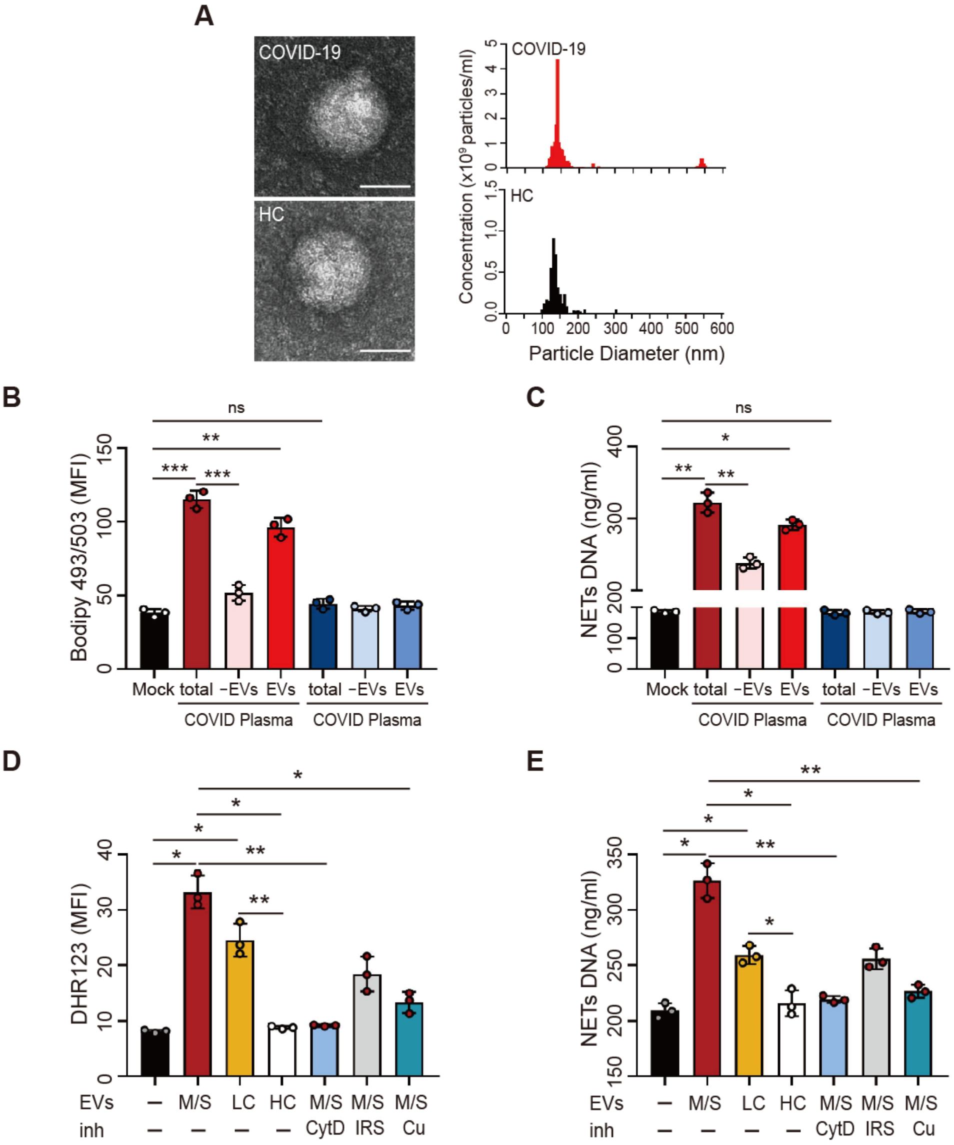

Figure S3. Characterization and functional effects of plasma-derived extracellular vesicles (EVs) from COVID-19 patients. (A) Negative-staining TEM (left) and nanoparticle tracking analysis (right) showing typical EV morphology and increased particle numbers in COVID-19 plasma compared with healthy controls (HC). Scale bars, 100 nm. (B, C) Quantification of (B) lipid droplet (LD) accumulation and (C) NET DNA release in neutrophils treated with plasma, EV-depleted plasma, or isolated EVs from COVID-19 patients or HC. (D, E) COVID-19 patient–derived EVs enhanced (D) reactive oxygen species (ROS) production and (E) NET DNA release in neutrophils, which were reduced by cytochalasin D (Cyt D), the TLR7 inhibitor IRS661 (IRS), or the TLR8 inhibitor Cu-CPT9a (Cu). All experiments were performed in triplicate, and data are presented as mean ± SD. Statistical significance was assessed using the Kruskal– Wallis test with Dunn's post hoc correction for multiple groups, or the Mann–Whitney U test or unpaired two-tailed Student's t-test for two-group comparisons, as described in the Methods. *P < 0.05, **P < 0.01, ***P < 0.005; ns, not significant. Abbreviations: M/SC, moderate/severe COVID-19; LC, long COVID; RC, recovered COVID-19.

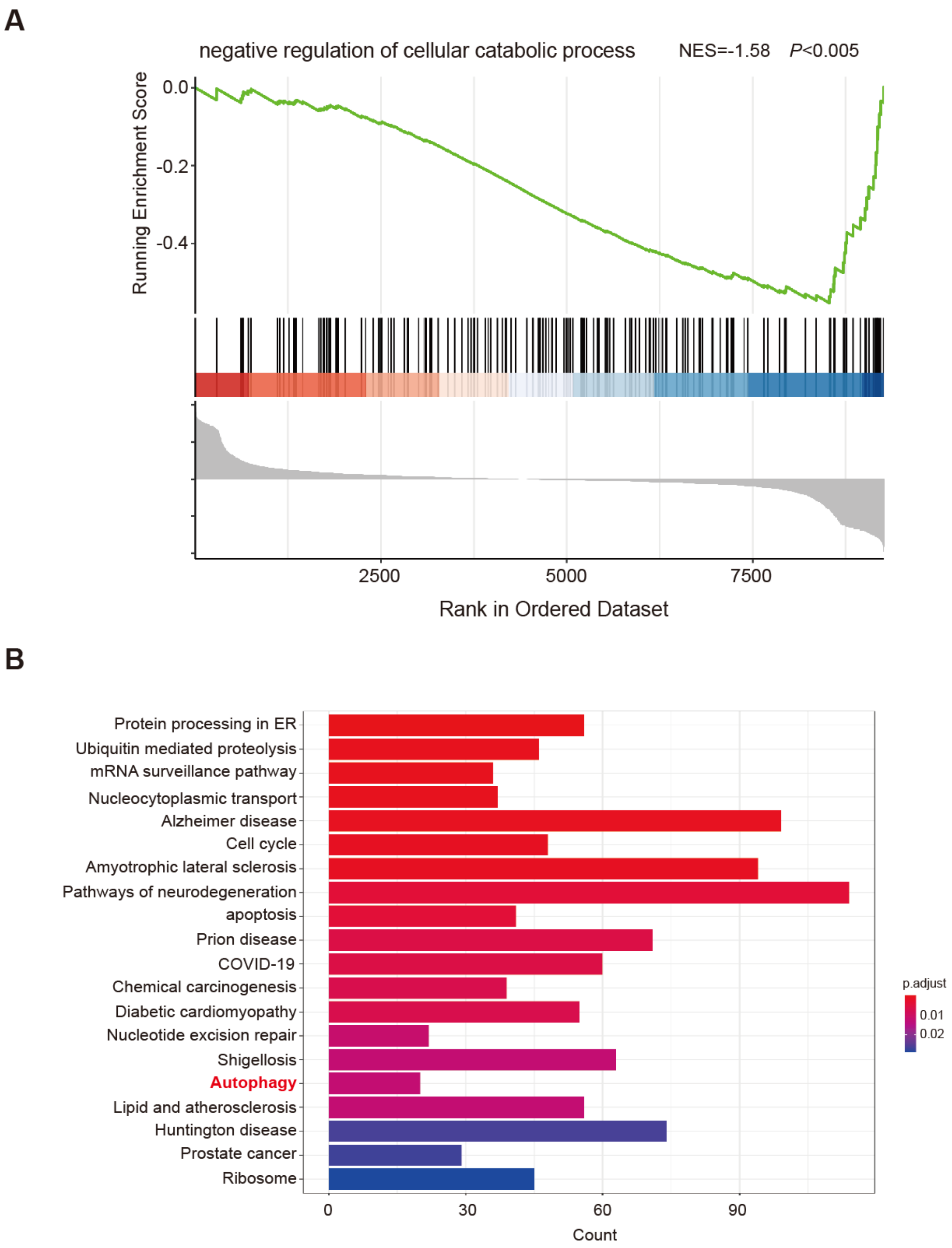

Figure S4. Spike protein treatment impairs catabolic and autophagy pathways in neutrophils. (A) GSEA enrichment plot showing altered regulation of the "negative regulation of cellular catabolic process" gene set. (B) KEGG pathway enrichment analysis showing dysregulation of autophagy-related pathways.

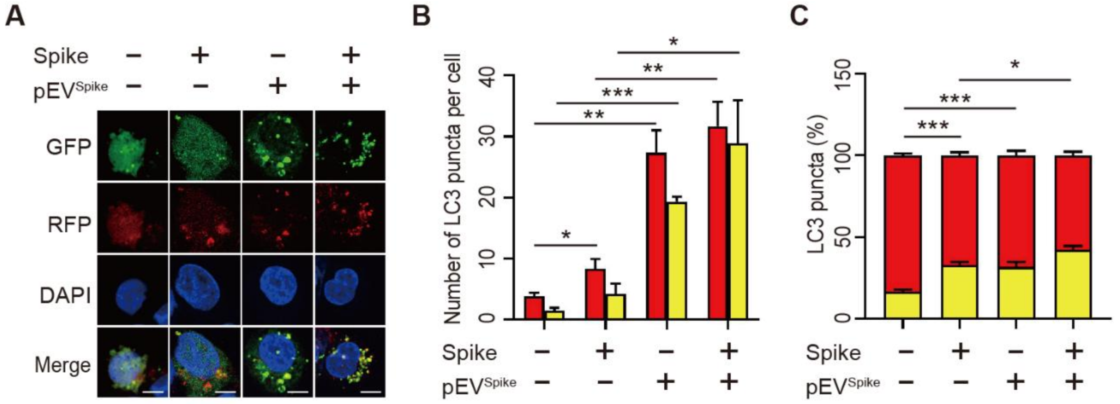

Figure S5. THP-1 cells stably expressing the RFP-GFP-LC3 fusion protein were treated with the indicated reagent for 24 h and observed via (A) confocal microscopy. (B) The numbers of RFP^+GFP^- LC3 (red) and RFP^+GFP^+LC3 (yellow) puncta per cell in individual treatments were quantified. (C) The percentage of total of yellow puncta (autophagosomes) and red puncta (autolysosomes) per cell in individual treatments. All of the experiments were performed in triplicate, and the data are presented as the mean ± SD. The scale bar in the IFA image represents 10 μm. Statistical significance was determined using a two-tailed unpaired Student's *t*-test. $^*P < 0.05$, $^{**}P < 0.01$, $^{***}P < 0.005$.

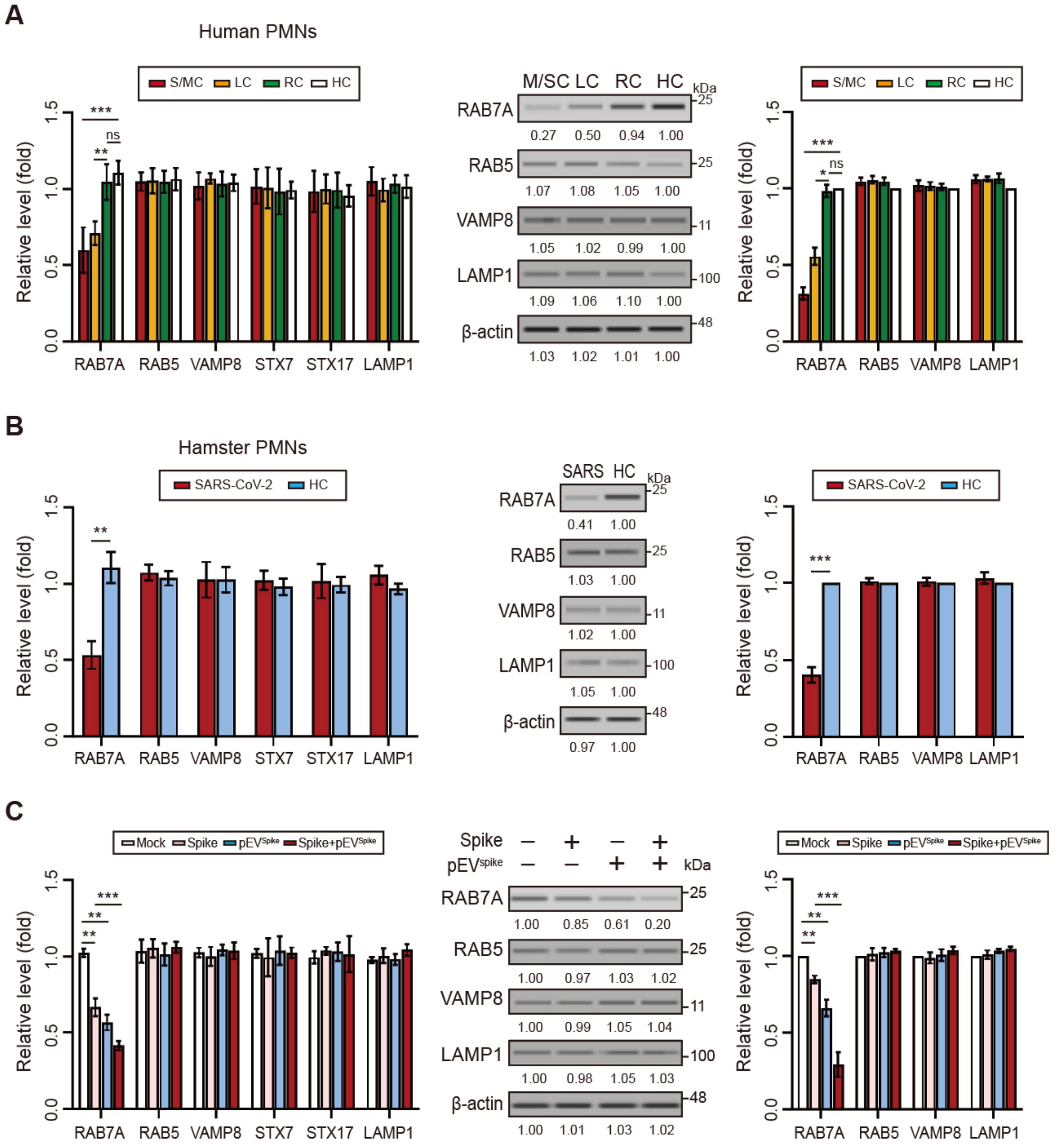

Figure S6. Validation of RAB7A specificity among autophagy- and lysosome-related genes. qRT-PCR (left) and immunoblot (middle and right) analyses of RAB7A, RAB5A, VAMP8, and LAMP1 were performed in (A) neutrophils from COVID-19 patients with varying disease status (M/SC, LC, RC) and healthy controls, (B) SARS-CoV-2–infected hamsters, and (C) spike protein– and pEV^{spike}-treated human neutrophils. β-actin was used as a loading control. Densitometric quantification of immunoblotting is shown in the right panel. All experiments were performed in triplicate, and data are presented as mean ± SD. Statistical significance was assessed using the Kruskal–Wallis test with Dunn's post hoc correction for multiple groups, and the Mann–Whitney *U* test or the unpaired two-tailed Student's *t*-test for two-group comparisons, as described in the Methods. $^{**}P < 0.01$, $^{***}P < 0.005$. Abbreviations: M/SC, moderate to severe COVID-19; LC, long COVID; RC, recovered COVID-19 patients; HC, healthy controls. PLD2, phospholipase D2.

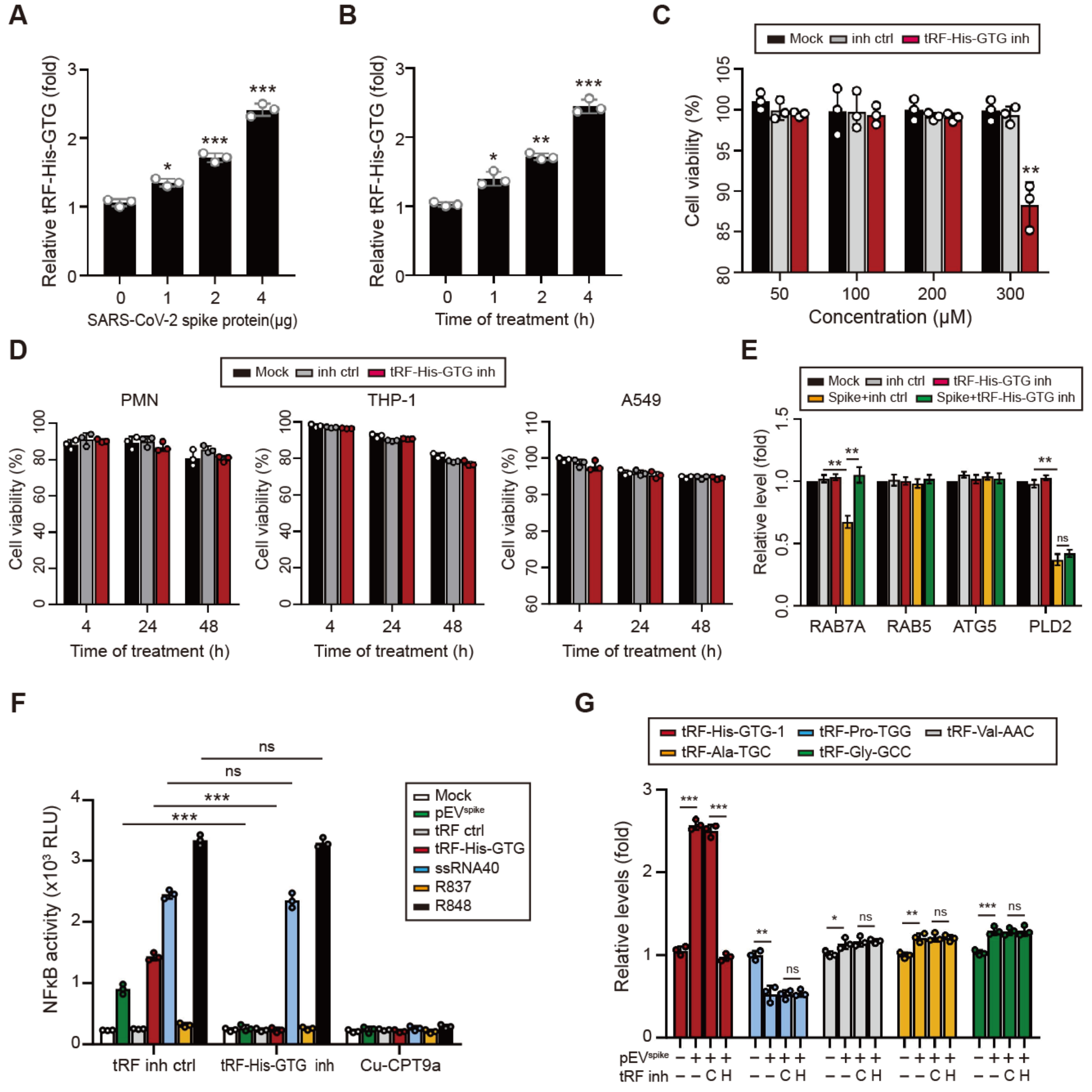

Figure S7. Recombinant SARS-CoV-2 spike protein-induced tRF-His-GTG-1 loading into platelet-derived extracellular vesicles (pEVs) and off-target assessment of the tRF-His-GTG-1 inhibitor. Human platelets were stimulated with spike protein at varying (A) concentrations and (B) time points. pEVs were isolated from the culture supernatant and analyzed for tRF-His-GTG-1 levels using qRT-PCR. (C) Cell viability of primary neutrophils (PMNs) treated for 4 h with increasing concentrations (50–300 μM) of tRF-His-GTG-1 inhibitor or scrambled control, confirming low cytotoxicity. (D) Cytotoxicity at 200 μM assessed at 4, 24, and 48 h in PMNs, THP-1 monocytes, and A549 epithelial cells. (E) qRT-PCR analysis showing selective restoration of RAB7A expression in spike-treated neutrophils by the tRF-His-GTG-1 inhibitor, with no significant changes in RAB5, ATG5, or PLD2. (F) NF-κB reporter assay in HEK-Blue hTLR8 cells stimulated with pEV^{spike}, tRF-His-GTG/control, or canonical TLR7/8 agonists in the presence of tRF-His-GTG-1 inhibitor/control, or the TLR8 antagonist Cu-CPT9a. (G) qPCR analysis of representative infection-related tsRNAs in neutrophils treated with pEV^{spike} in the presence of control inhibitor (C) or tRF-His-GTG-1 inhibitor (H). All experiments were performed in triplicate, and data are presented as mean ± SD. Statistical significance was assessed using the Kruskal–Wallis test with Dunn's post hoc correction for multiple groups, the Mann–Whitney *U* test or unpaired two-tailed Student's *t*-test for two-group comparisons, as described in the Methods. *$P < 0.05$, **$P < 0.01$, ***$P < 0.005$. ns, not significant.

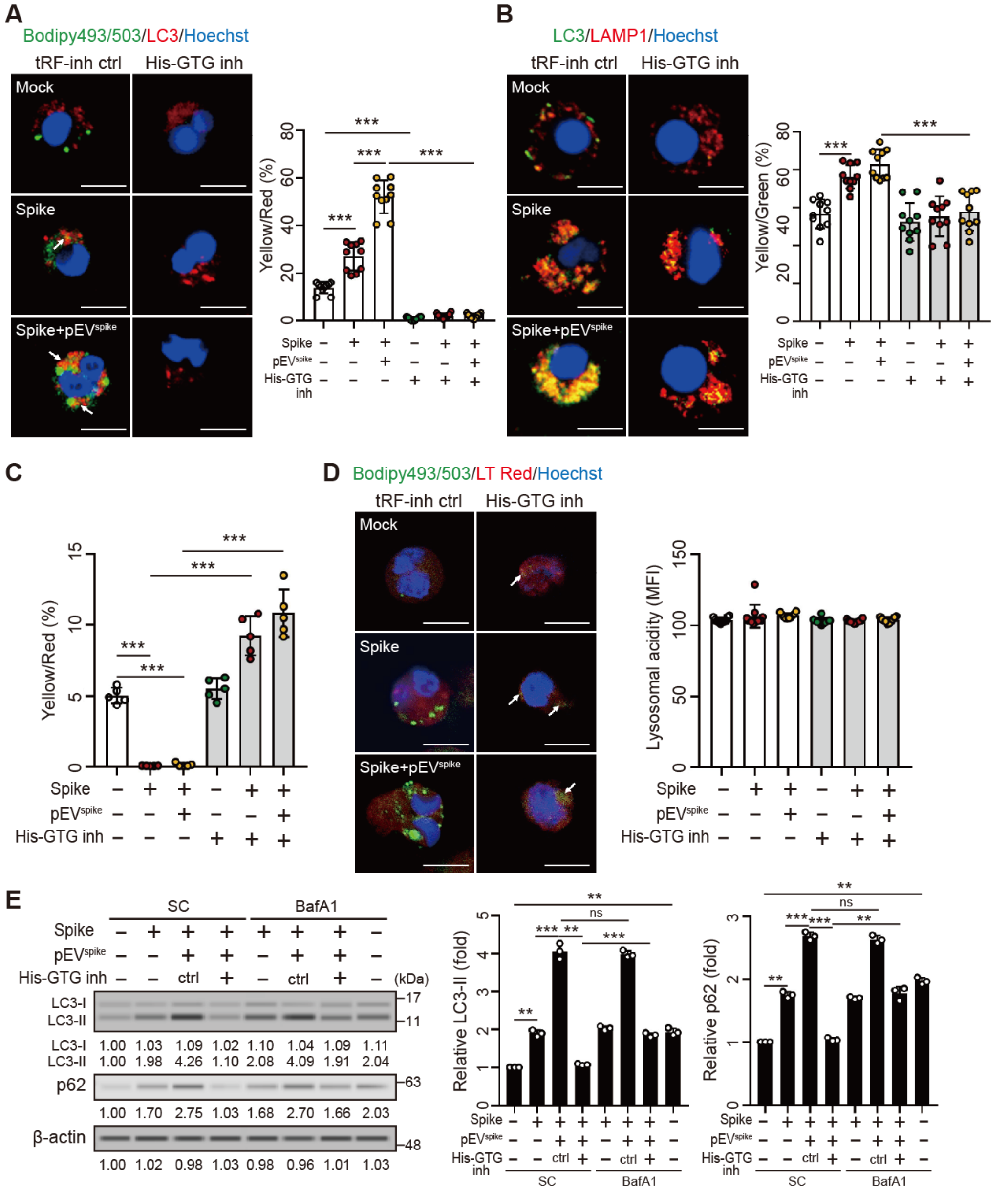

Figure S8. tRF-His-GTG-1 Inhibitor restores lipophagic flux in neutrophils. (A) Immunofluorescence analysis of LC3 and lipid droplets (LDs, BODIPY) in neutrophils treated with spike protein and/or pEV^{spike}, with or without tRF-His-GTG-1 inhibitor. Yellow/Red (%) represents the proportion of LC3 puncta colocalized with LDs. (B) Immunofluorescence analysis of LC3 and lysosomes (LAMP1) under the same conditions. Yellow/Green (%) represents the proportion of LC3 puncta colocalized with lysosomes. (C) Quantification of LD and lysosome (LAMP1) colocalization (Yellow/Red %) showing restoration of LD-lysosome fusion after tRF-His-GTG-1 inhibition. (D) Lysosomal acidity assessed by LysoTracker Red staining, showing no significant differences across groups. (E) Immunoblot analysis of LC3 and p62 in neutrophils treated with spike protein and/or pEV^{spike}, with or without tRF-His-GTG-1 inhibitor, in the presence or absence of BafA1. β-actin served as a loading control. Scale bars = 5 μm. All experiments were performed in triplicate, and data are presented as mean ± SD. Statistical significance was assessed using the Kruskal–Wallis test with Dunn's post hoc correction for multiple groups, the Mann–Whitney *U* test or unpaired two-tailed Student's *t*-test for two-group comparisons, as described in the Methods $^{*}P < 0.05$, $^{**}P < 0.01$, $^{***}P < 0.005$, ns, not significant.

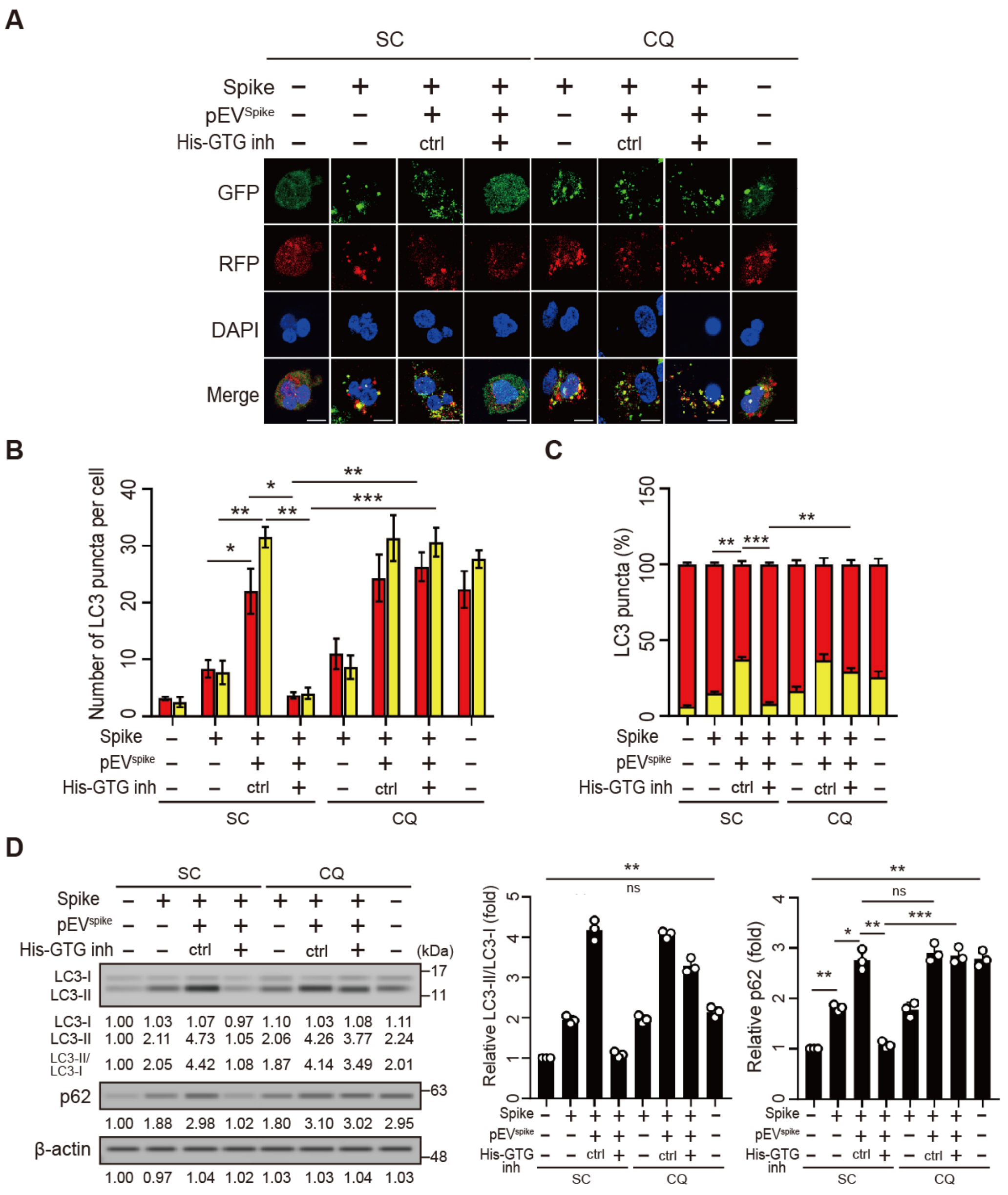

Figure S9. THP-1 cells stably expressing the RFP-GFP-LC3 fusion protein were treated with the indicated reagent for 24 h and observed via (A) confocal microscopy. (B) The numbers of RFP+GFP- LC3 (red) and RFP+GFP+LC3 (yellow) puncta per cell in individual treatments were quantified. (C) The percentage of total of yellow puncta (autophagosomes) and red puncta (autolysosomes) per cell in individual treatments. (D) Immunoblot analysis of LC3 and p62 in neutrophils treated with spike protein and/or pEV^{spike}, with or without tRF-His-GTG-1 inhibitor, in the presence or absence of chloroquine (CQ). β-actin served as a loading control. All experiments were performed in triplicate, and data are presented as mean ± SD. Statistical significance was assessed using the Kruskal–Wallis test with Dunn's post hoc correction for multiple groups, and the Mann–Whitney *U* test or unpaired two-tailed Student's *t*-test for two-group comparisons, as described in the Methods. *$P < 0.05$, **$P < 0.01$, ***$P < 0.005$, ns, not significant.

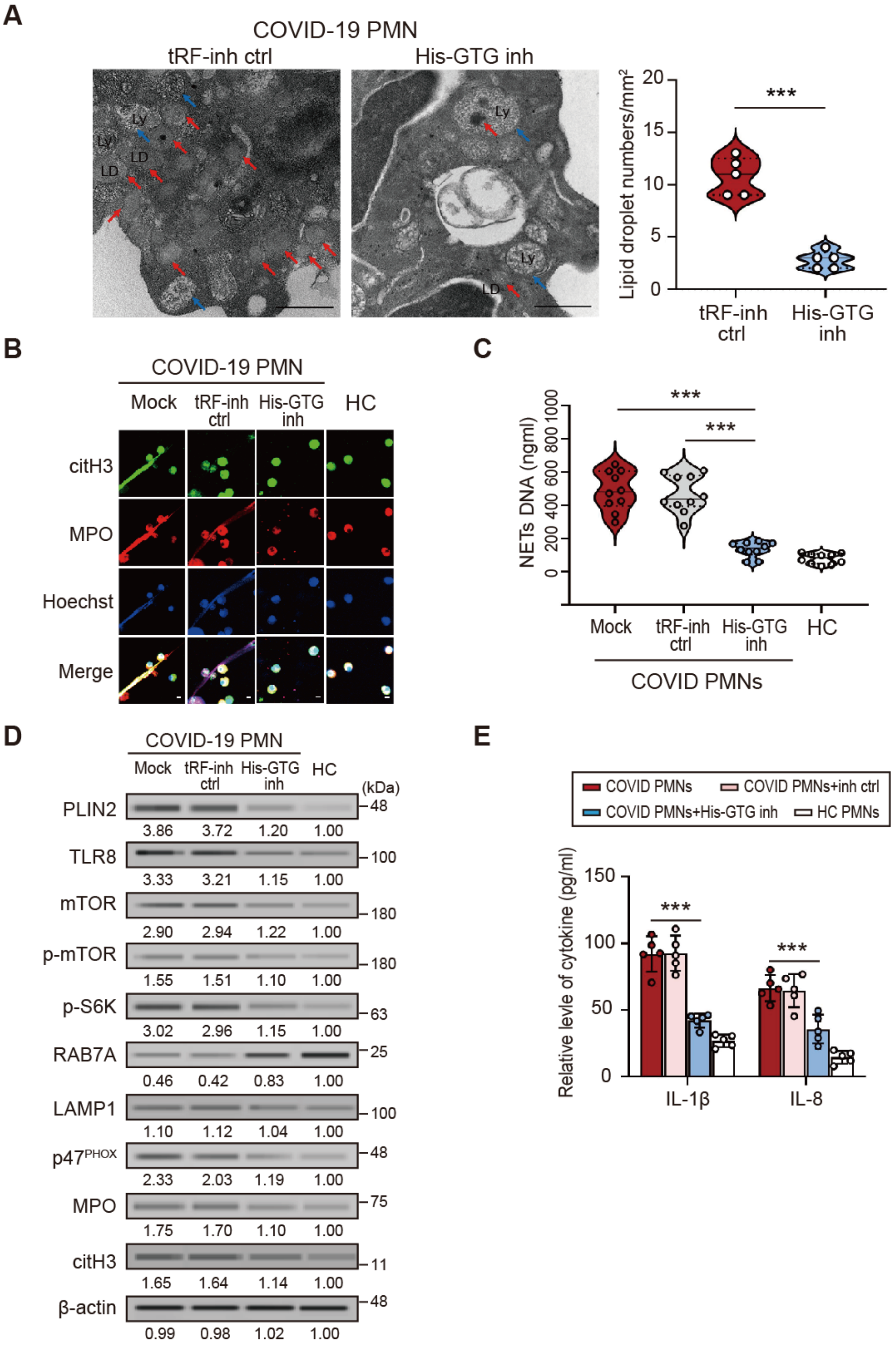

Figure S10. *Ex vivo* treatment with the tRF-His-GTG-1 inhibitor restores lipophagy and reduces NET formation and inflammatory cytokine production in neutrophils from COVID-19 patients. (A) TEM images of neutrophils treated with tRF-His-GTG-1 inhibitor or control. Lipid droplets (LD, red arrows) and lysosomes (Ly, blue arrows) are indicated; LD quantification is shown on the right. (B) Immunofluorescence staining of citH3 (green), MPO (red), and Hoechst (blue) in neutrophils from COVID-19 patients and healthy controls (HC). (C) Quantification of NET DNA release by ELISA in mock- and inhibitor-treated neutrophils. (D) Immunoblotting of lipophagy- and NET-related markers in treated neutrophils; β-actin was used as a loading control. Densitometric values normalized to HC are shown below. (E) ELISA quantification of IL-1β and IL-8 in culture supernatants. All experiments were performed in triplicate, and data are presented as mean ± SD. Statistical significance was assessed using the Kruskal–Wallis test with Dunn's post hoc correction for multiple groups, the Mann–Whitney *U* test or unpaired two-tailed Student's *t*-test for two-group comparisons, as described in the Methods. Scale bars: 500 nm (TEM), 5 µm (IFA).
