# Supplementary figures and images for: Extracellular Vesicle‐Delivered tRF‐His‐GTG‐1 Reprograms Neutrophil Lipophagy and Triggers Inflammation in COVID‐19
Source: Adv Sci (Weinh). 2026 Jan 14;13(13):e08695. doi: 10.1002/advs.202508695 (PMC12955898; doi:10.1002/advs.202508695)

Fig. 1J

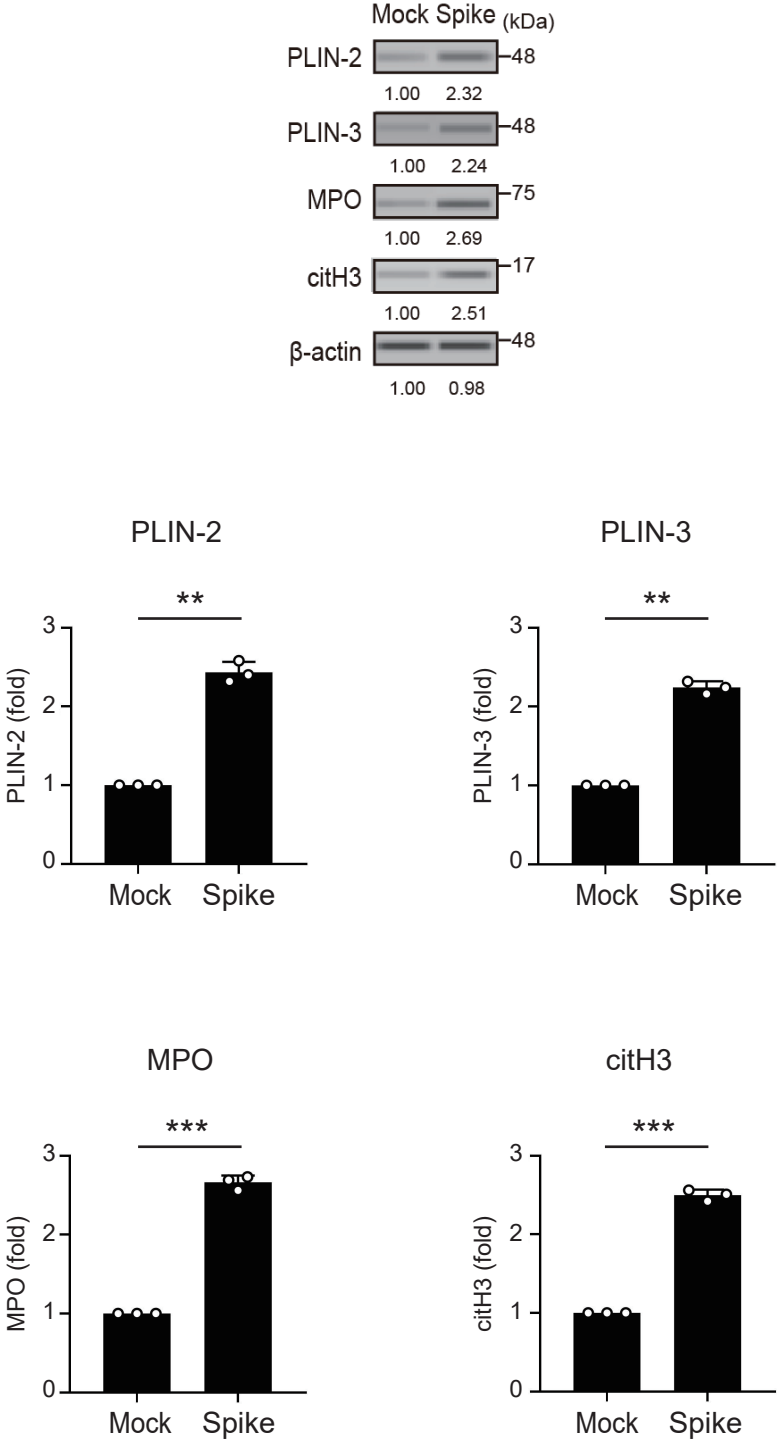

Fig. 2C

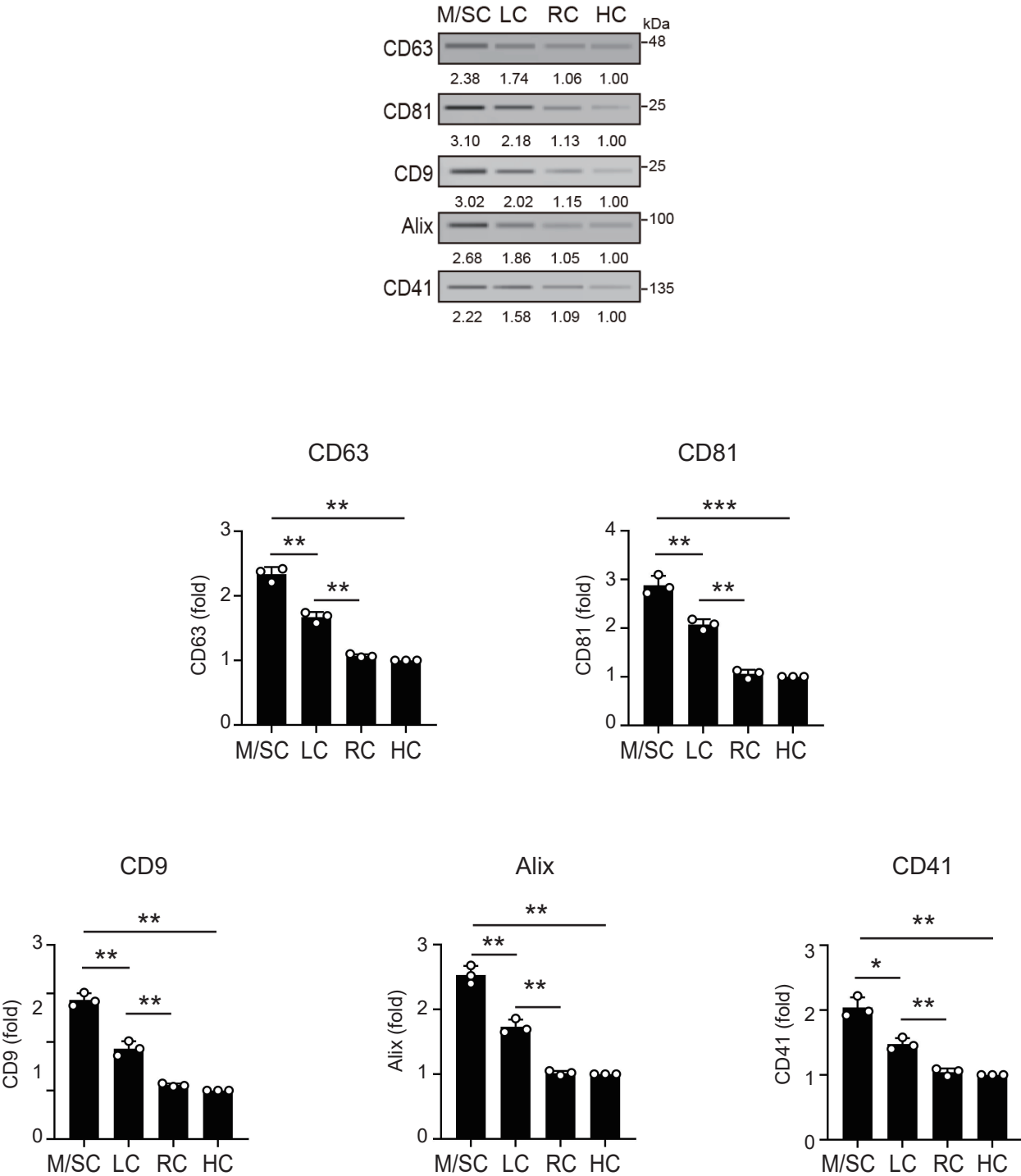

Fig. 2G

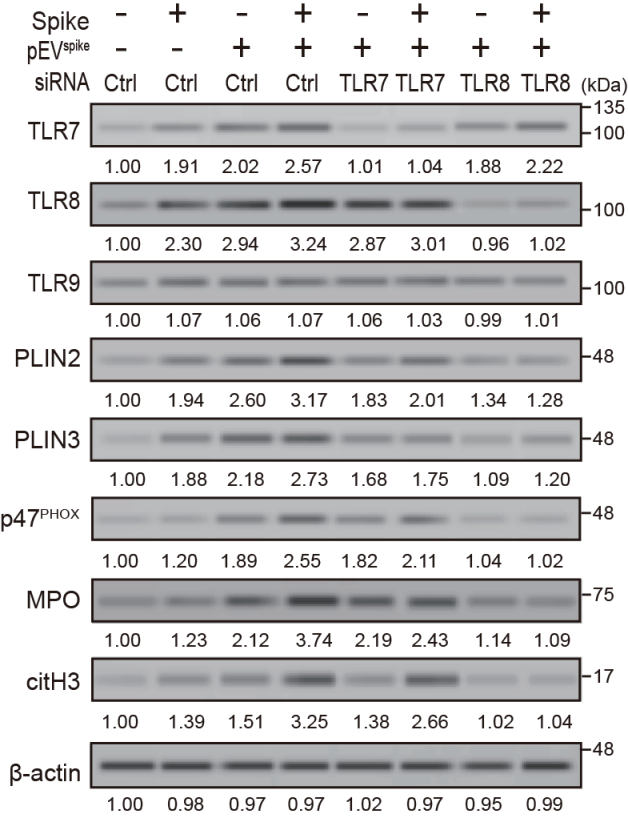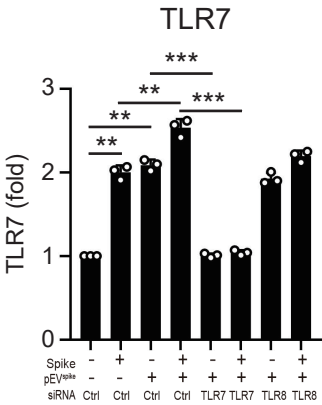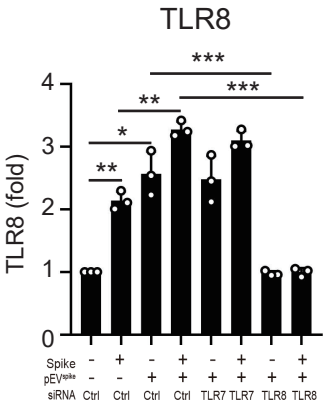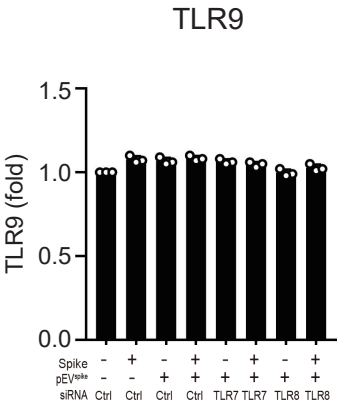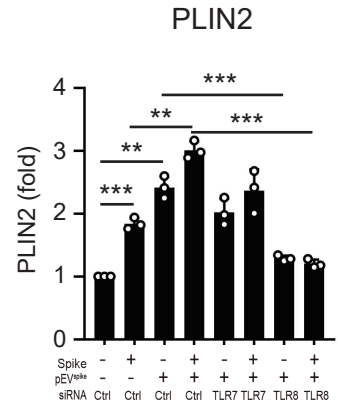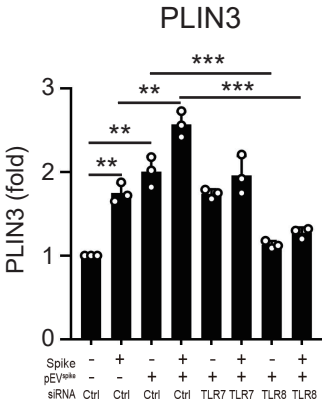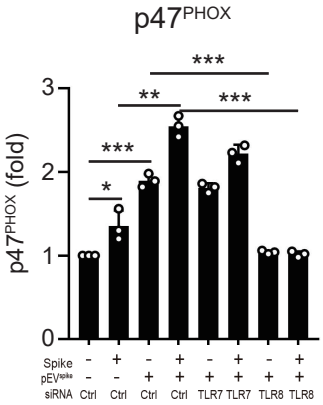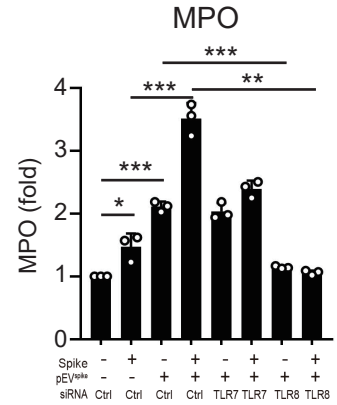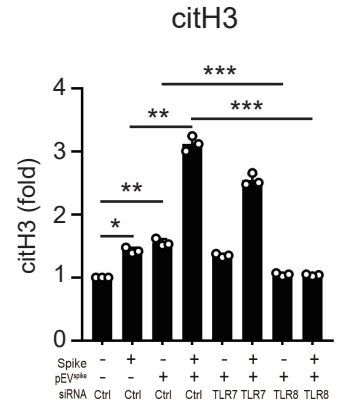

Fig. 3B

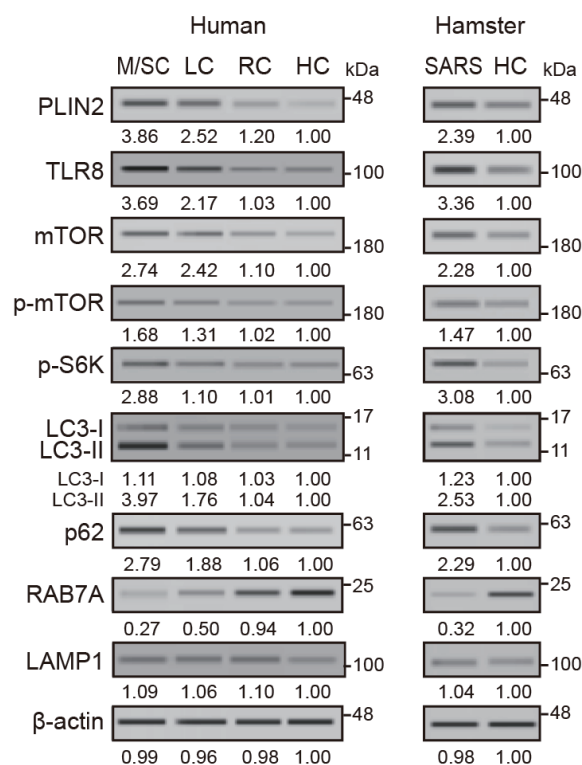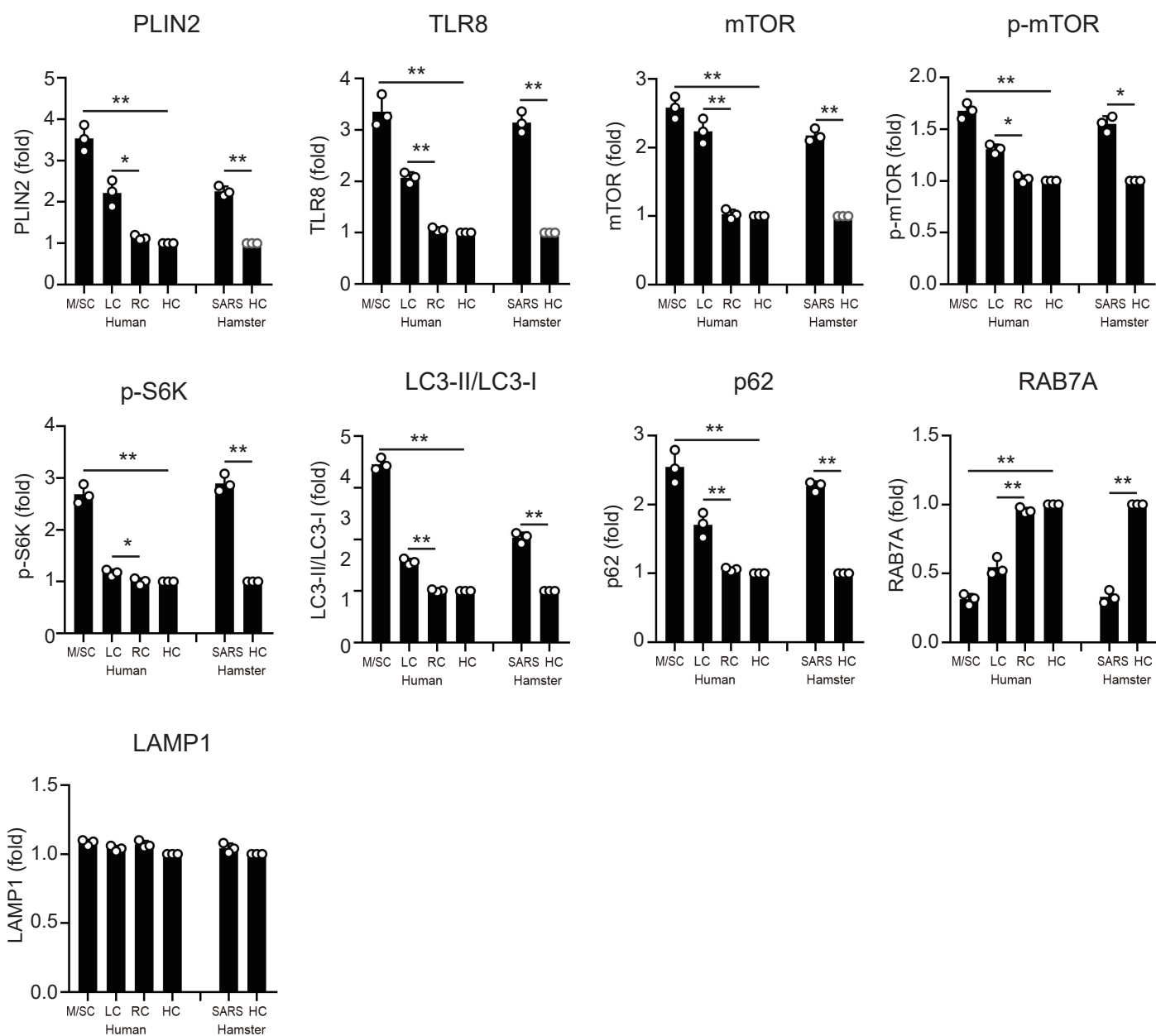

Fig. 3C

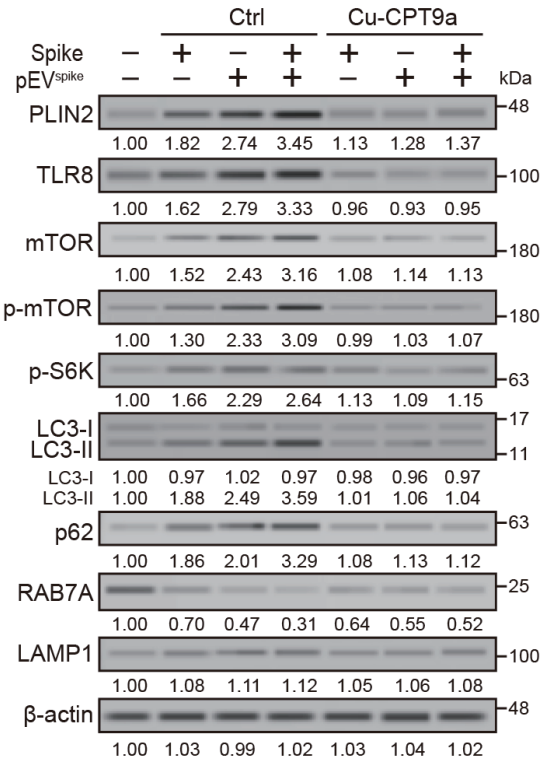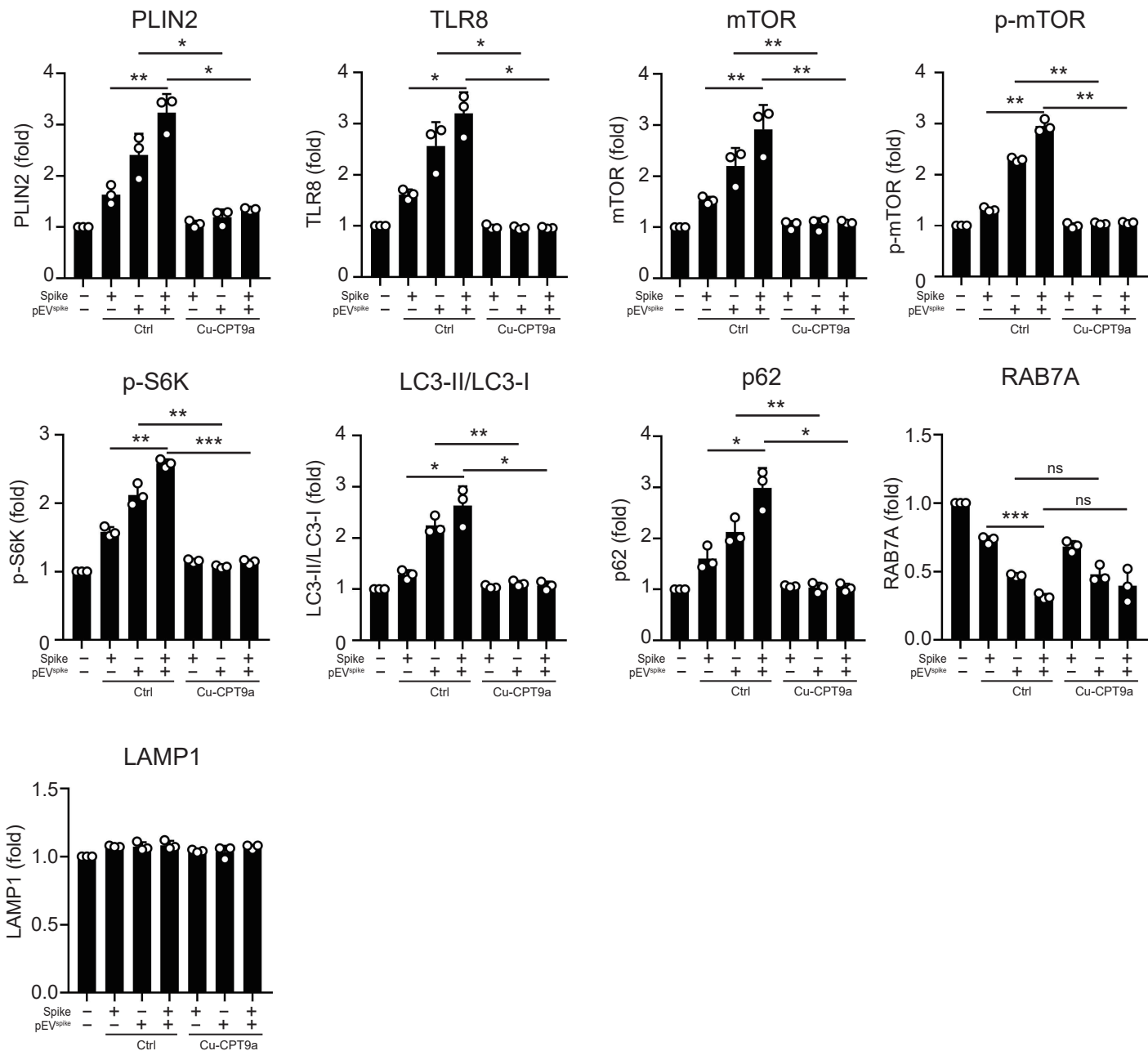

Fig. 3E

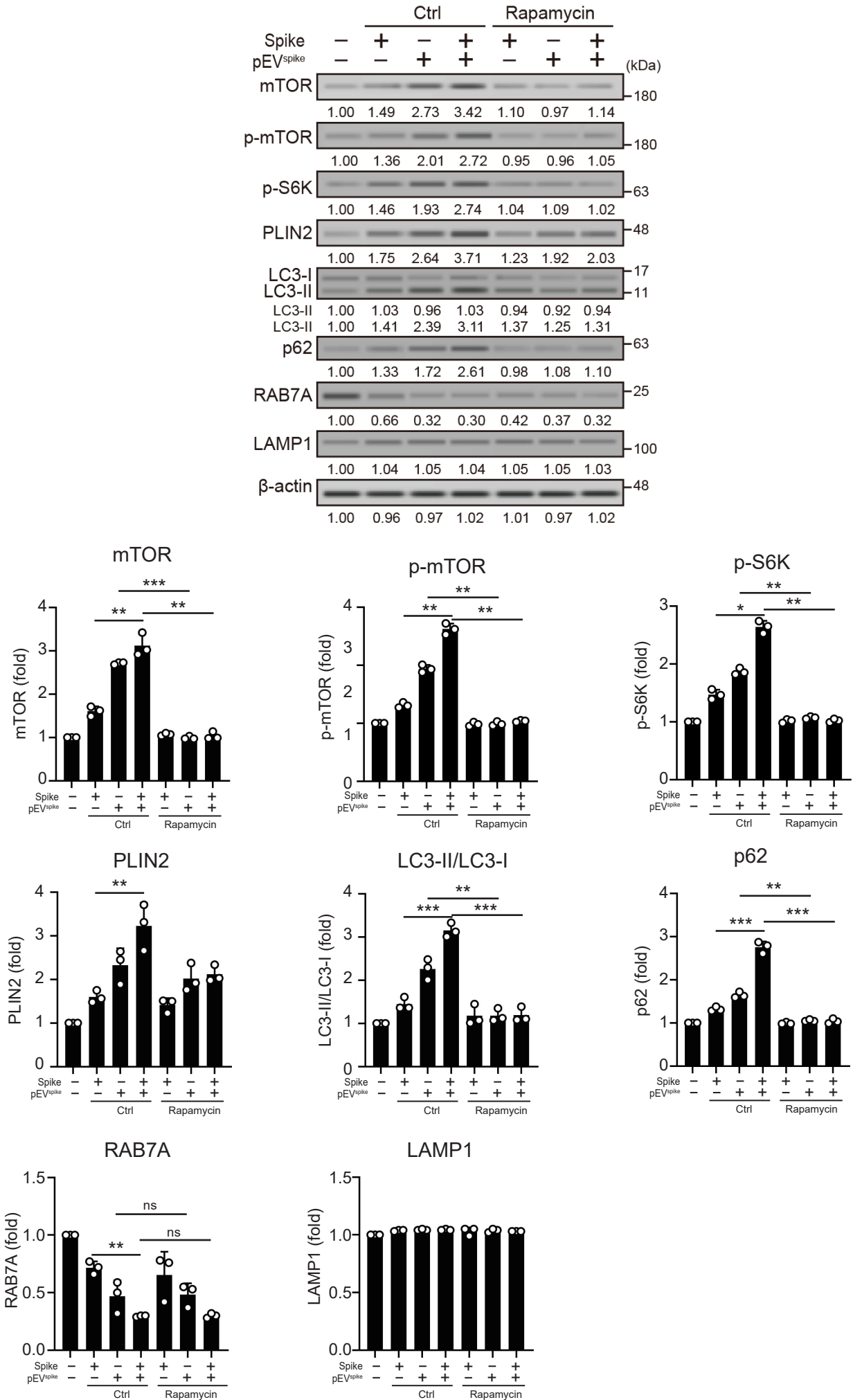

Fig. 4B

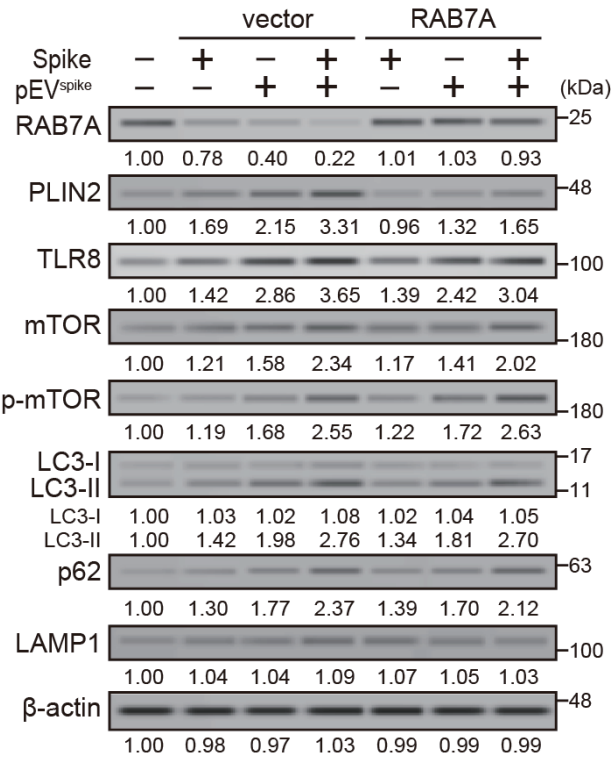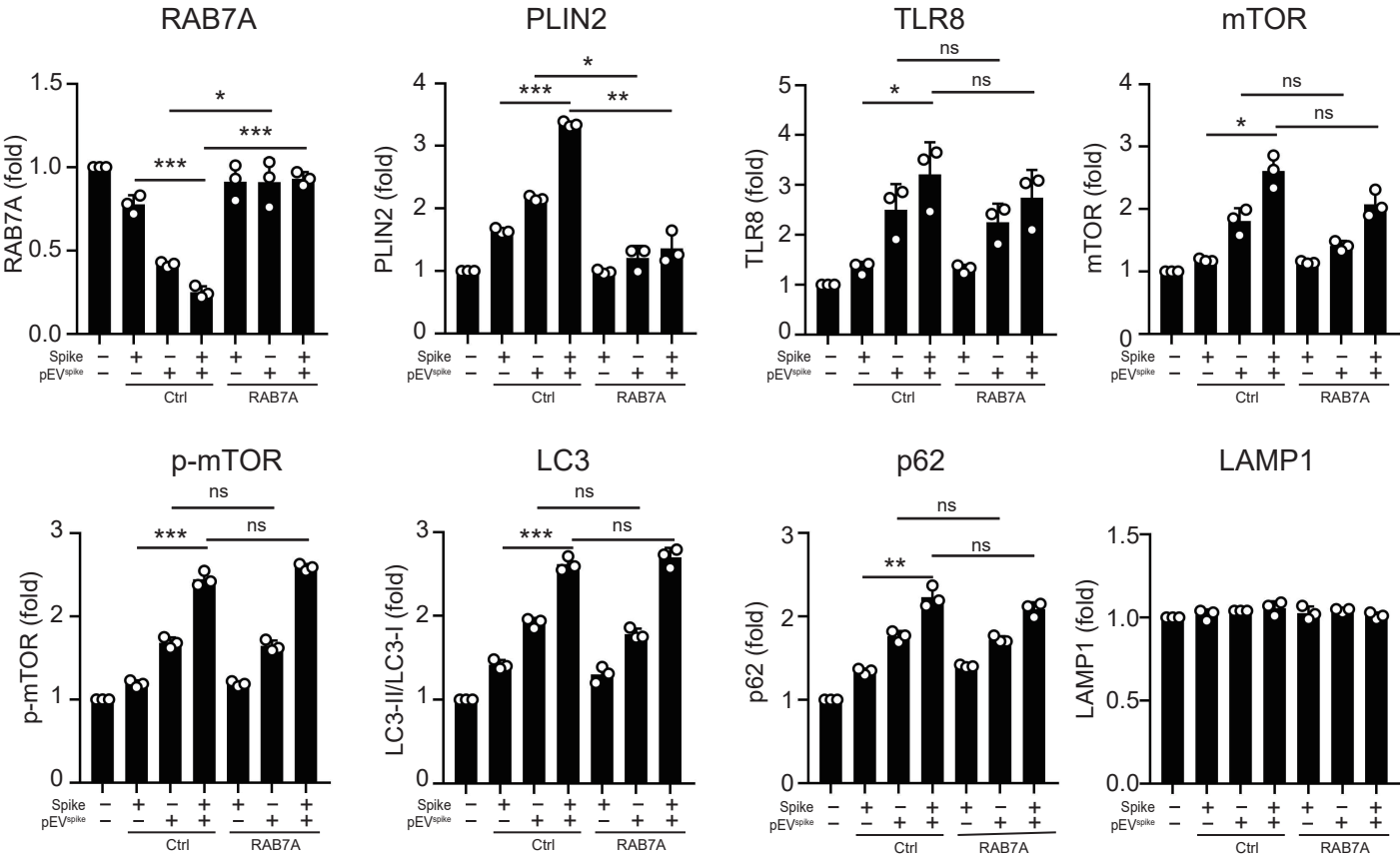

Fig. 5H

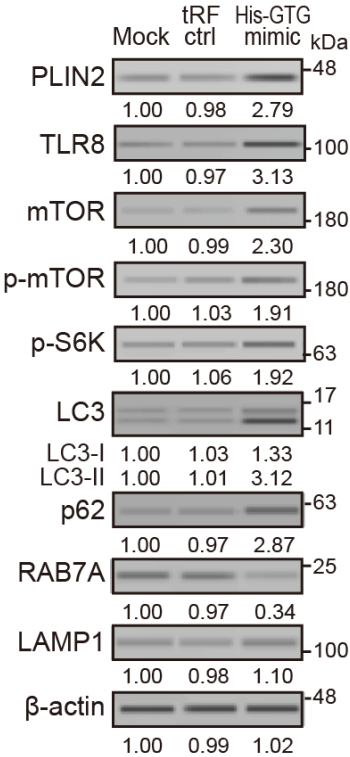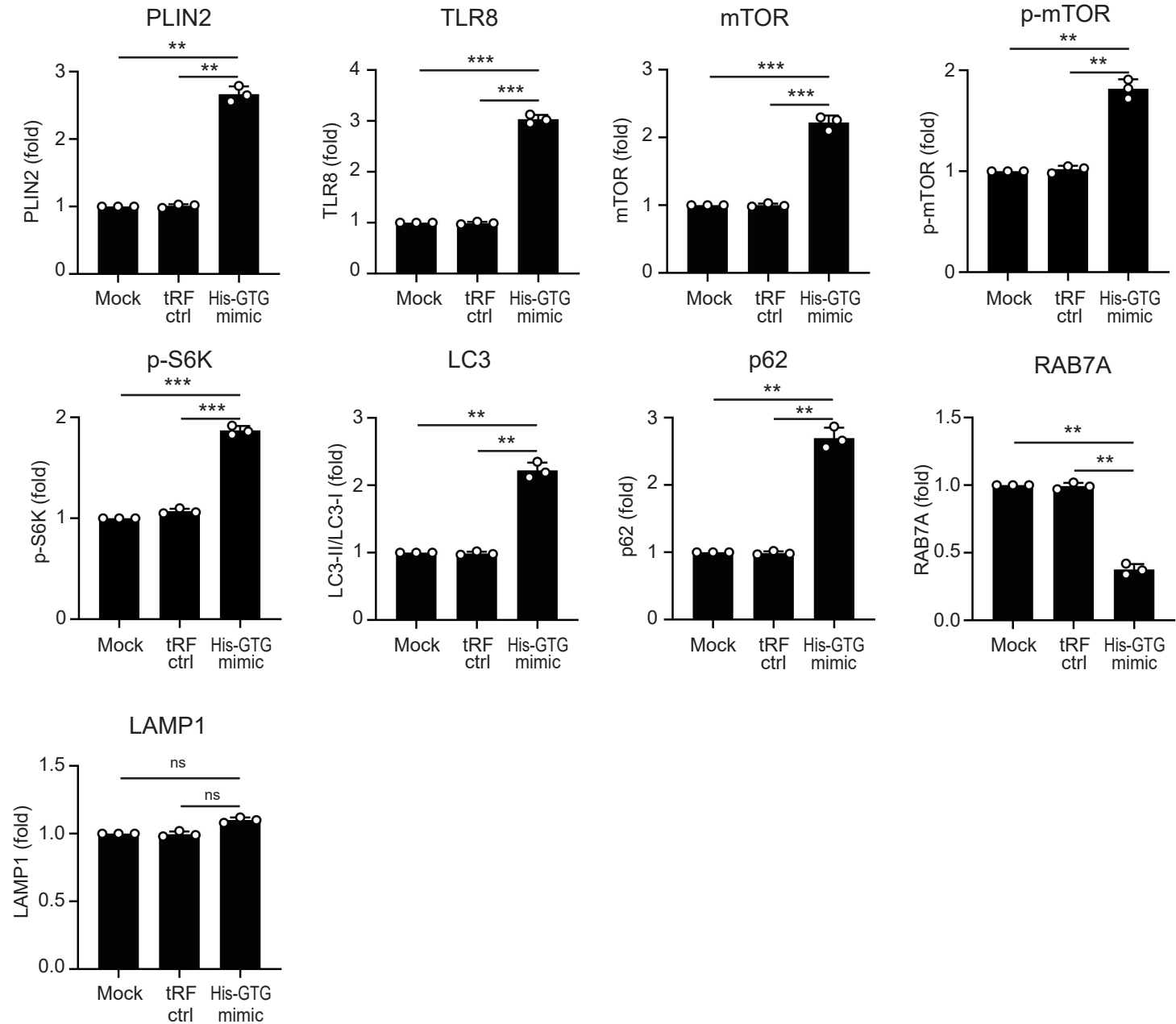

Fig. 6B

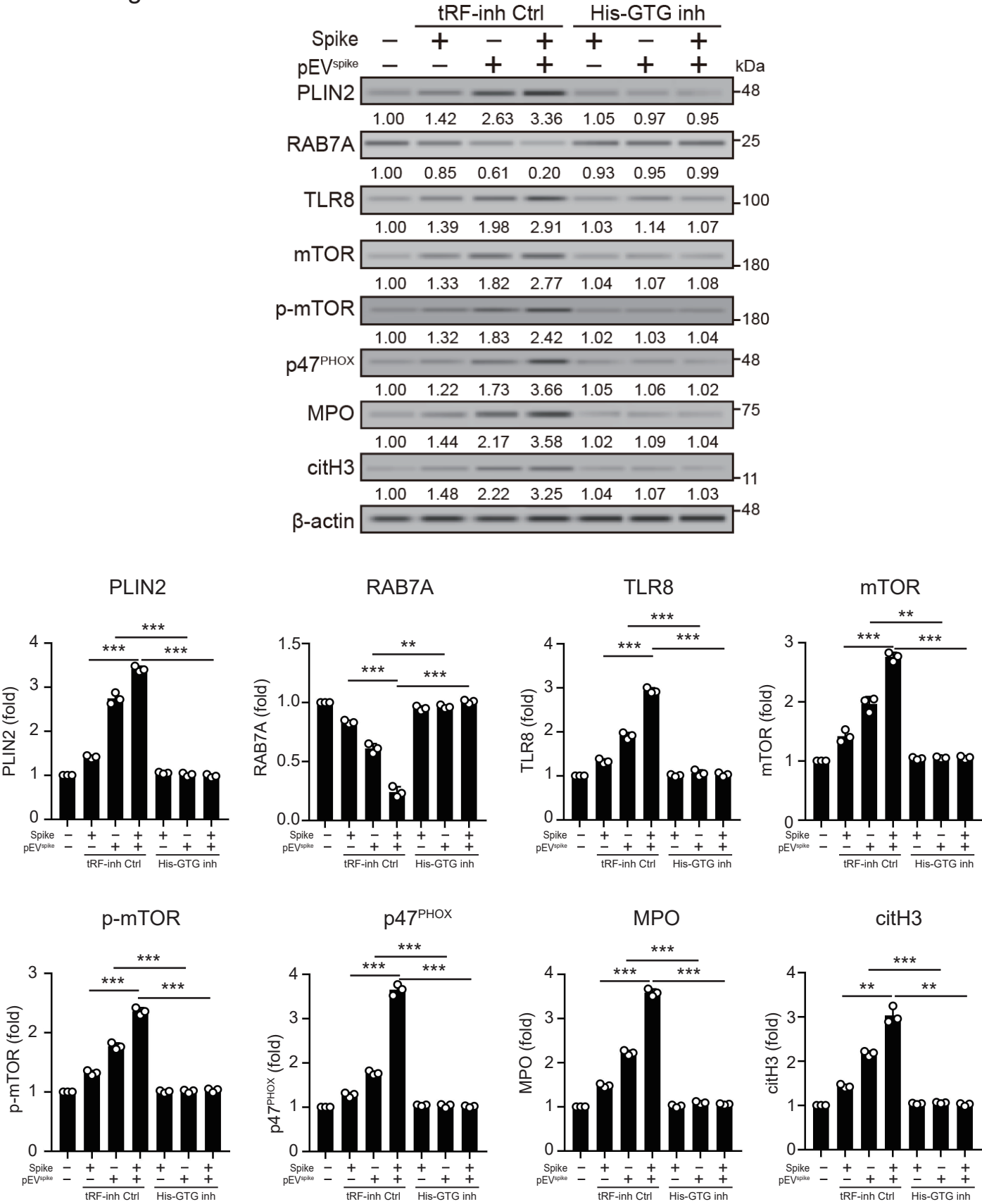

Fig. S2G

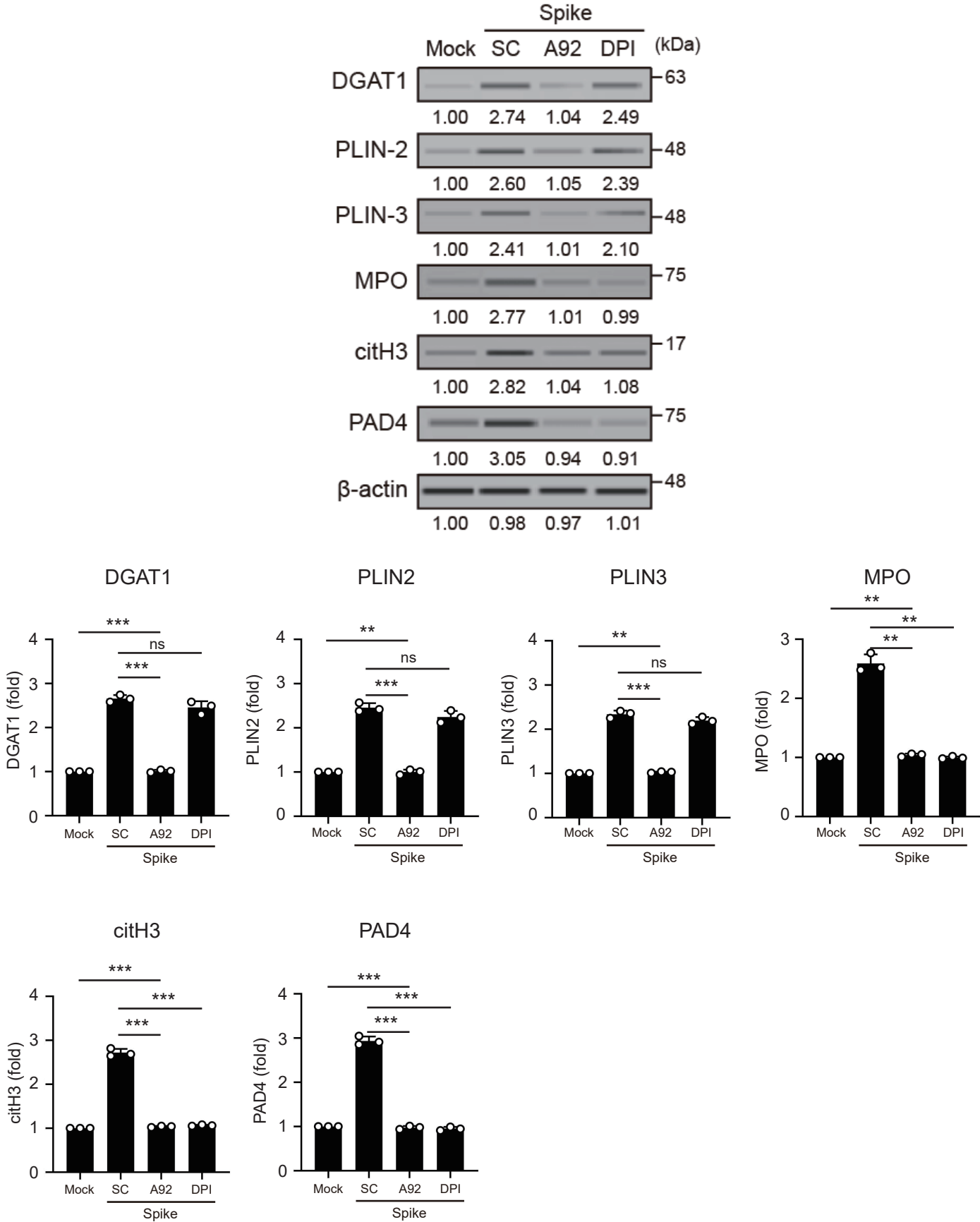

Fig. S10D

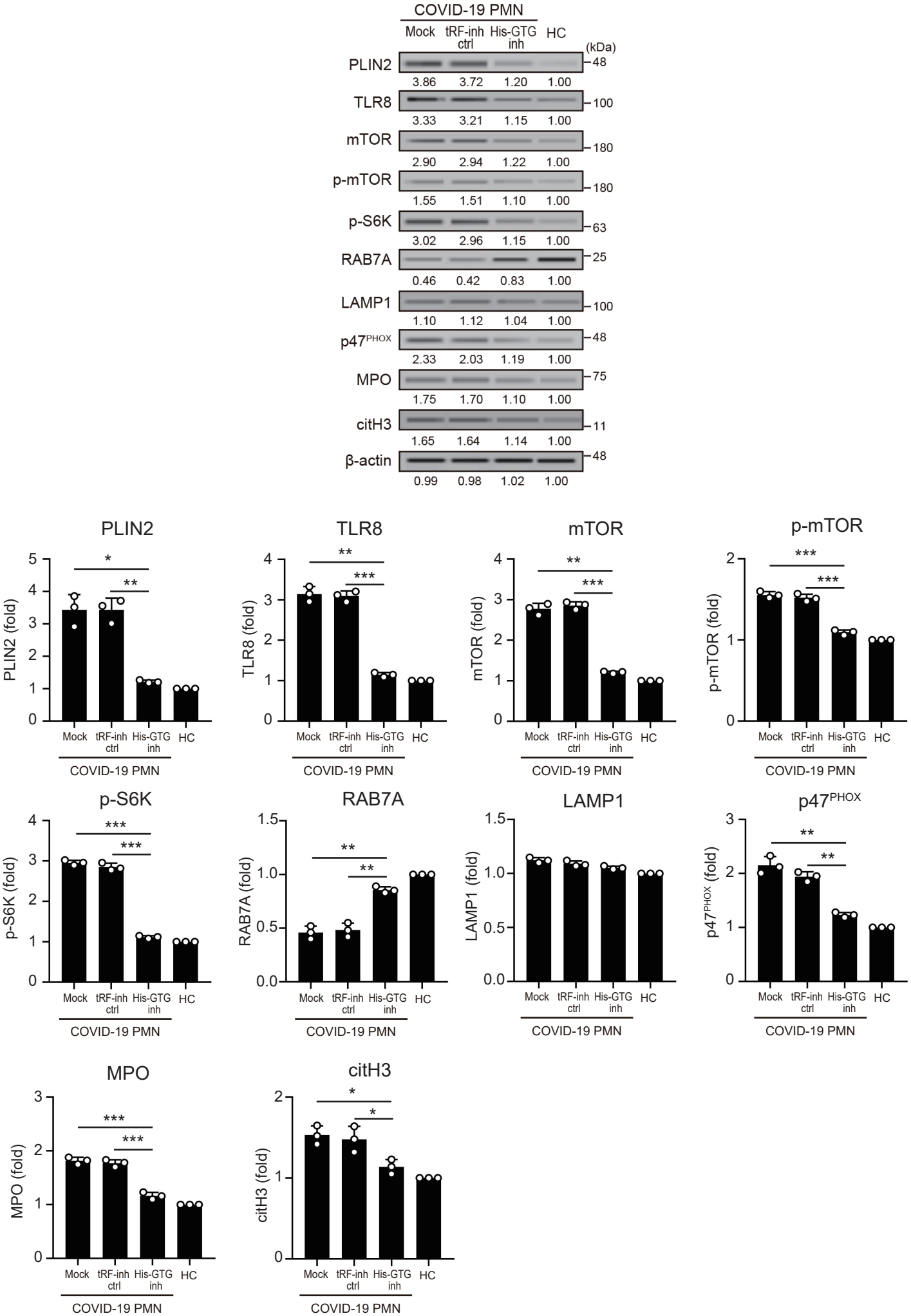

Supplement: Supplementary file 2 — Supporting File 2: advs73502‐sup‐0002‐Additional file 2_Densitometric analysis of immunoblot.pdf. [file ADVS-13-e08695-s001.pdf]
