# Supplementary material for: Extracellular Vesicle‐Delivered tRF‐His‐GTG‐1 Reprograms Neutrophil Lipophagy and Triggers Inflammation in COVID‐19
Source: Adv Sci (Weinh). 2026 Jan 14;13(13):e08695. doi: 10.1002/advs.202508695 (PMC12955898; doi:10.1002/advs.202508695)

Fig. 1J

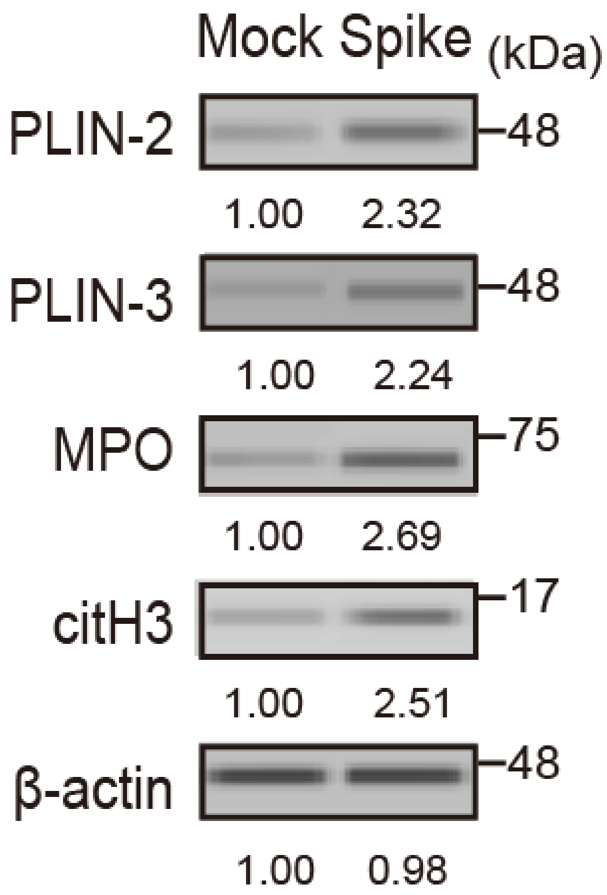

Mock Spike (kDa)
PLIN-2
48
1.00 2.32
PLIN-3
48
1.00 2.24
MPO
75
1.00 2.69
citH3
17
1.00 2.51
β-actin
48
1.00 0.98

PLIN-2 (48 kDa)/ PLIN-3 (47 kDa)/ MPO (72 kDa)/ citH3 (14 kDa)

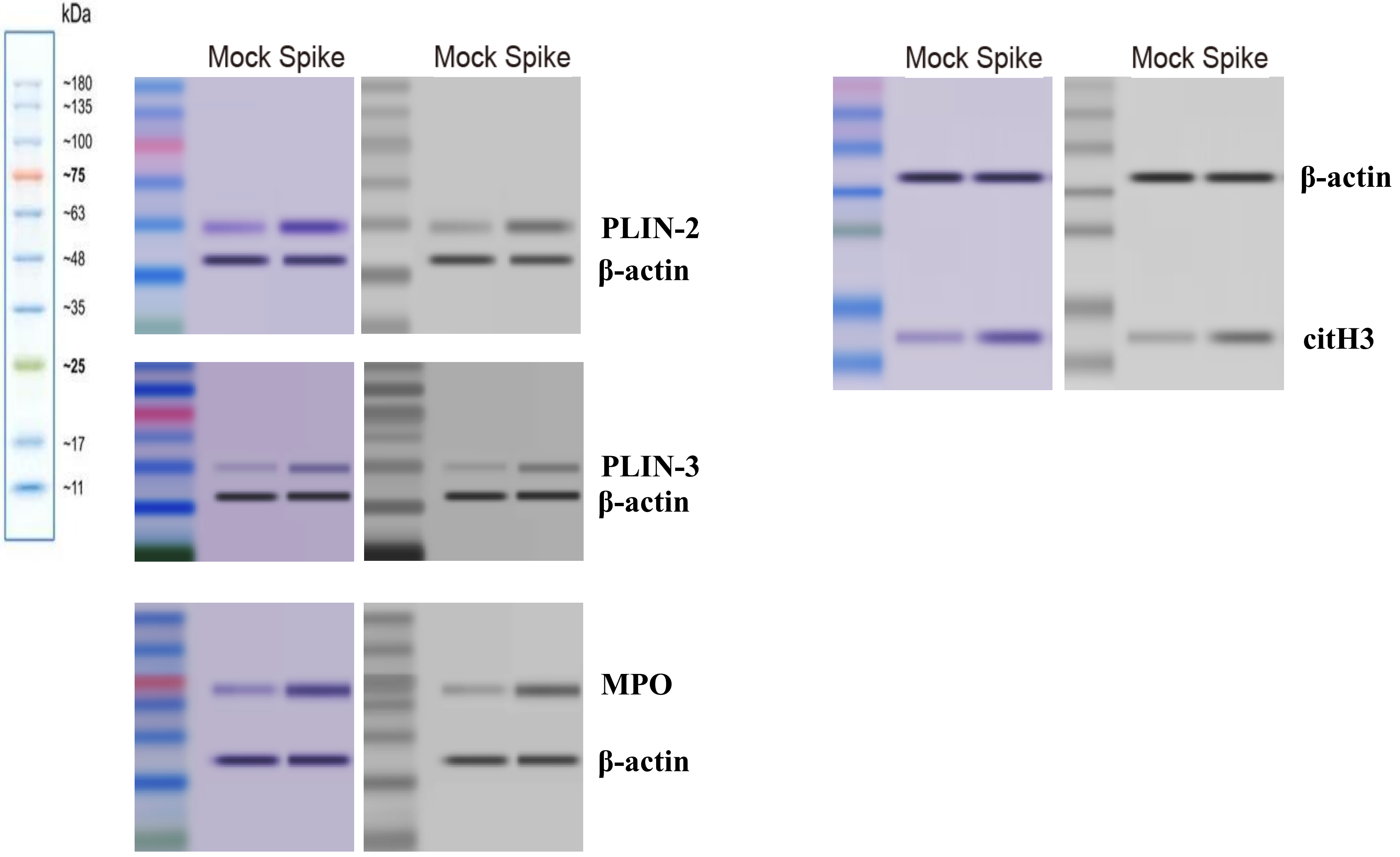

Fig. 2C

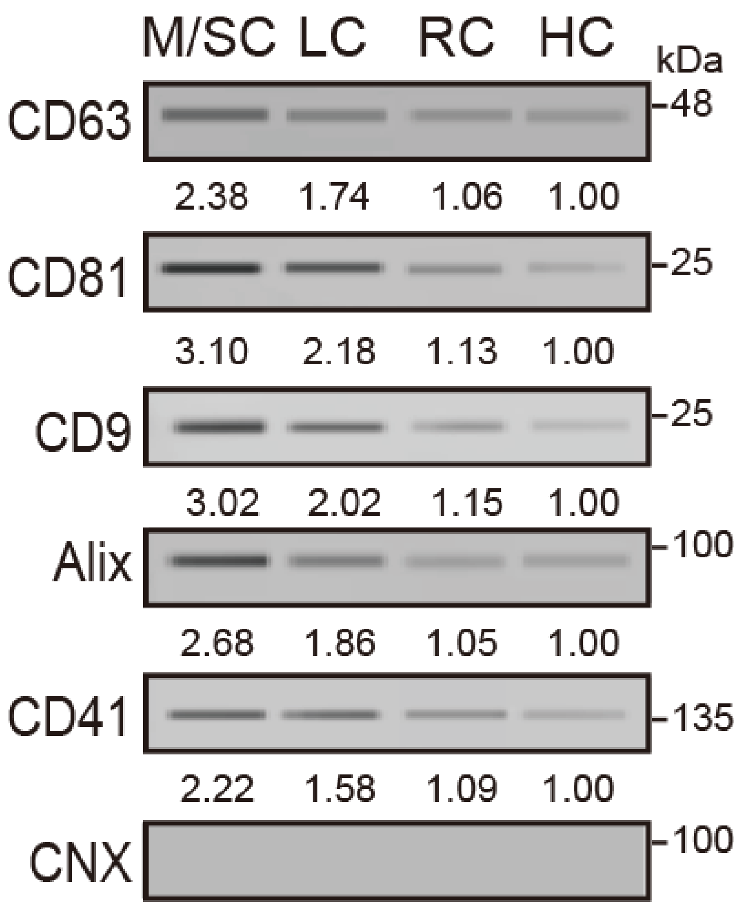
M/SC LC RC HC
kDa
CD63
-48
2.38 1.74 1.06 1.00
CD81
-25
3.10 2.18 1.13 1.00
CD9
-25
3.02 2.02 1.15 1.00
Alix
-100
2.68 1.86 1.05 1.00
CD41
-135
2.22 1.58 1.09 1.00
CNX
-100

CD63 (43 kDa)/ CD81 (25 kDa)/ CD9 (24 kDa)/ Alix (95 kDa)/ CD41 (137 kDa)

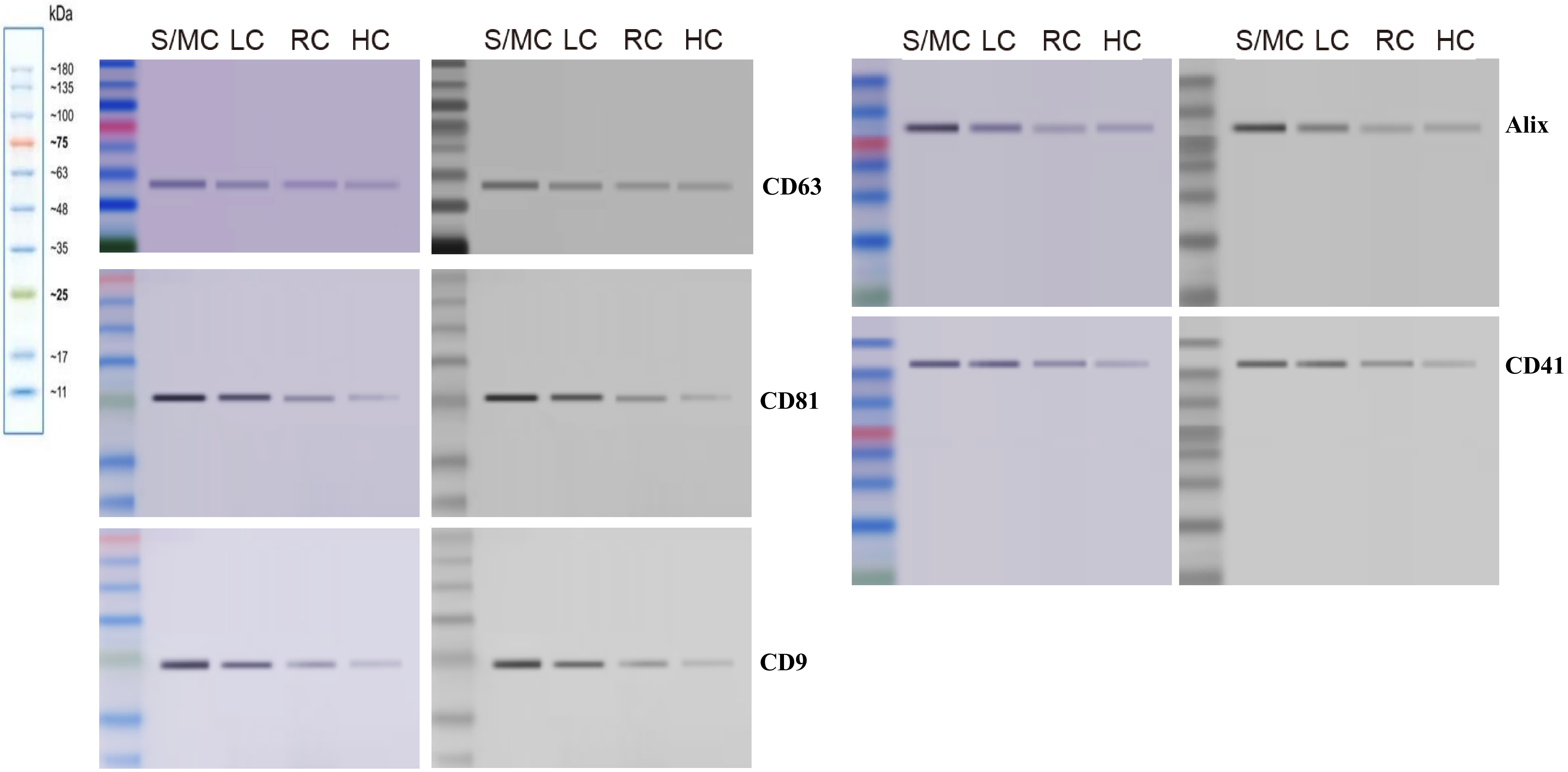

Calnexin (90 kda)

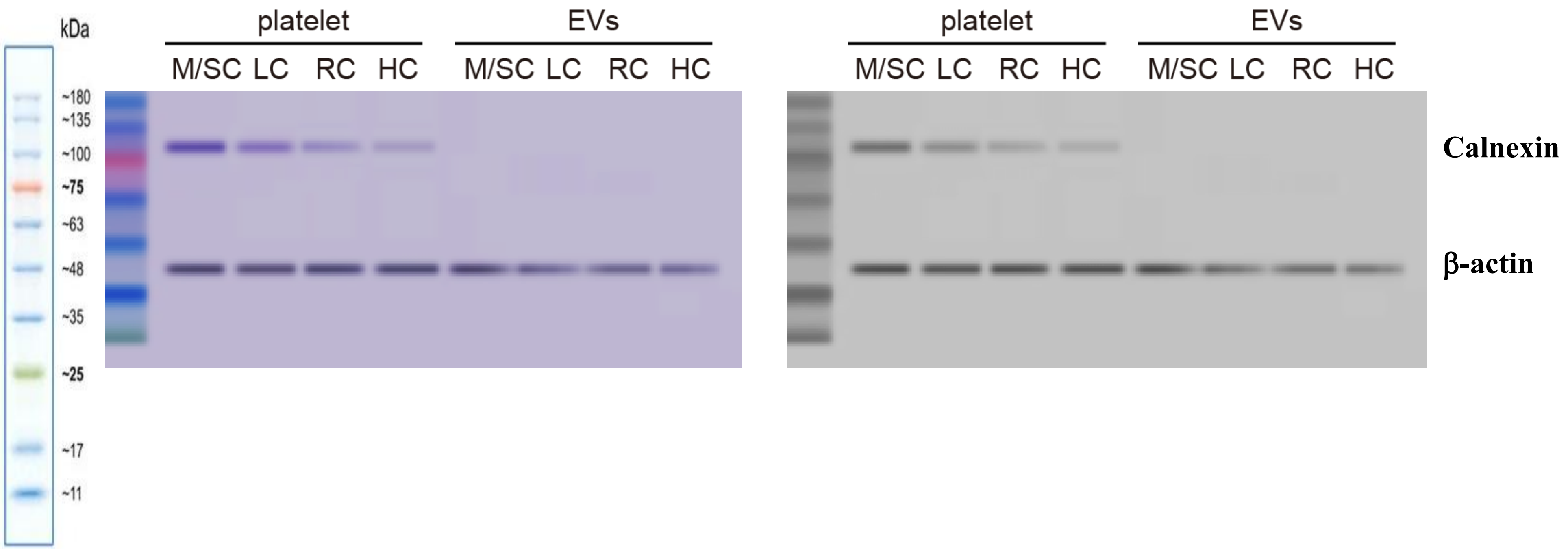

Fig. 2G

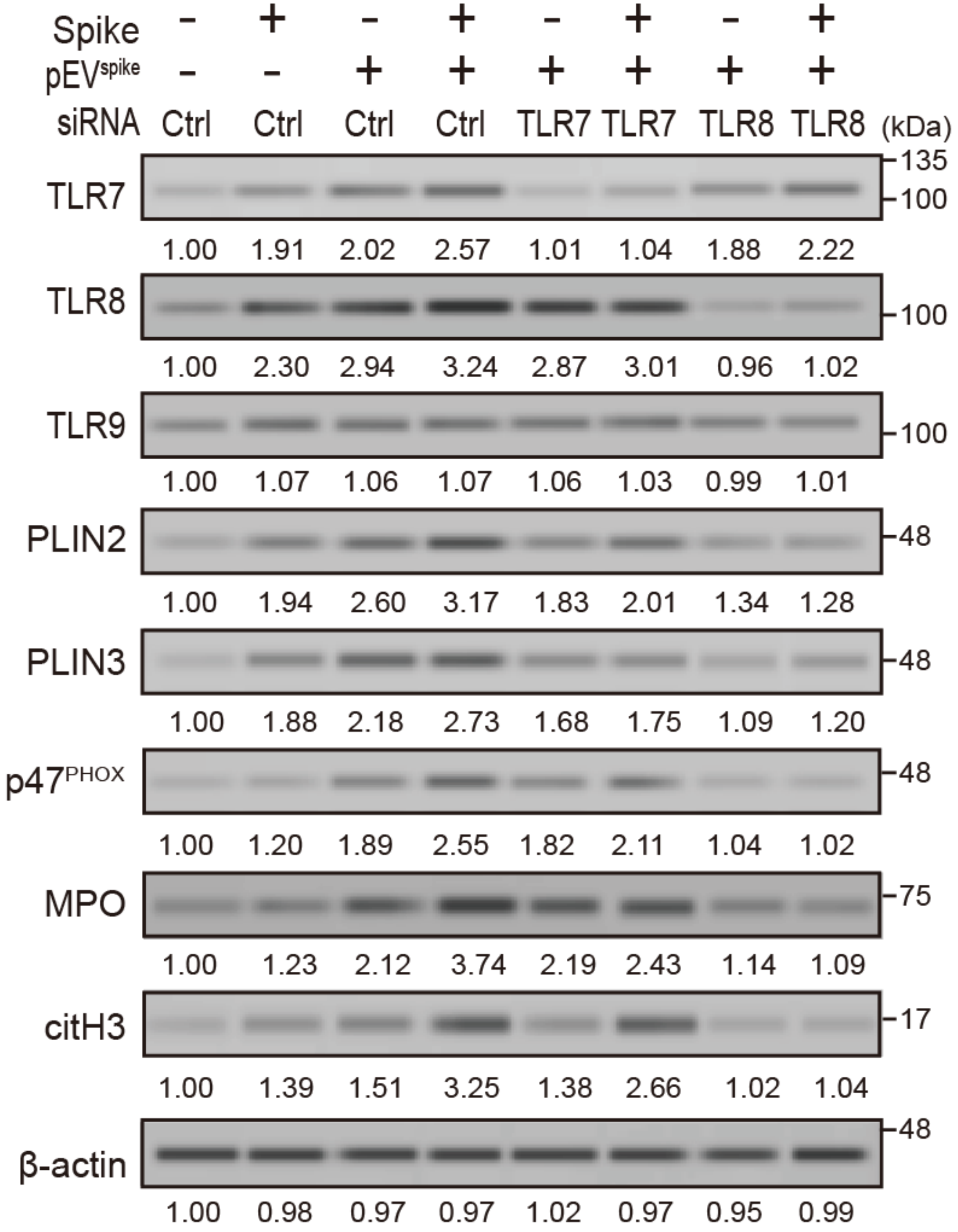
Spike - + - + - + - +
pEVspike - - + + + + + +
siRNA Ctrl Ctrl Ctrl Ctrl TLR7 TLR7 TLR8 TLR8
(kDa)
TLR7
135
100
1.00 1.91 2.02 2.57 1.01 1.04 1.88 2.22
TLR8
100
1.00 2.30 2.94 3.24 2.87 3.01 0.96 1.02
TLR9
100
1.00 1.07 1.06 1.07 1.06 1.03 0.99 1.01
PLIN2
48
1.00 1.94 2.60 3.17 1.83 2.01 1.34 1.28
PLIN3
48
1.00 1.88 2.18 2.73 1.68 1.75 1.09 1.20
p47PHOX
48
1.00 1.20 1.89 2.55 1.82 2.11 1.04 1.02
MPO
75
1.00 1.23 2.12 3.74 2.19 2.43 1.14 1.09
citH3
17
1.00 1.39 1.51 3.25 1.38 2.66 1.02 1.04
β-actin
48
1.00 0.98 0.97 0.97 1.02 0.97 0.95 0.99

TLR7 (121 kDa)/ TLR8 (110 kDa)/ TLR9 (113 kDa)

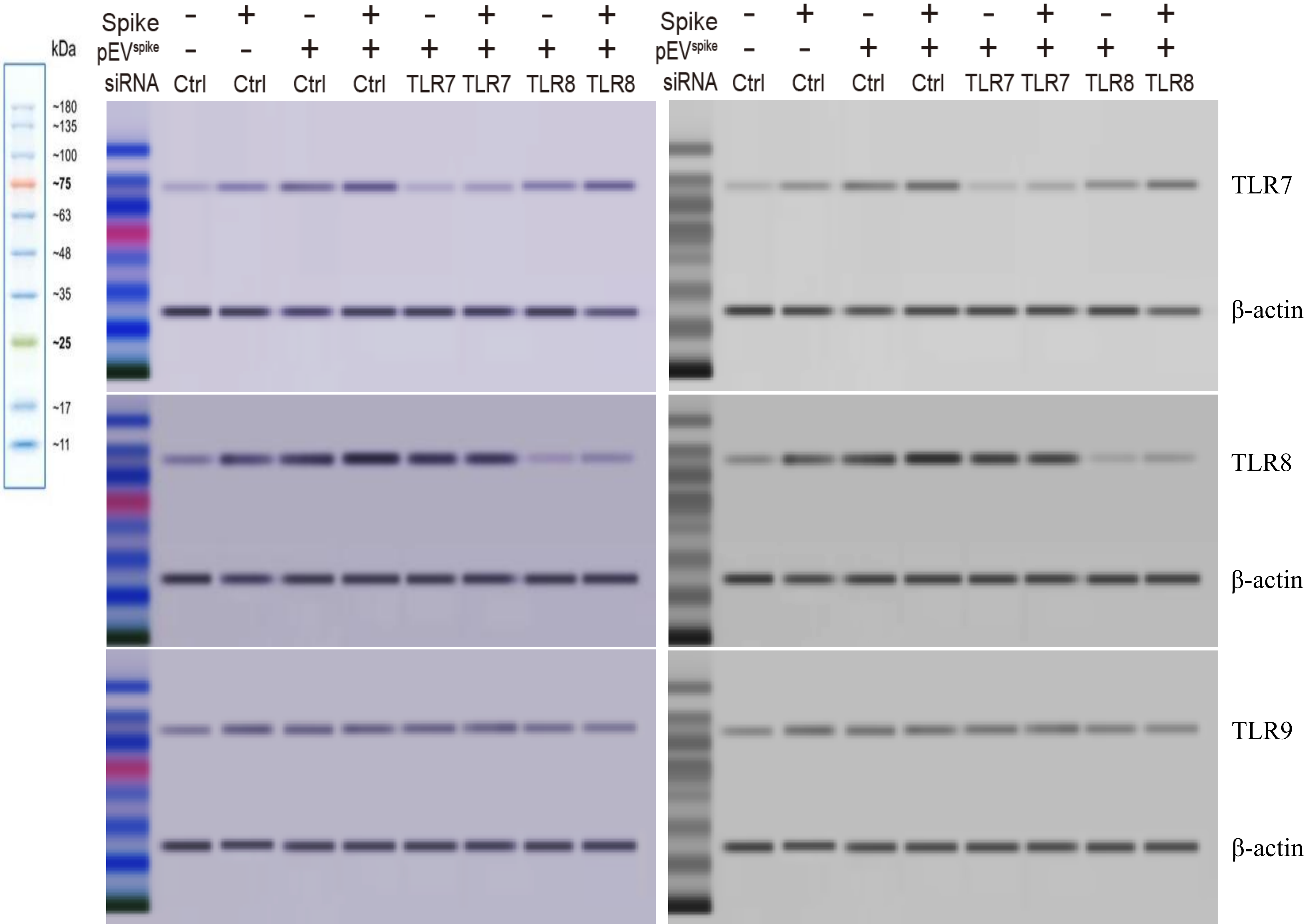

PLIN-2 (48 kDa)/ PLIN-3 (47 kDa)/ p47^{PHOX} (47 kDa)

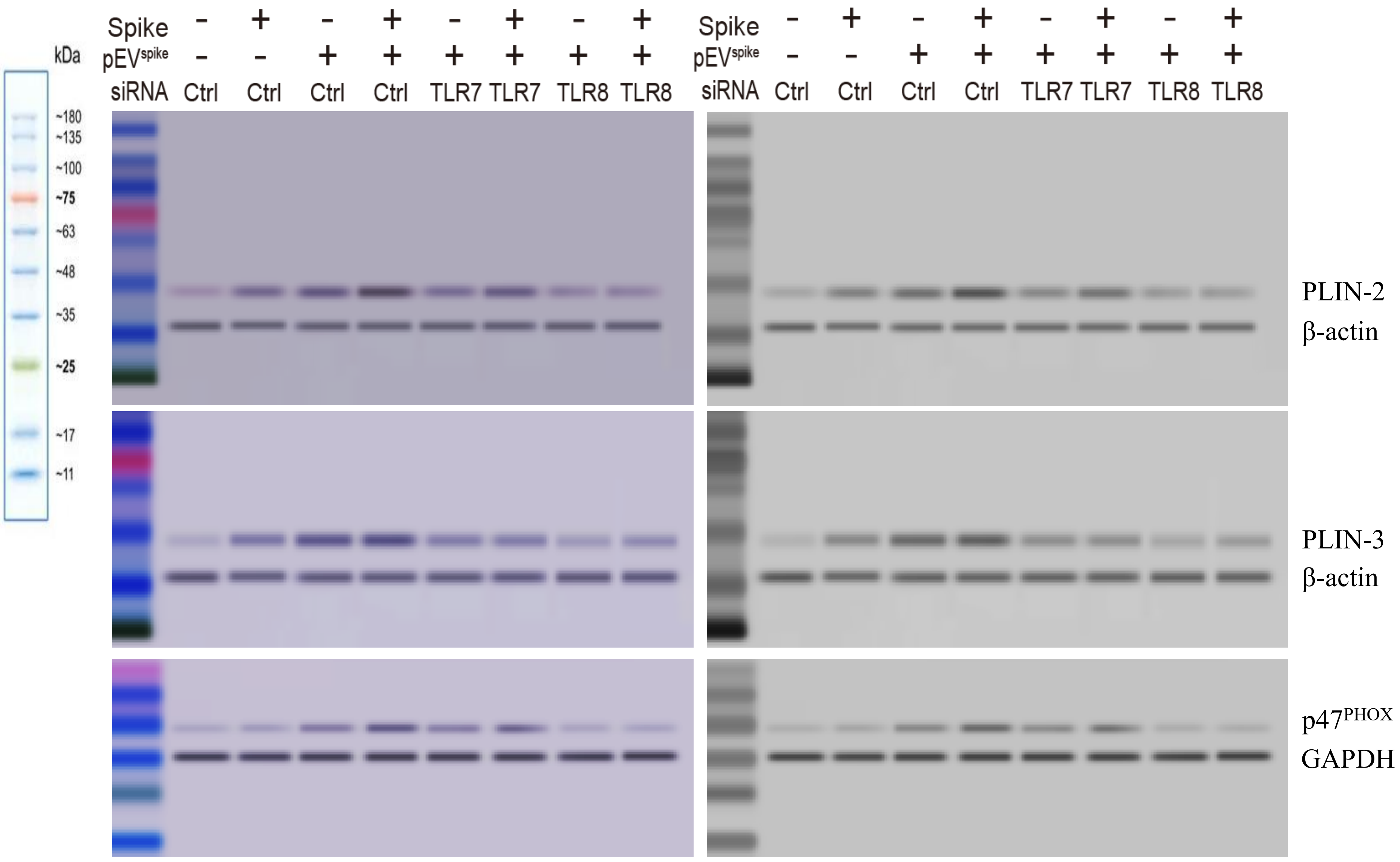

MPO (72 kDa)/ citH3 (14 kDa)

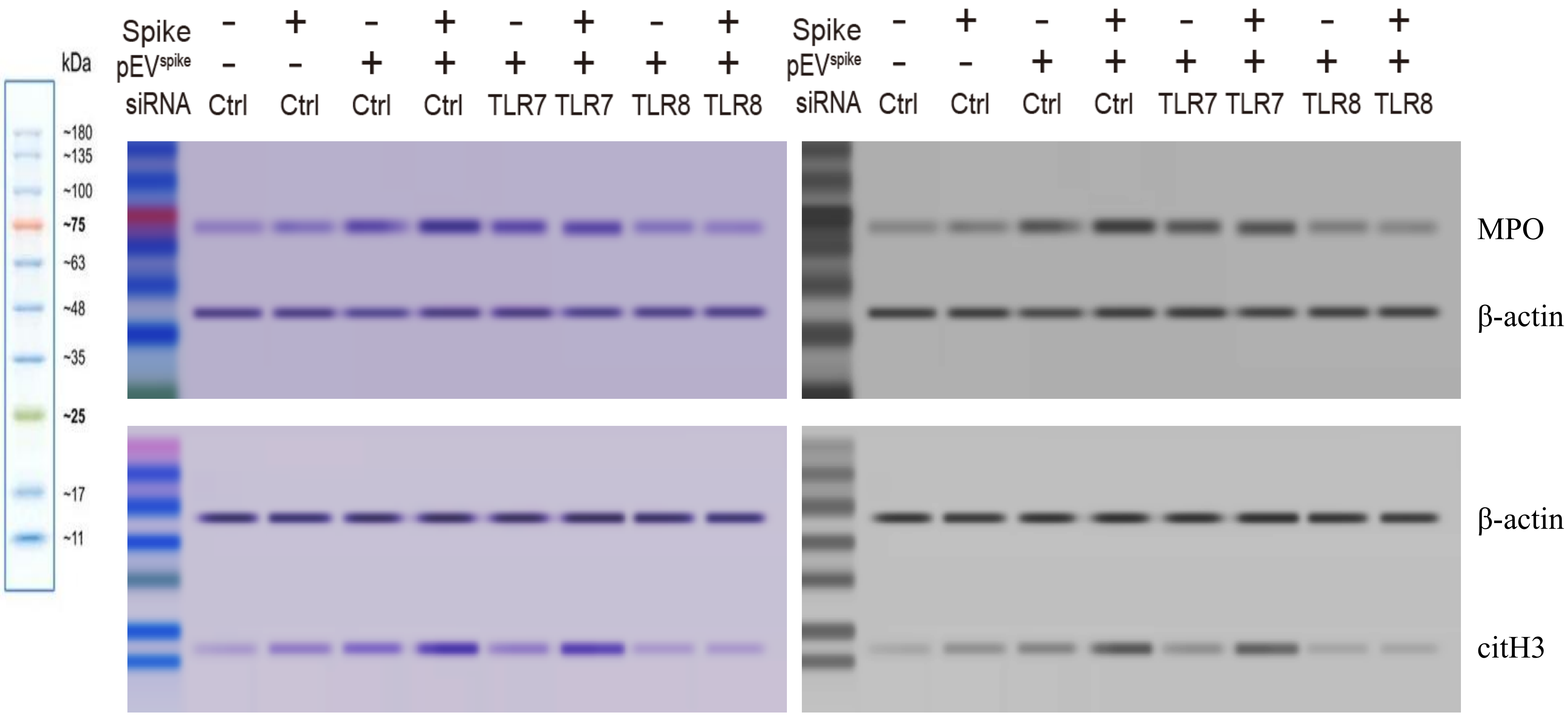

Fig. 3B

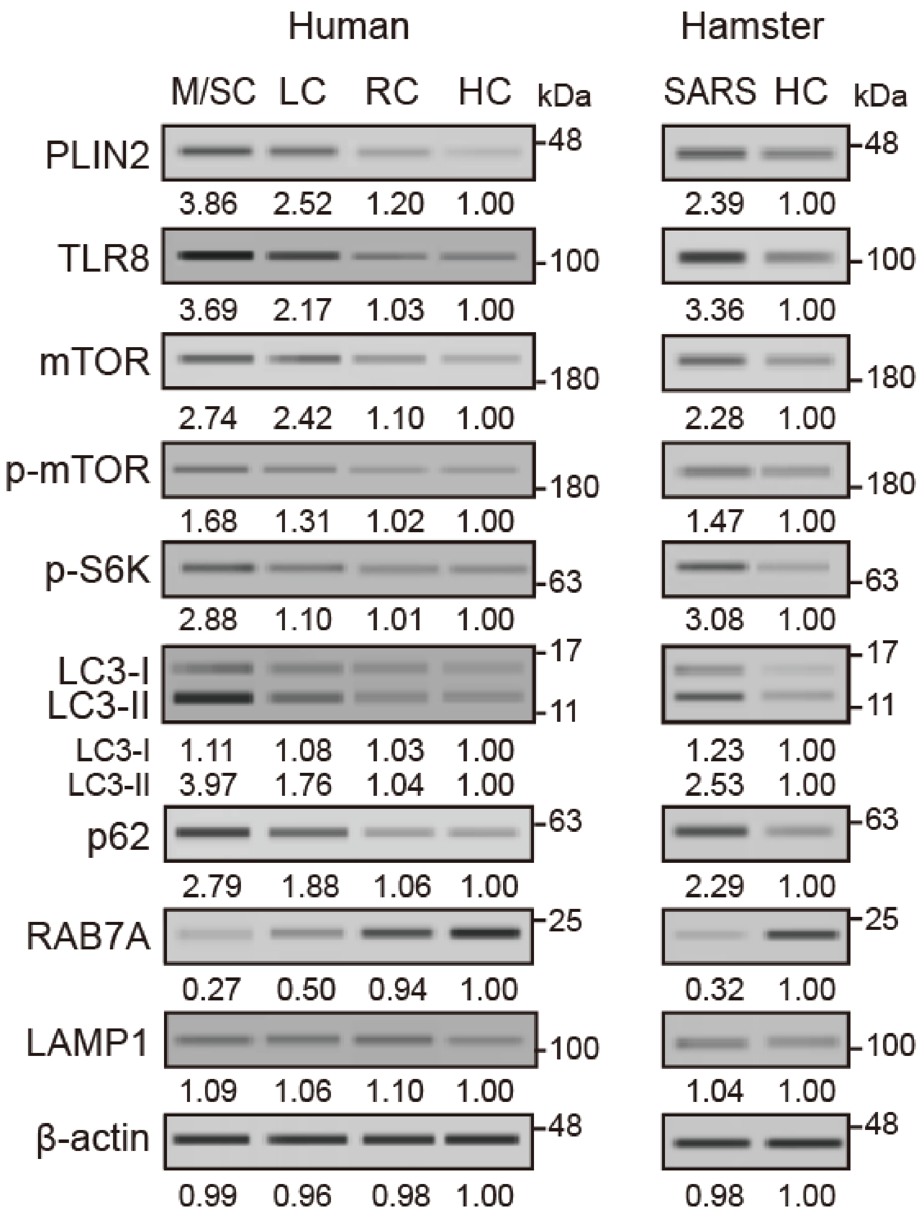
Human
Hamster
M/SC LC RC HC kDa
SARS HC kDa
PLIN2
3.86 2.52 1.20 1.00
2.39 1.00
TLR8
3.69 2.17 1.03 1.00
3.36 1.00
mTOR
2.74 2.42 1.10 1.00
2.28 1.00
p-mTOR
1.68 1.31 1.02 1.00
1.47 1.00
p-S6K
2.88 1.10 1.01 1.00
3.08 1.00
LC3-I
LC3-II
LC3-I 1.11 1.08 1.03 1.00
LC3-II 3.97 1.76 1.04 1.00
1.23 1.00
2.53 1.00
p62
2.79 1.88 1.06 1.00
2.29 1.00
RAB7A
0.27 0.50 0.94 1.00
0.32 1.00
LAMP1
1.09 1.06 1.10 1.00
1.04 1.00
β-actin
0.99 0.96 0.98 1.00
0.98 1.00
48
100
180
63
17
11
25

PLIN2(48 kDa)/ TLR8 (110 kDa)

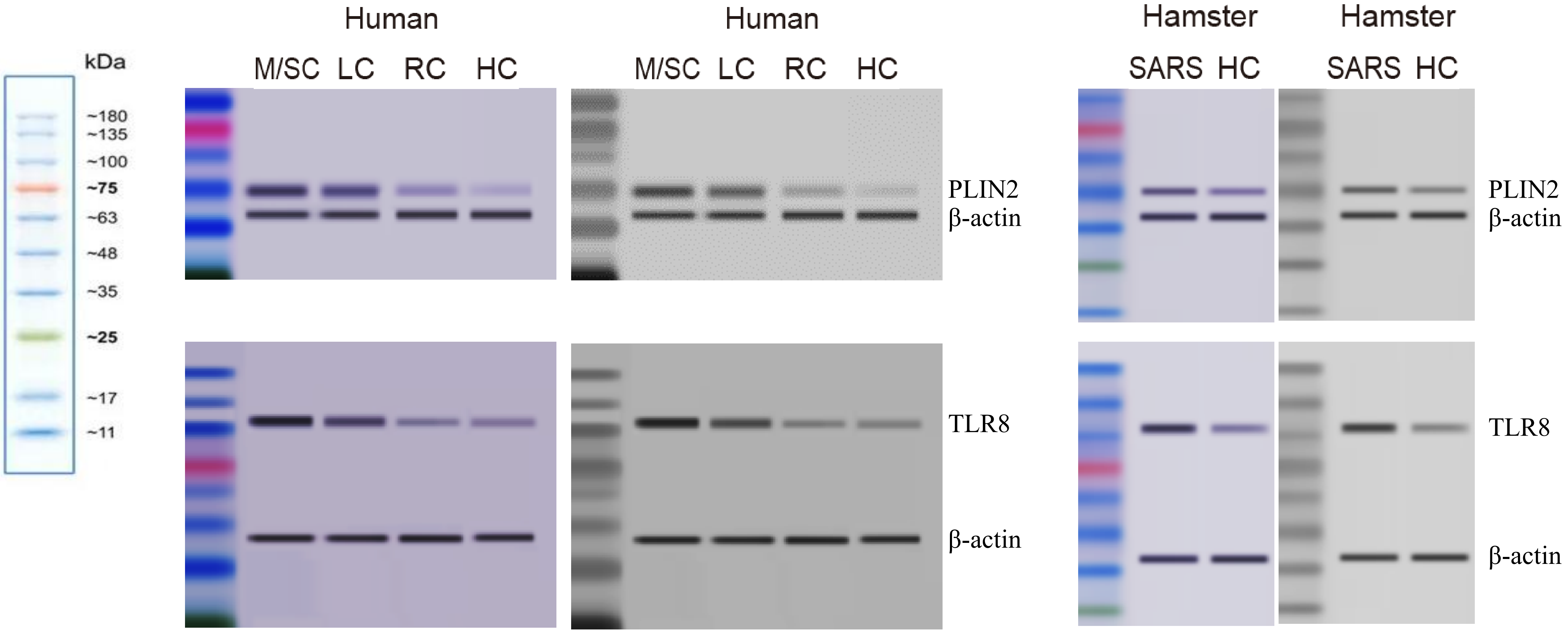

mTOR (289 kDa)/p-mTOR (289 kDa)/p-S6K (70 kDa)

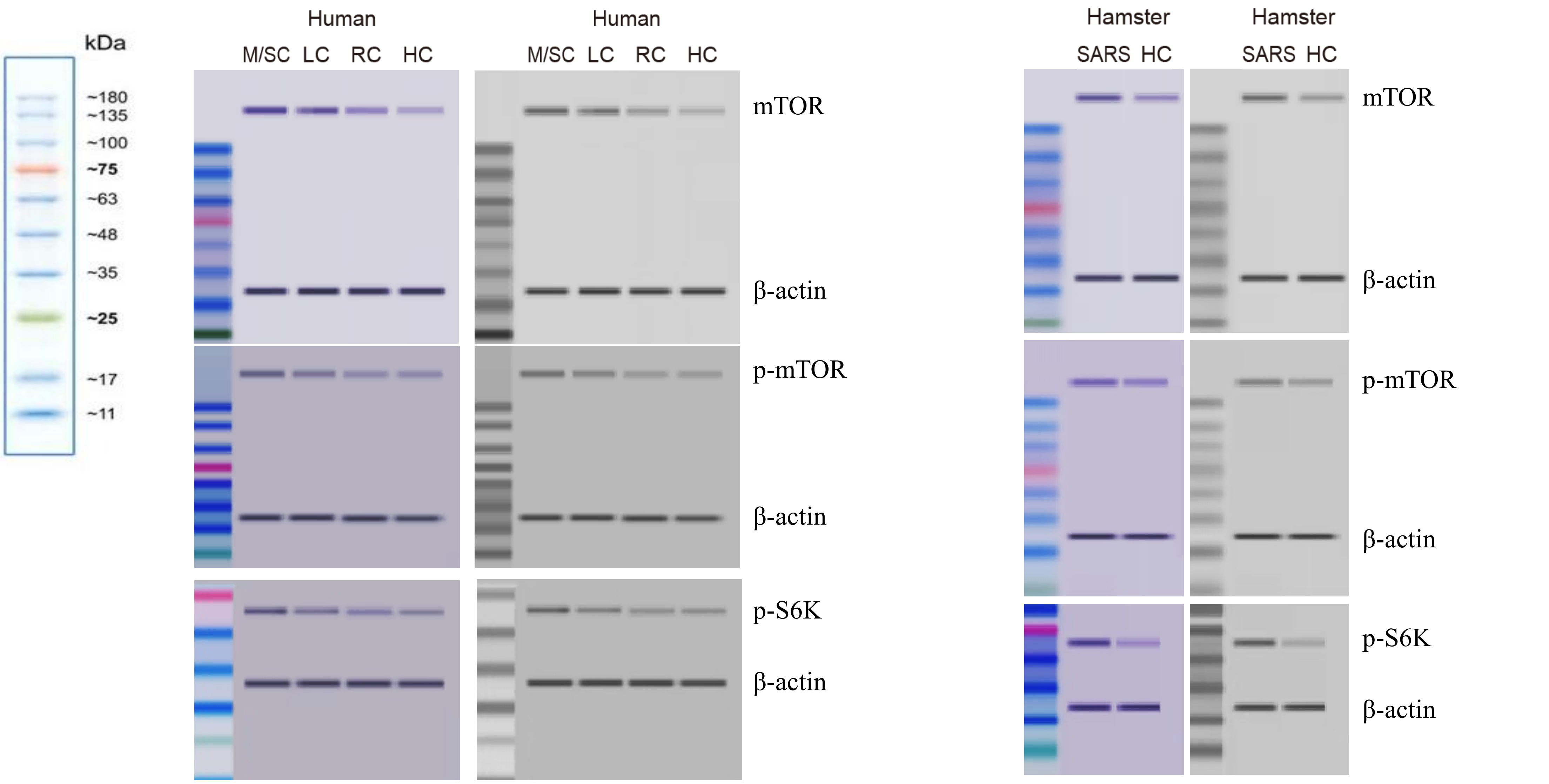

LC3 (14, 16 kDa)/p62 (62 kDa)

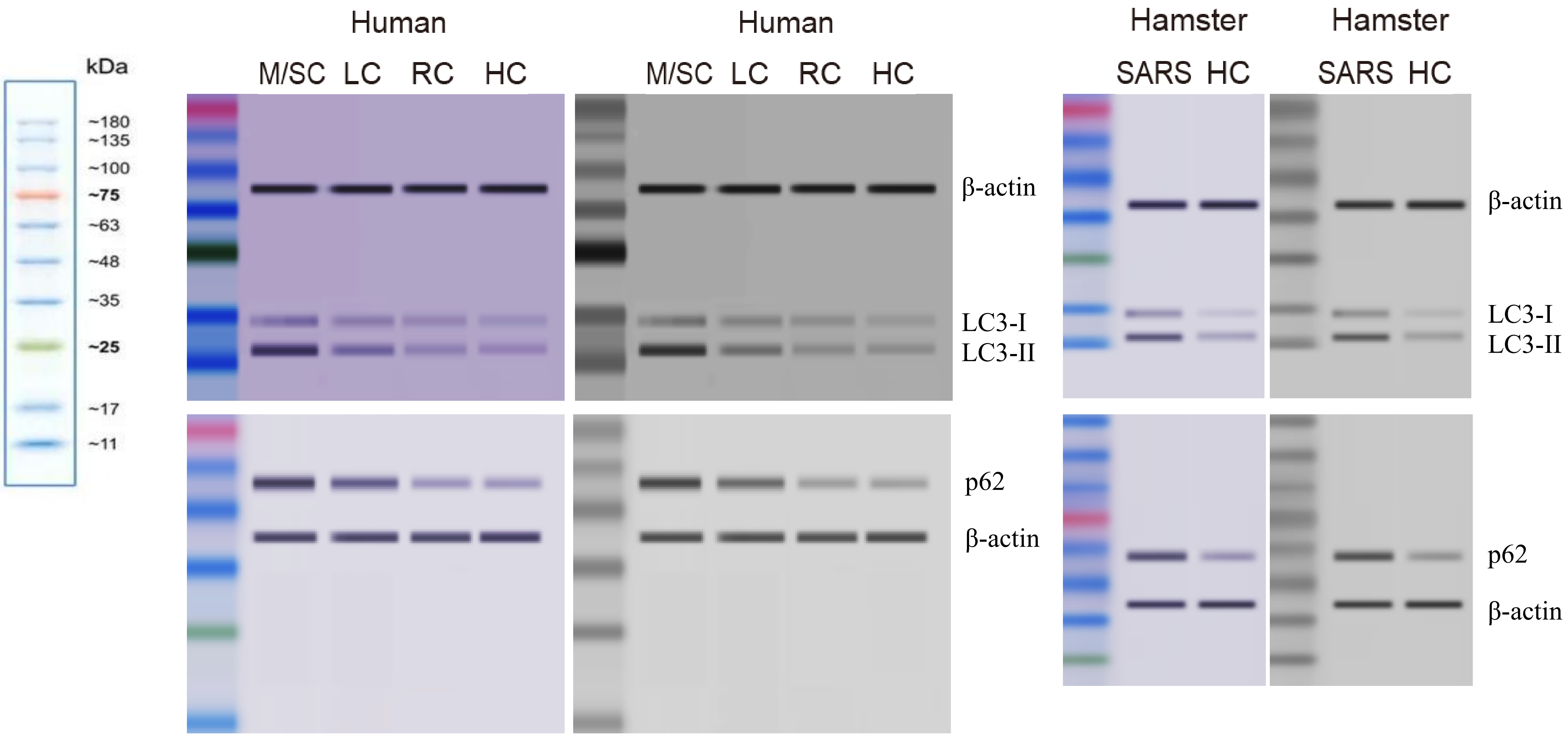

RAB7A (23 kDa)/LAMP1 (100~120 kDa)

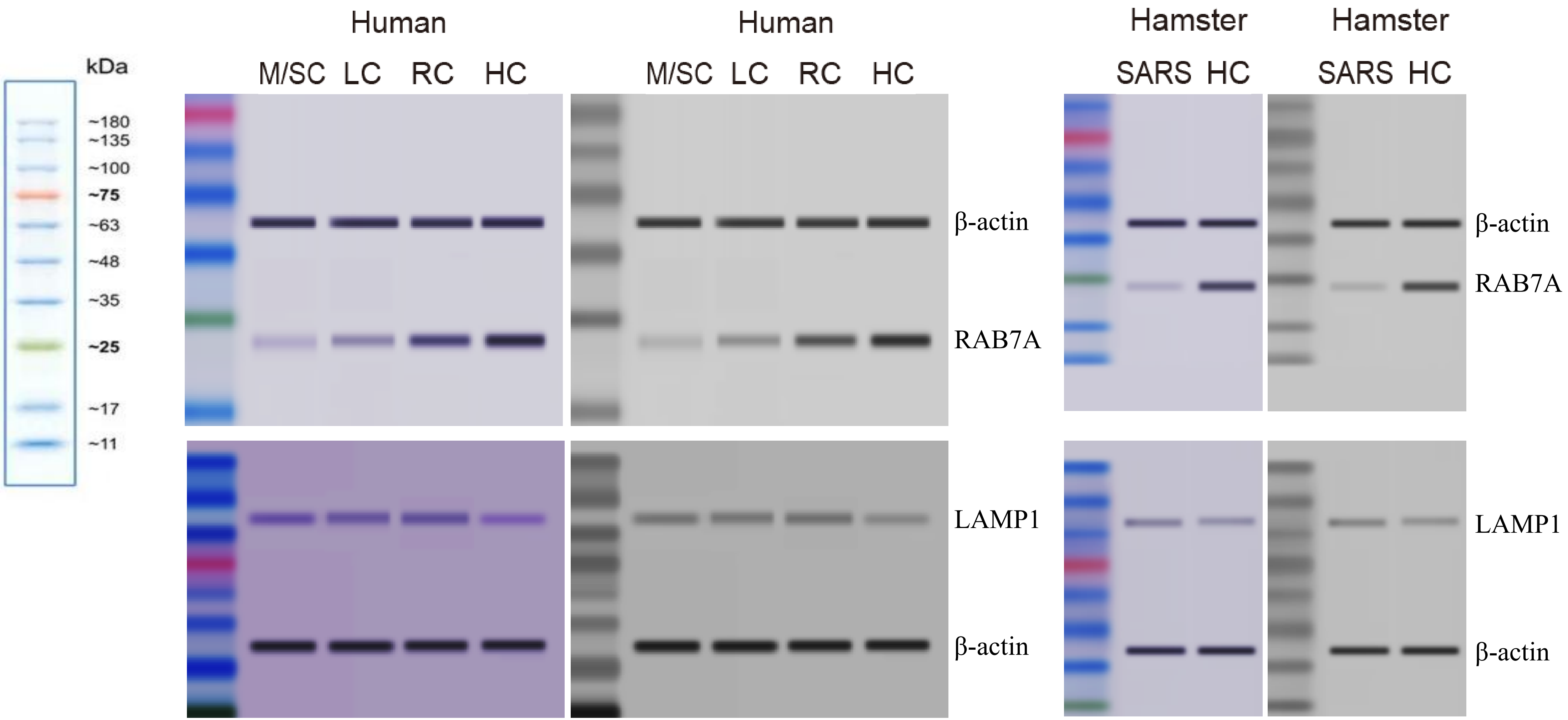

Fig. 3C

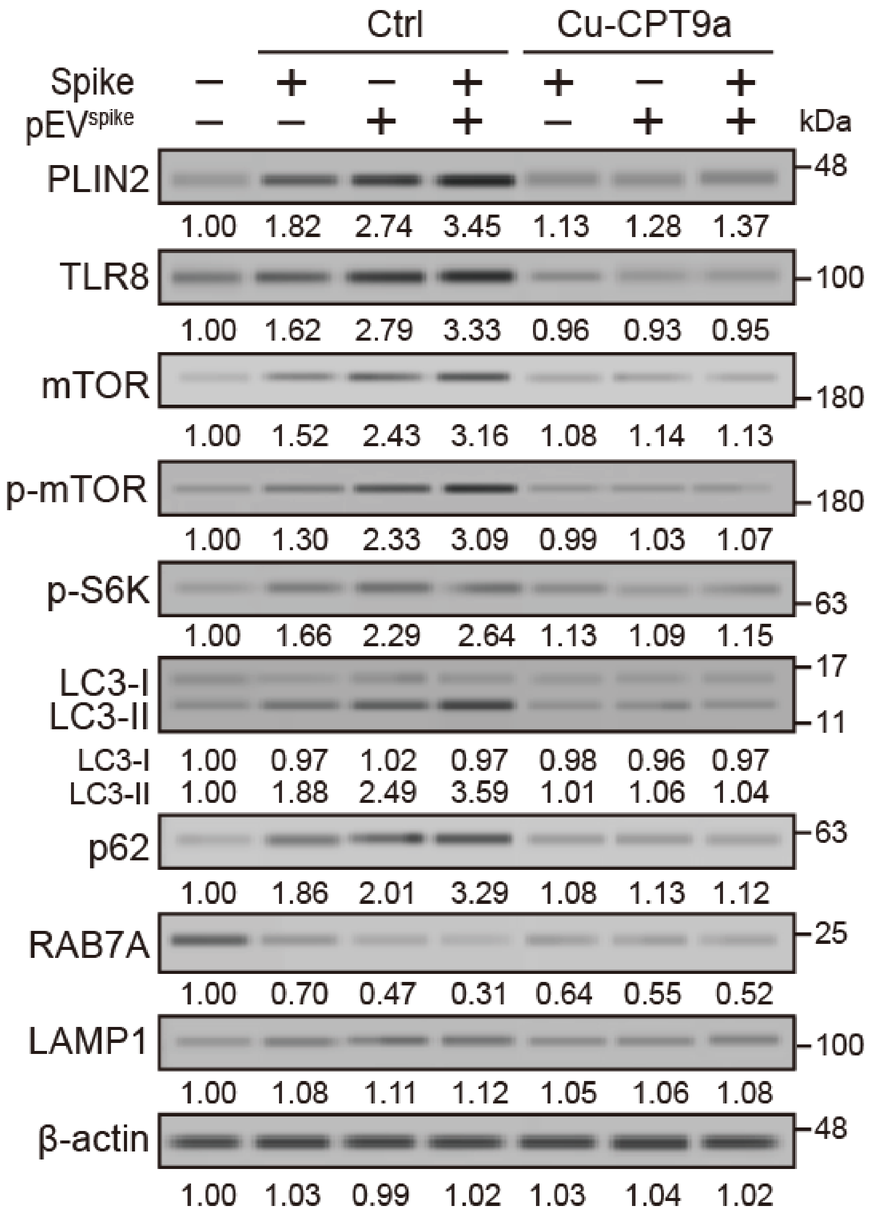

Ctrl
Cu-CPT9a
Spike − + − + + − +
pEVspike − − + + − + +
kDa
PLIN2
48
1.00 1.82 2.74 3.45 1.13 1.28 1.37
TLR8
100
1.00 1.62 2.79 3.33 0.96 0.93 0.95
mTOR
180
1.00 1.52 2.43 3.16 1.08 1.14 1.13
p-mTOR
180
1.00 1.30 2.33 3.09 0.99 1.03 1.07
p-S6K
63
1.00 1.66 2.29 2.64 1.13 1.09 1.15
LC3-I
LC3-II
17
11
LC3-I 1.00 0.97 1.02 0.97 0.98 0.96 0.97
LC3-II 1.00 1.88 2.49 3.59 1.01 1.06 1.04
p62
63
1.00 1.86 2.01 3.29 1.08 1.13 1.12
RAB7A
25
1.00 0.70 0.47 0.31 0.64 0.55 0.52
LAMP1
100
1.00 1.08 1.11 1.12 1.05 1.06 1.08
β-actin
48
1.00 1.03 0.99 1.02 1.03 1.04 1.02

PLIN2(48 kDa)/TLR8 (110 kDa)/mTOR (289 kDa)/p-mTOR (289 kDa)/p-S6K (70 kDa)

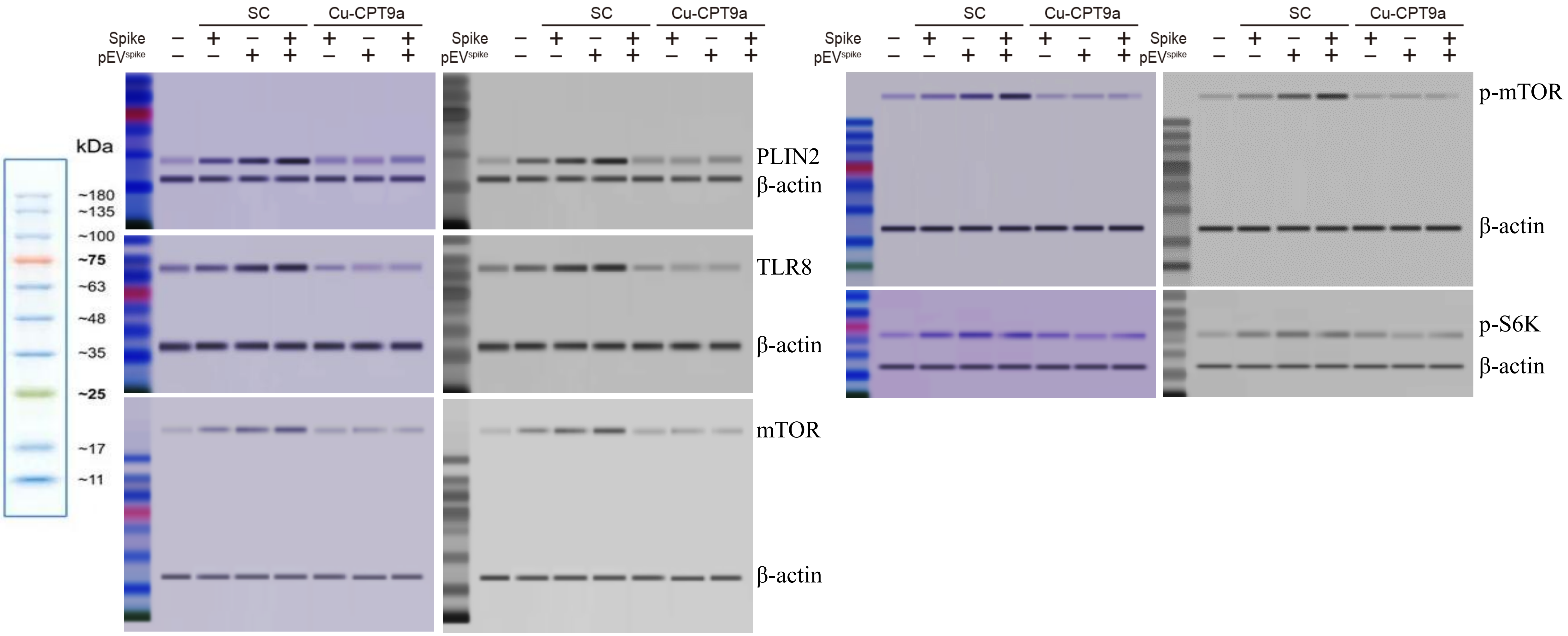

LC3 (14, 16kDa)/ p62 (62 kDa)/RAB7A (23 kDa)/LAMP1 (100~120 kDa)

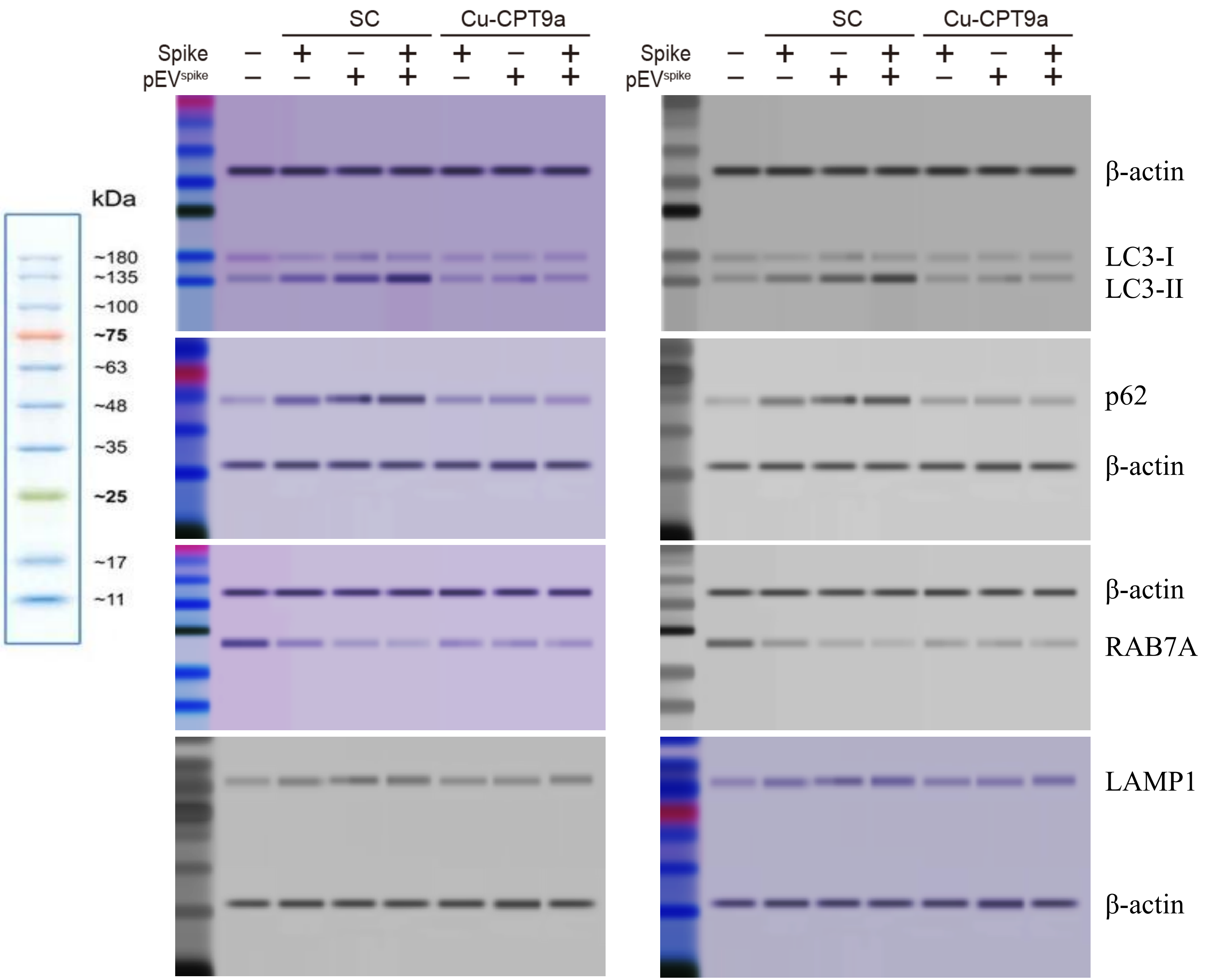

Fig. 3E

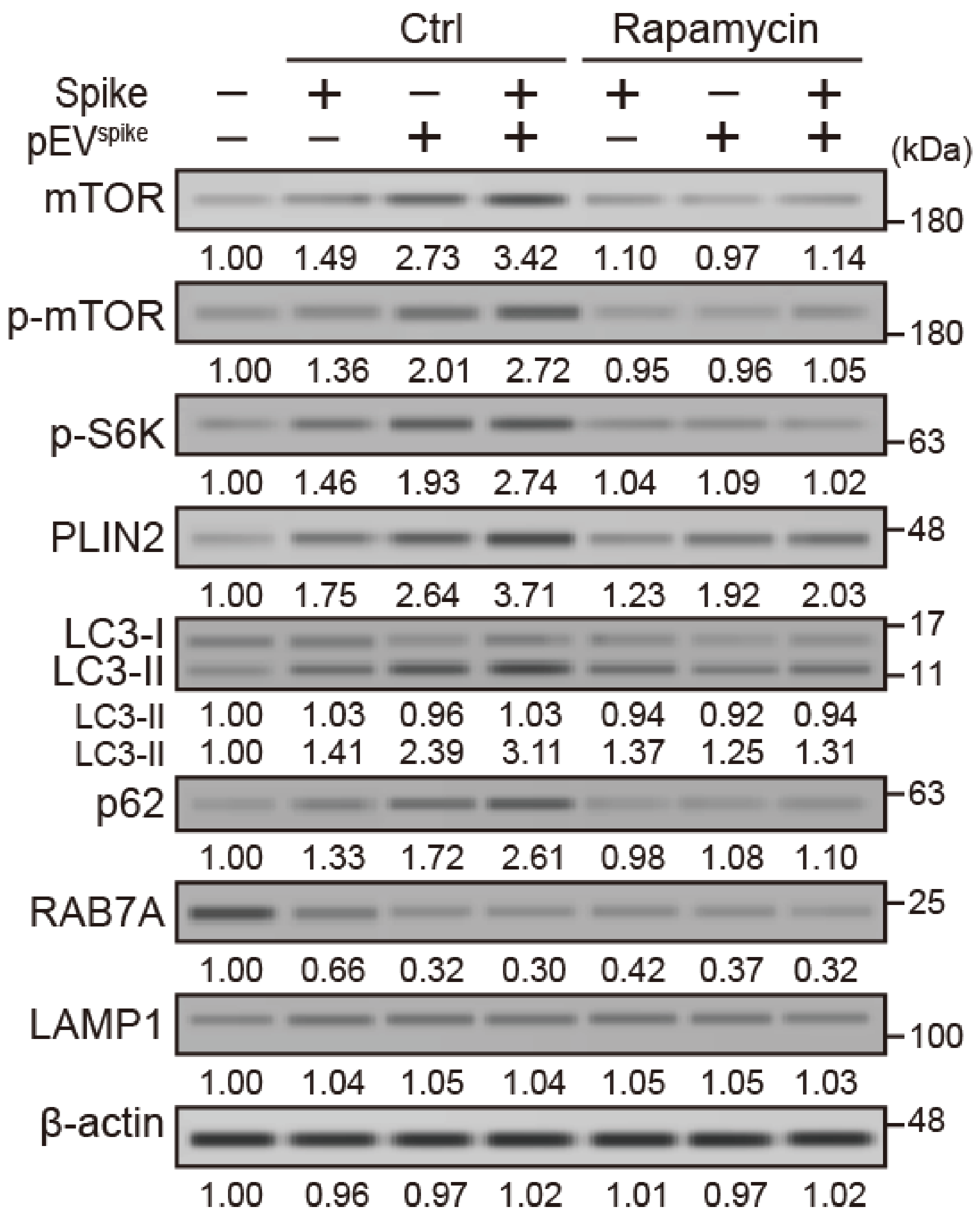
Ctrl
Rapamycin
Spike − + − + + − +
pEVspike − − + + − + +
(kDa)
mTOR
180
1.00 1.49 2.73 3.42 1.10 0.97 1.14
p-mTOR
180
1.00 1.36 2.01 2.72 0.95 0.96 1.05
p-S6K
63
1.00 1.46 1.93 2.74 1.04 1.09 1.02
PLIN2
48
1.00 1.75 2.64 3.71 1.23 1.92 2.03
LC3-I
LC3-II
17
11
LC3-II 1.00 1.03 0.96 1.03 0.94 0.92 0.94
LC3-II 1.00 1.41 2.39 3.11 1.37 1.25 1.31
p62
63
1.00 1.33 1.72 2.61 0.98 1.08 1.10
RAB7A
25
1.00 0.66 0.32 0.30 0.42 0.37 0.32
LAMP1
100
1.00 1.04 1.05 1.04 1.05 1.05 1.03
β-actin
48
1.00 0.96 0.97 1.02 1.01 0.97 1.02

mTOR (289 kDa)/p-mTOR (289 kDa)/p-S6K (70 kDa)/PLIN2(48 kDa)/LC3 (14, 16kDa)

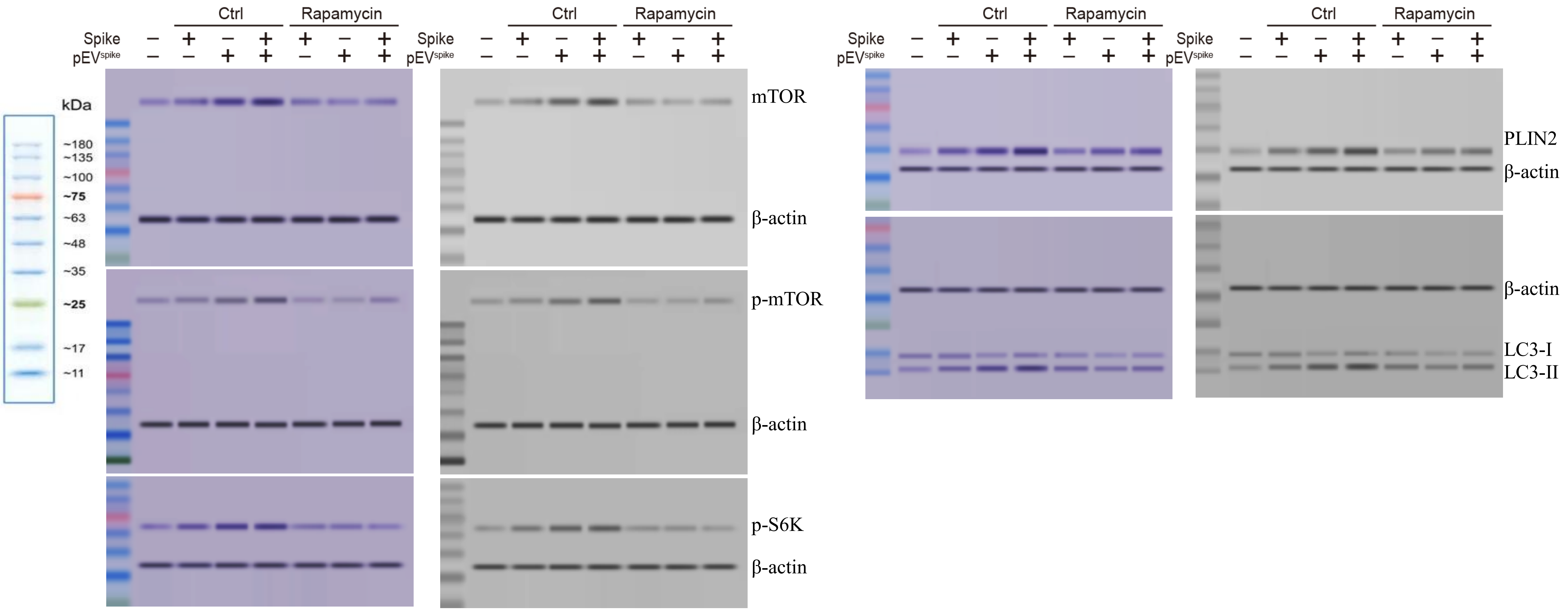

p62 (62 kDa)/RAB7A (23 kDa)/LAMP1 (100~120 kDa)

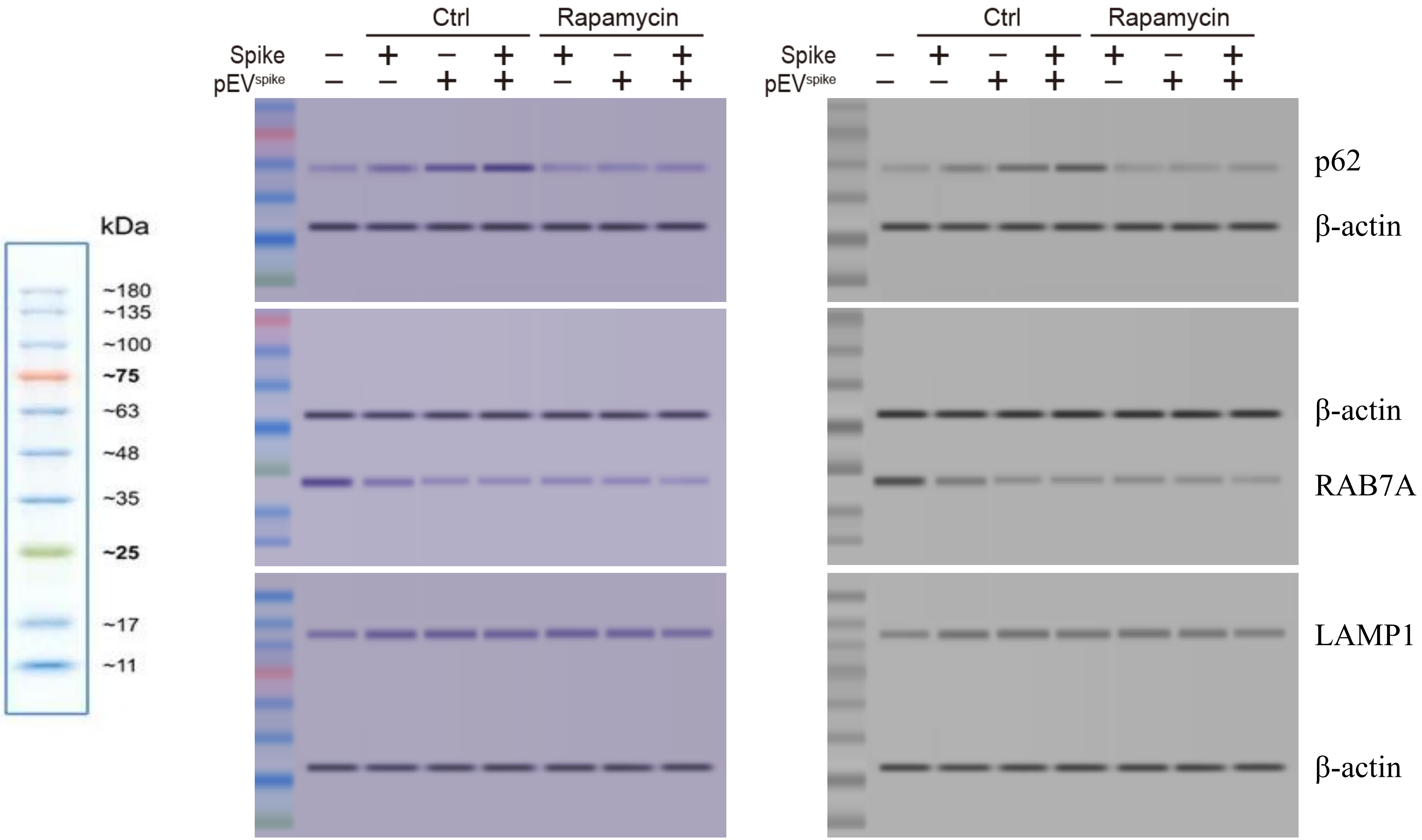

Fig. 4B

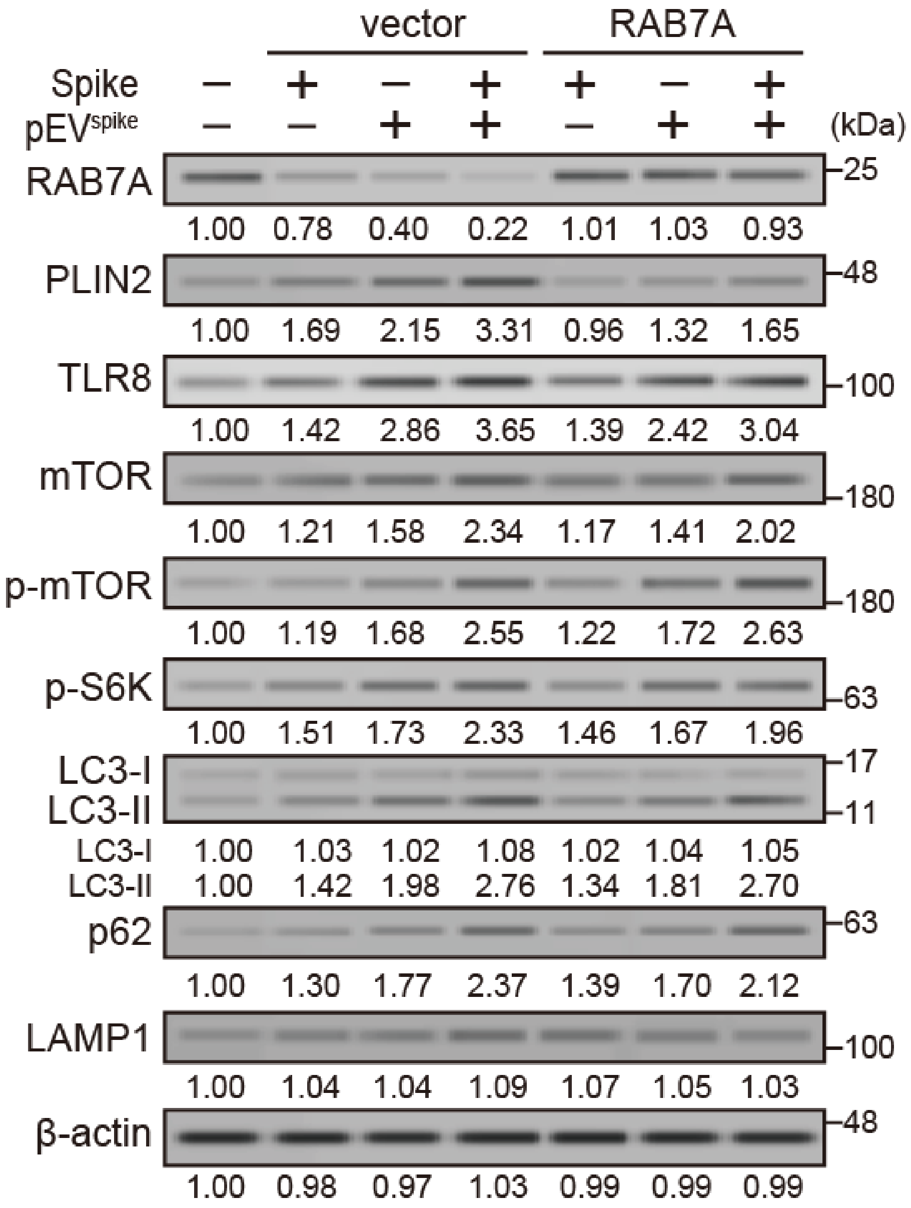

vector
RAB7A
Spike – + – + + – +
pEV^spike – – + + – + + (kDa)
RAB7A
1.00 0.78 0.40 0.22 1.01 1.03 0.93
25
PLIN2
1.00 1.69 2.15 3.31 0.96 1.32 1.65
48
TLR8
1.00 1.42 2.86 3.65 1.39 2.42 3.04
100
mTOR
1.00 1.21 1.58 2.34 1.17 1.41 2.02
180
p-mTOR
1.00 1.19 1.68 2.55 1.22 1.72 2.63
180
p-S6K
1.00 1.51 1.73 2.33 1.46 1.67 1.96
63
LC3-I
LC3-II
17
11
LC3-I 1.00 1.03 1.02 1.08 1.02 1.04 1.05
LC3-II 1.00 1.42 1.98 2.76 1.34 1.81 2.70
p62
63
1.00 1.30 1.77 2.37 1.39 1.70 2.12
LAMP1
100
1.00 1.04 1.04 1.09 1.07 1.05 1.03
β-actin
48
1.00 0.98 0.97 1.03 0.99 0.99 0.99

RAB7A (23 kDa)/PLIN2 (48 kDa)/TLR8 (110 kDa)/mTOR (289 kDa)/p-mTOR (289 kDa)/p-S6K (70 kDa)

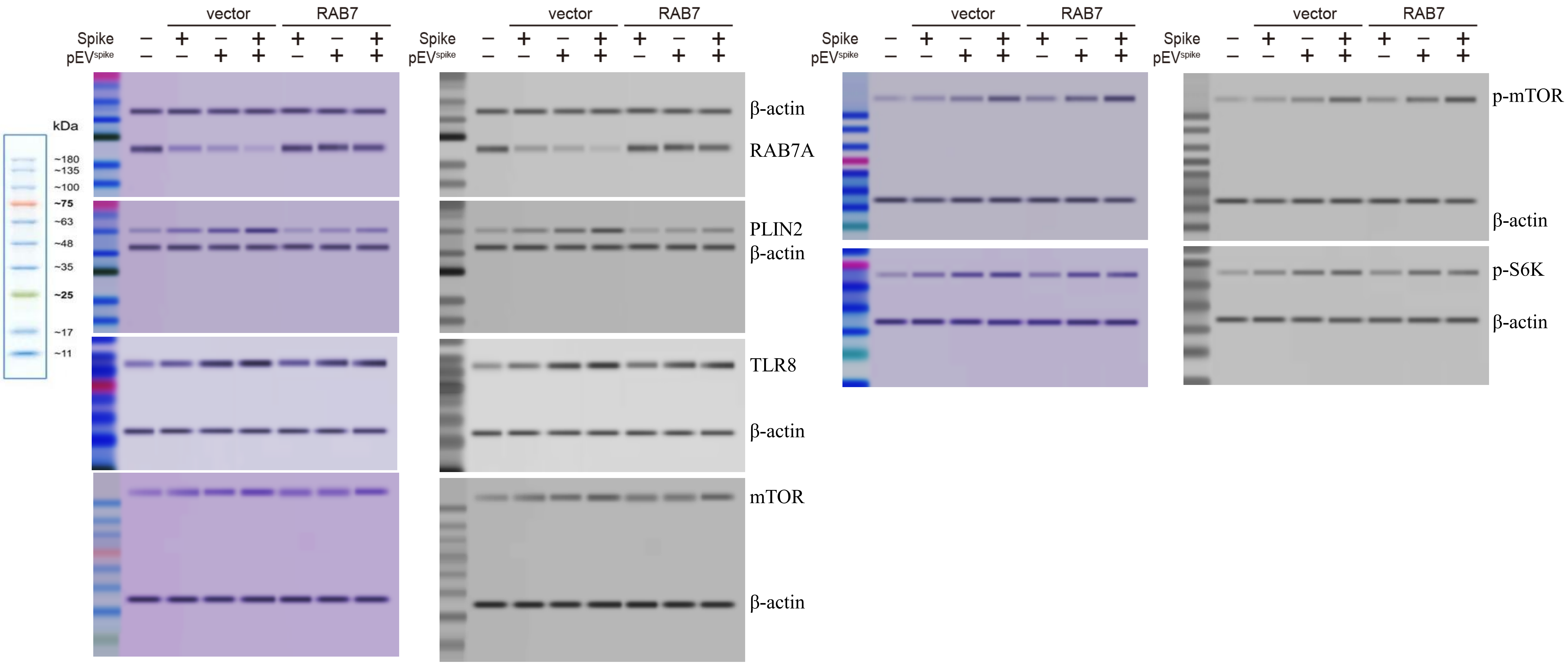

LC3 (14, 16kDa)/p62 (62 kDa)/LAMP1 (100~120 kDa)

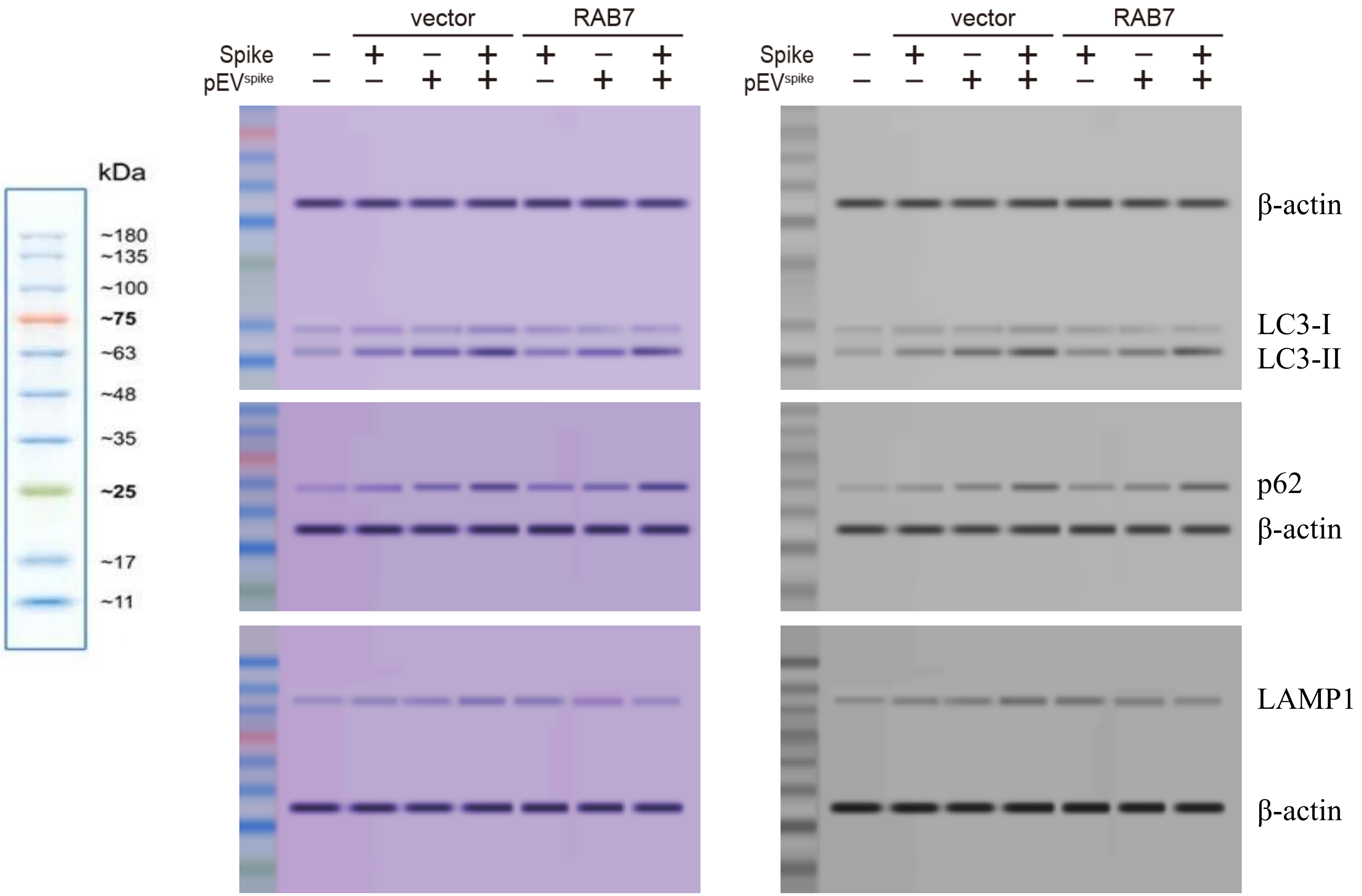

Fig. 5H

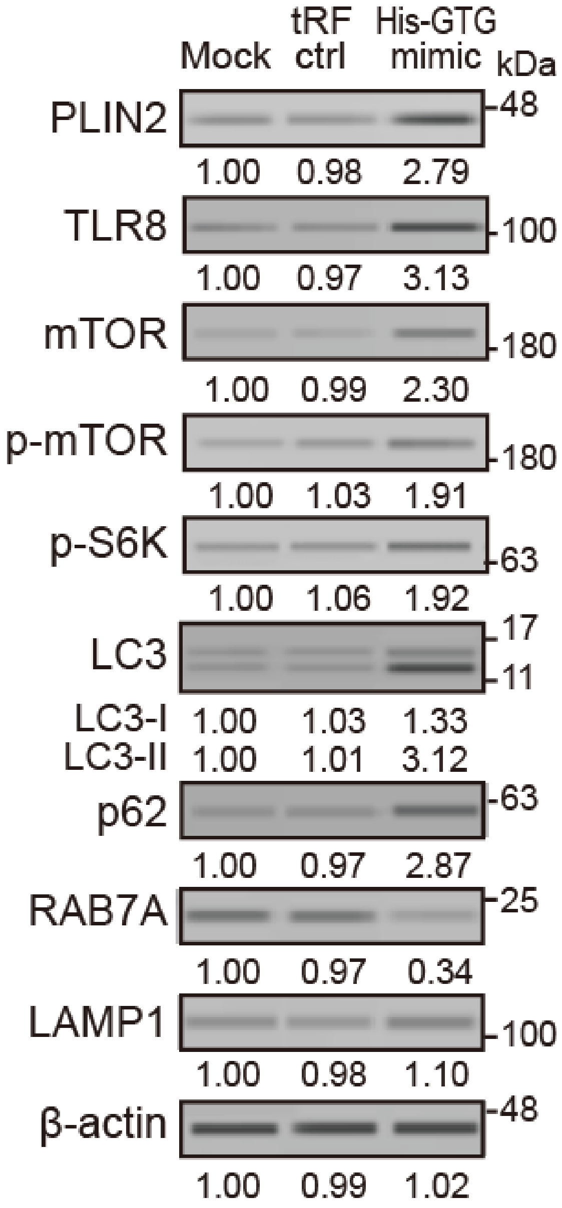

Mock
tRF ctrl
His-GTG mimic
kDa
PLIN2
48
1.00 0.98 2.79
TLR8
100
1.00 0.97 3.13
mTOR
180
1.00 0.99 2.30
p-mTOR
180
1.00 1.03 1.91
p-S6K
63
1.00 1.06 1.92
LC3
17
11
LC3-I 1.00 1.03 1.33
LC3-II 1.00 1.01 3.12
p62
63
1.00 0.97 2.87
RAB7A
25
1.00 0.97 0.34
LAMP1
100
1.00 0.98 1.10
β-actin
48
1.00 0.99 1.02

PLIN2(48 kDa)/TLR8 (110 kDa)/mTOR (289 kDa)/p-mTOR (289 kDa)/p-S6K (70 kDa)

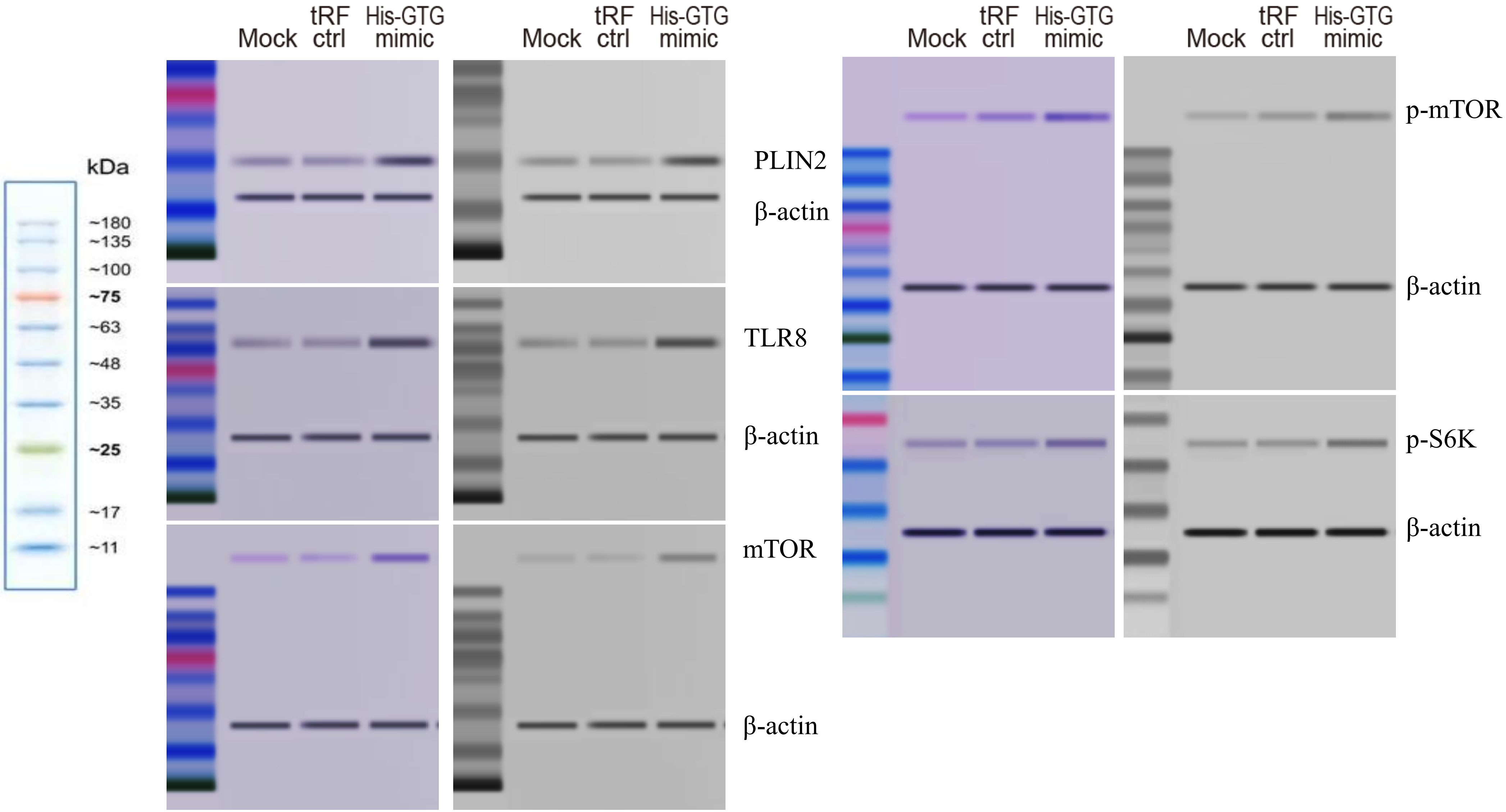

LC3 (14, 16kDa)/p62 (62 kDa)/RAB7A (23 kDa)/LAMP1 (100~120 kDa)

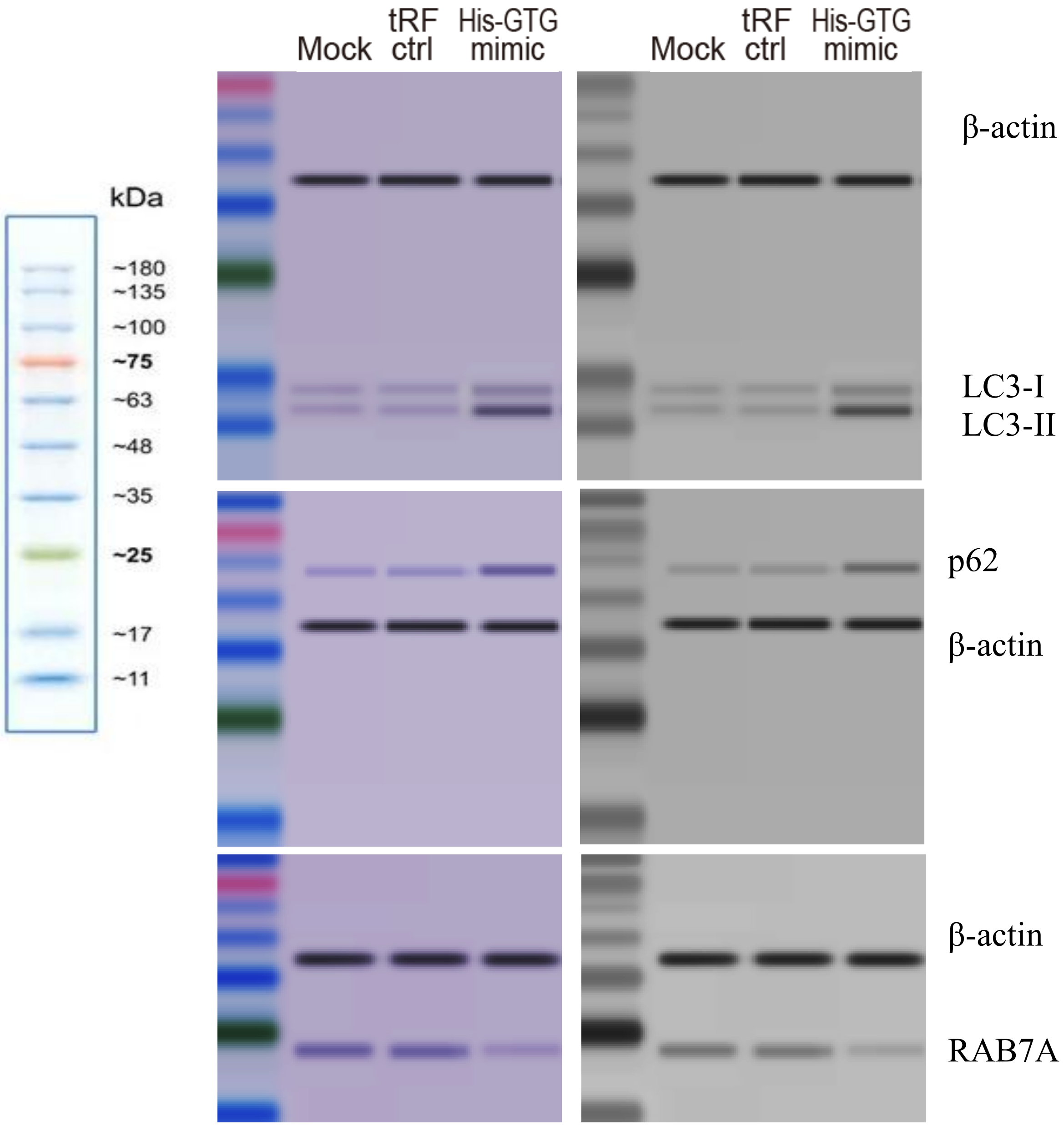

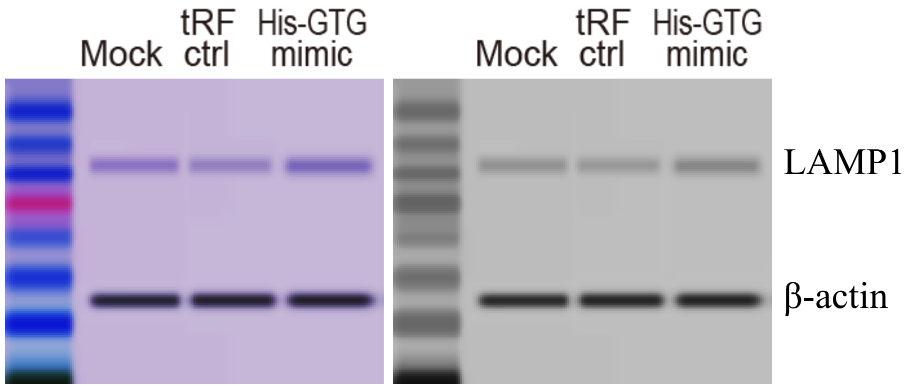

Fig. 6B

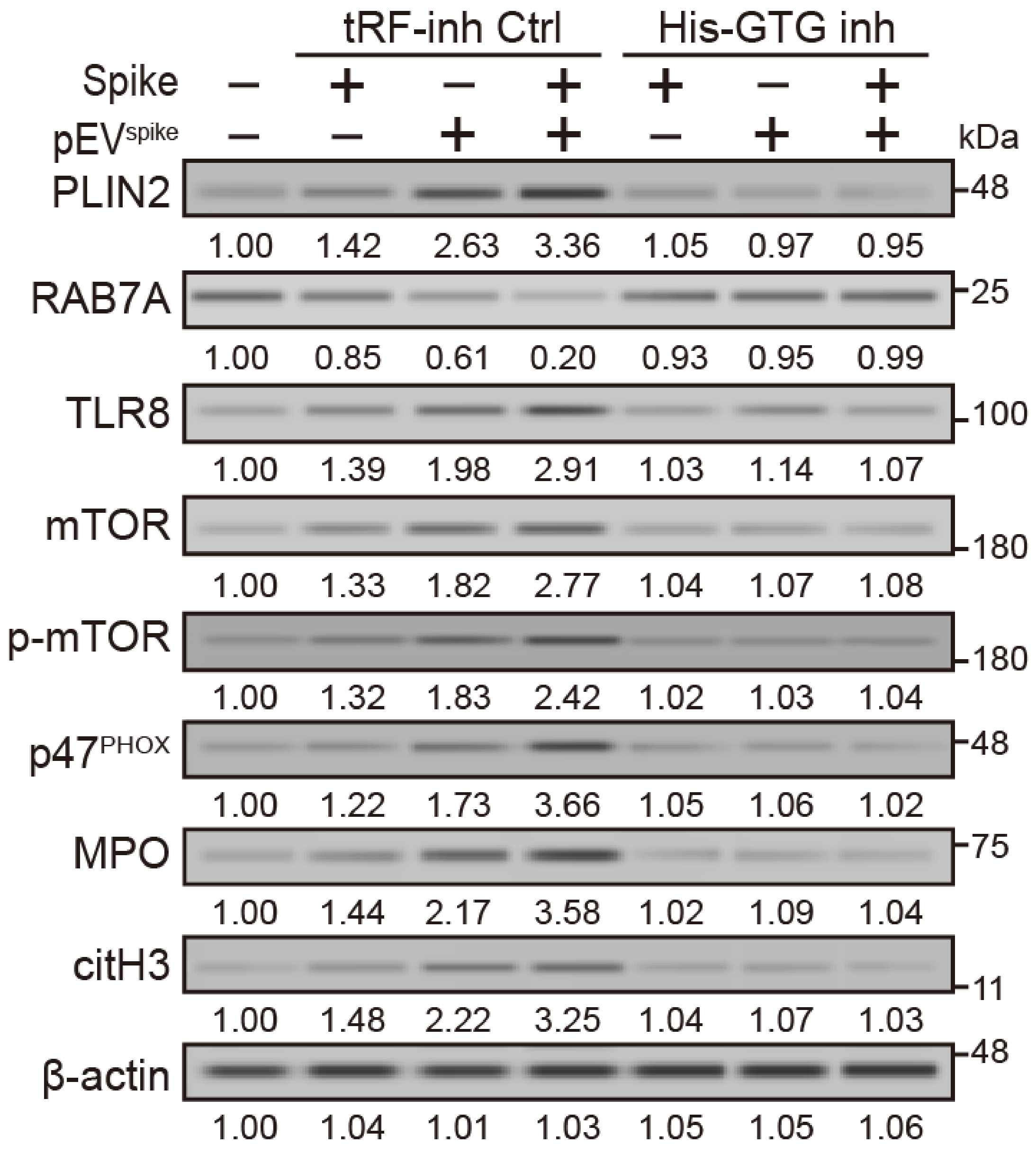
tRF-inh Ctrl
His-GTG inh
Spike – + – + + – +
pEVspike – – + + – + +
kDa
PLIN2
48
1.00 1.42 2.63 3.36 1.05 0.97 0.95
RAB7A
25
1.00 0.85 0.61 0.20 0.93 0.95 0.99
TLR8
100
1.00 1.39 1.98 2.91 1.03 1.14 1.07
mTOR
180
1.00 1.33 1.82 2.77 1.04 1.07 1.08
p-mTOR
180
1.00 1.32 1.83 2.42 1.02 1.03 1.04
p47PHOX
48
1.00 1.22 1.73 3.66 1.05 1.06 1.02
MPO
75
1.00 1.44 2.17 3.58 1.02 1.09 1.04
citH3
11
1.00 1.48 2.22 3.25 1.04 1.07 1.03
β-actin
48
1.00 1.04 1.01 1.03 1.05 1.05 1.06

PLIN2(48 kDa)/RAB7A (23 kDa)

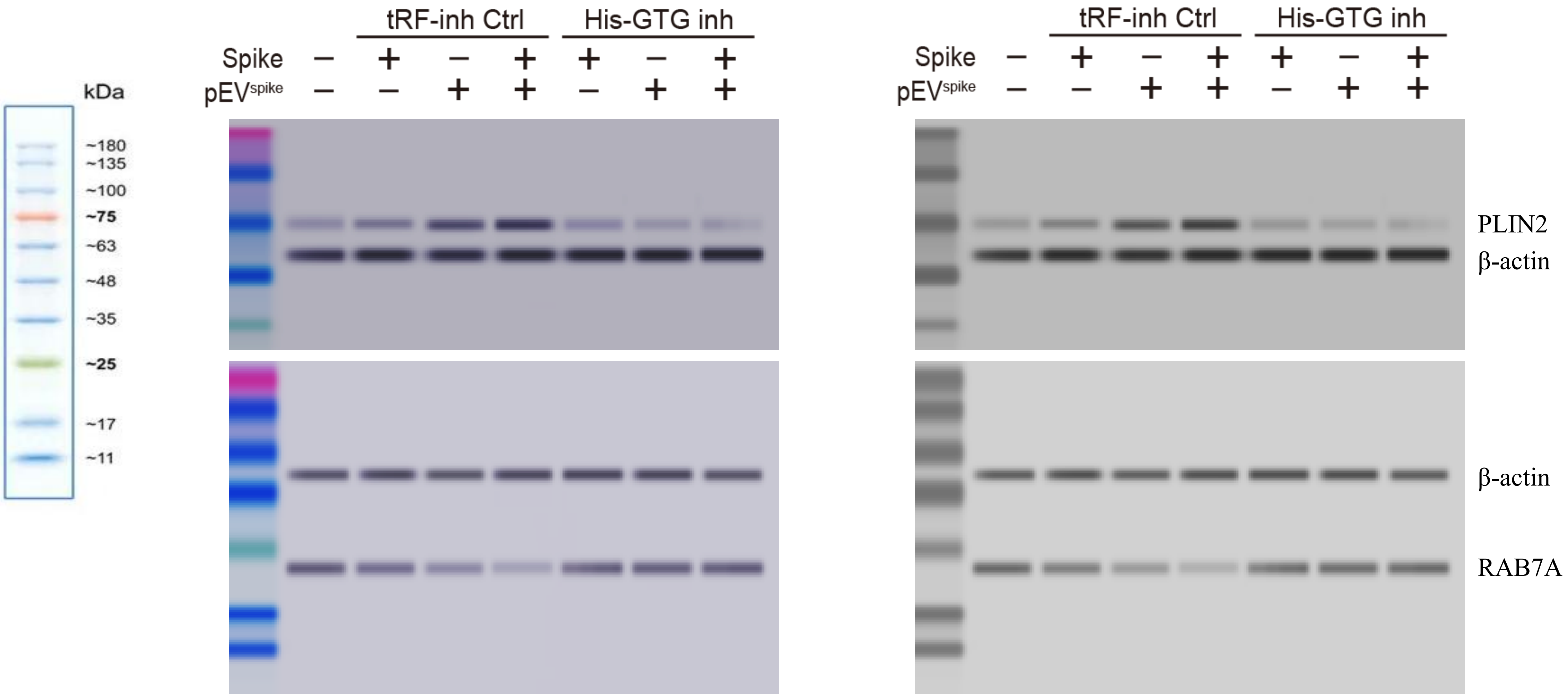

TLR8 (110 kDa)/mTOR (289 kDa)/p-mTOR (289 kDa)

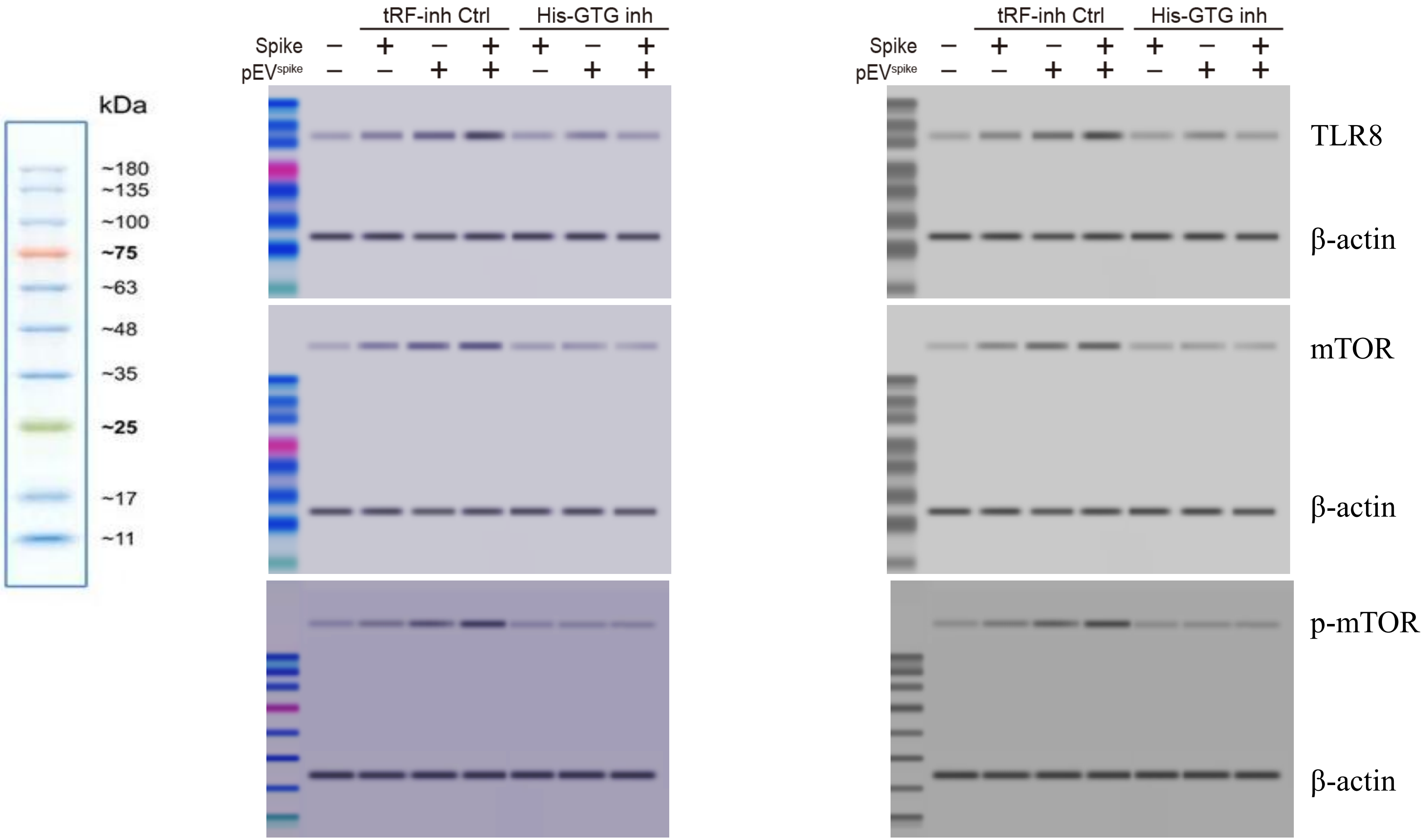

p47^{PHOX} (47 kDa)/MPO (62kDa)/ citH3 (14 kDa)

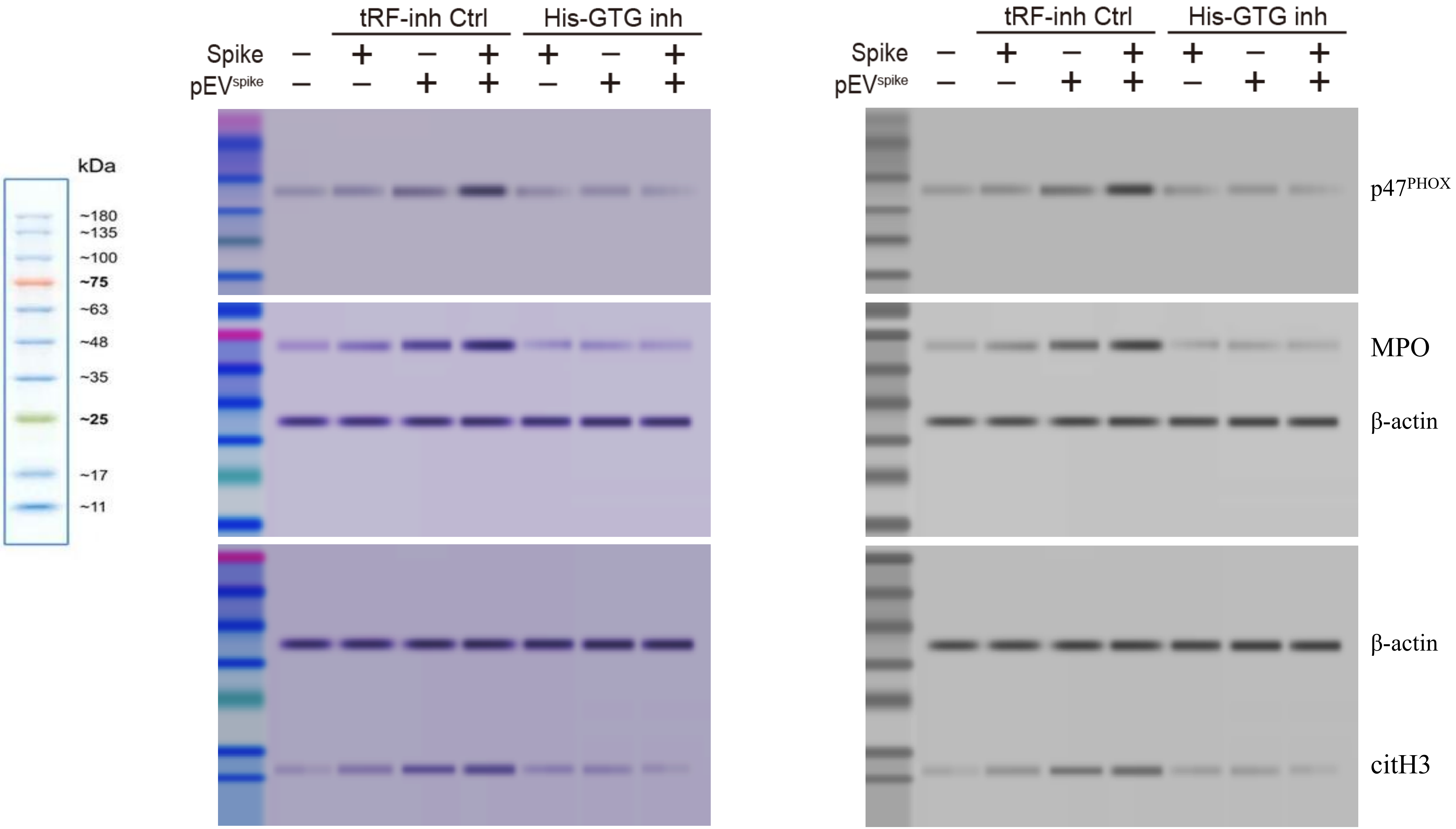

Fig. S2G

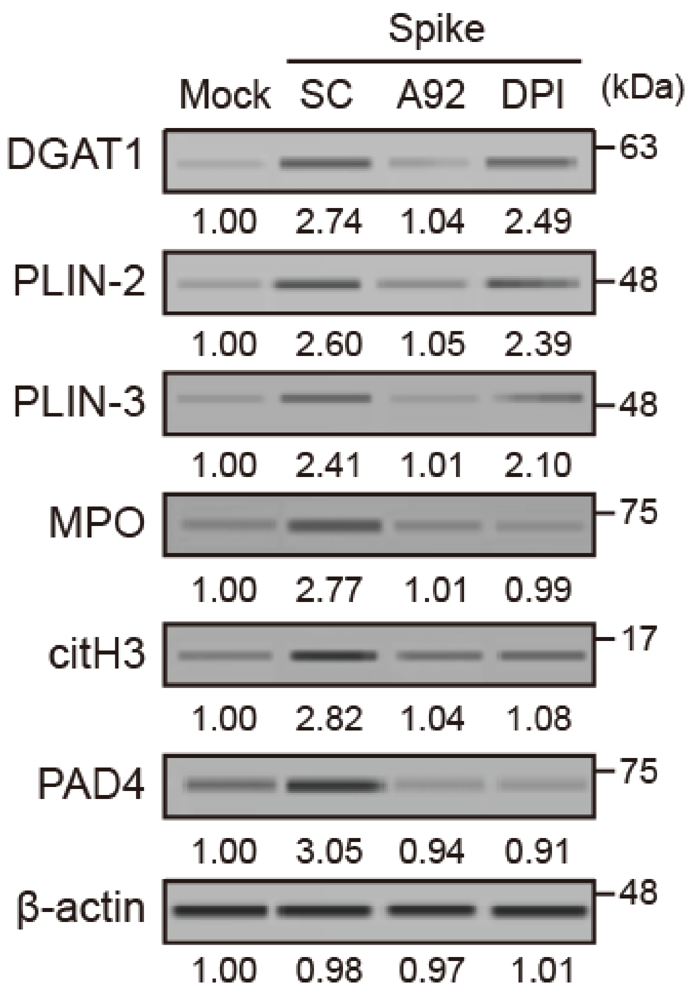

Spike
Mock SC A92 DPI (kDa)
DGAT1
63
1.00 2.74 1.04 2.49
PLIN-2
48
1.00 2.60 1.05 2.39
PLIN-3
48
1.00 2.41 1.01 2.10
MPO
75
1.00 2.77 1.01 0.99
citH3
17
1.00 2.82 1.04 1.08
PAD4
75
1.00 3.05 0.94 0.91
β-actin
48
1.00 0.98 0.97 1.01

DGAT1 (55 kDa)/ PLIN2 (48 kDa)/PLIN3 (47 kDa)/MPO (72 kDa)/citH3 (14 kDa)/PAD4 (72 kDa)

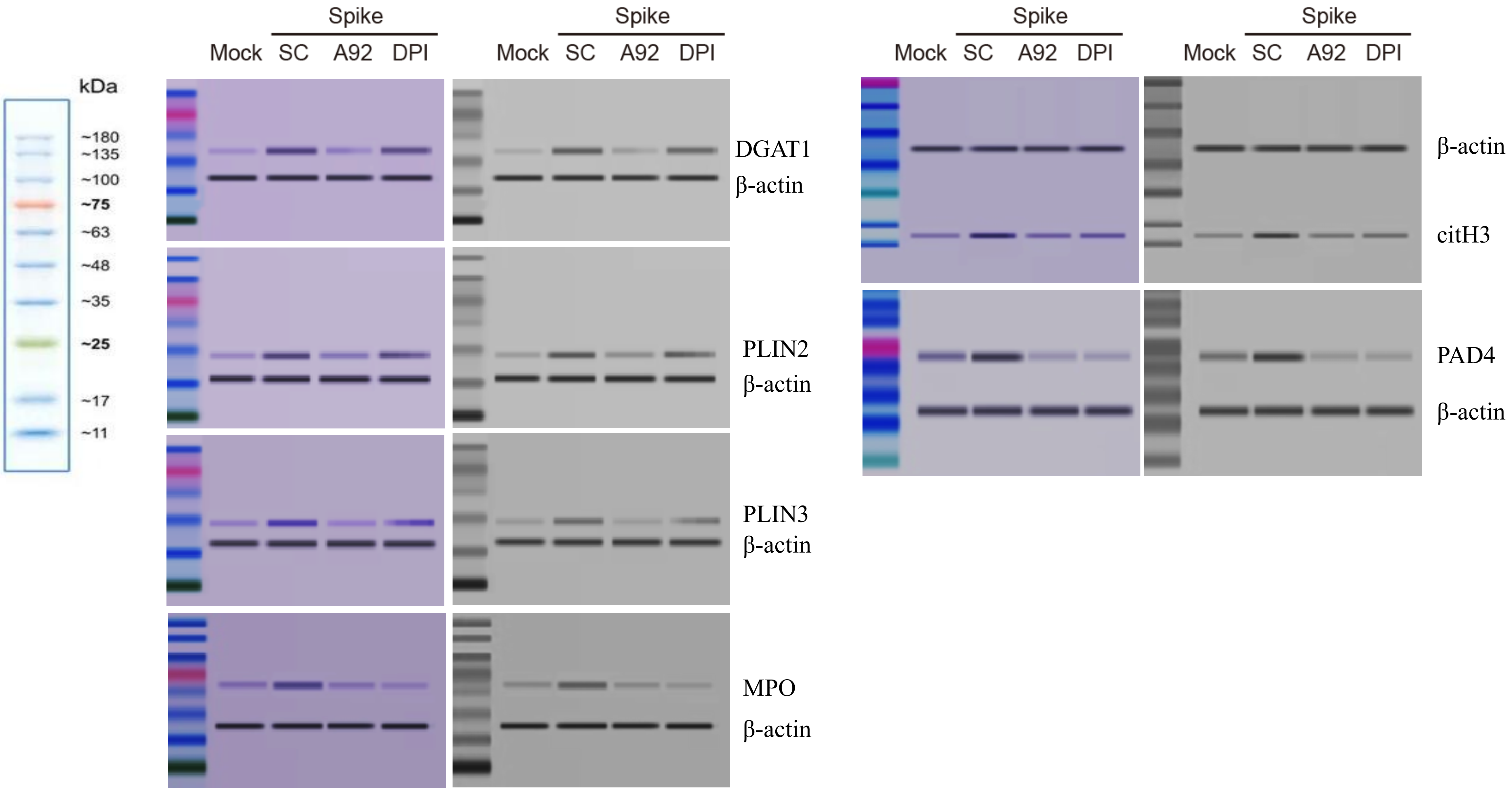

Fig. S6A

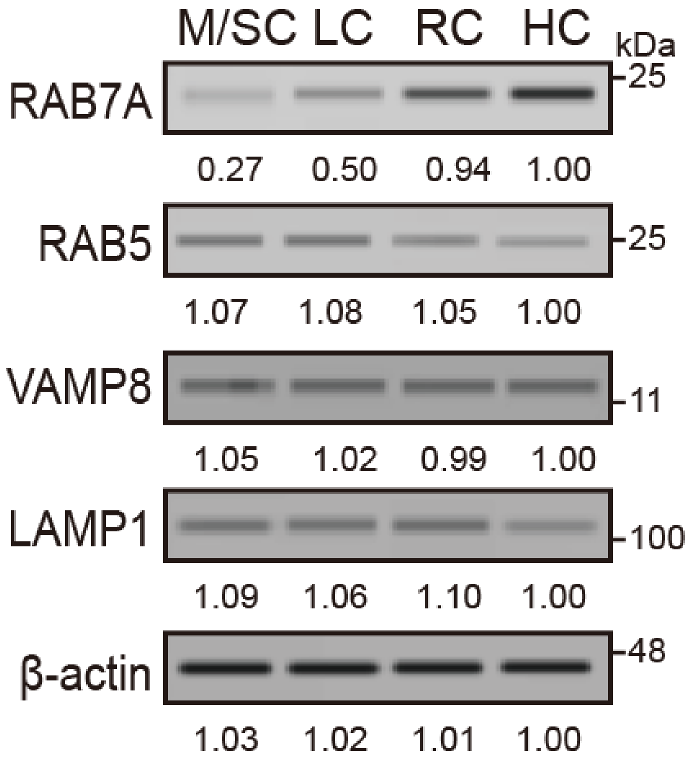

M/SC LC RC HC
kDa
RAB7A
25
0.27 0.50 0.94 1.00
RAB5
25
1.07 1.08 1.05 1.00
VAMP8
11
1.05 1.02 0.99 1.00
LAMP1
100
1.09 1.06 1.10 1.00
β-actin
48
1.03 1.02 1.01 1.00

RAB7A (23 kDa)/RAB5 (25 kDa)/VAMP8 (15 kDa)/LAMP1 (100-120 kDa)

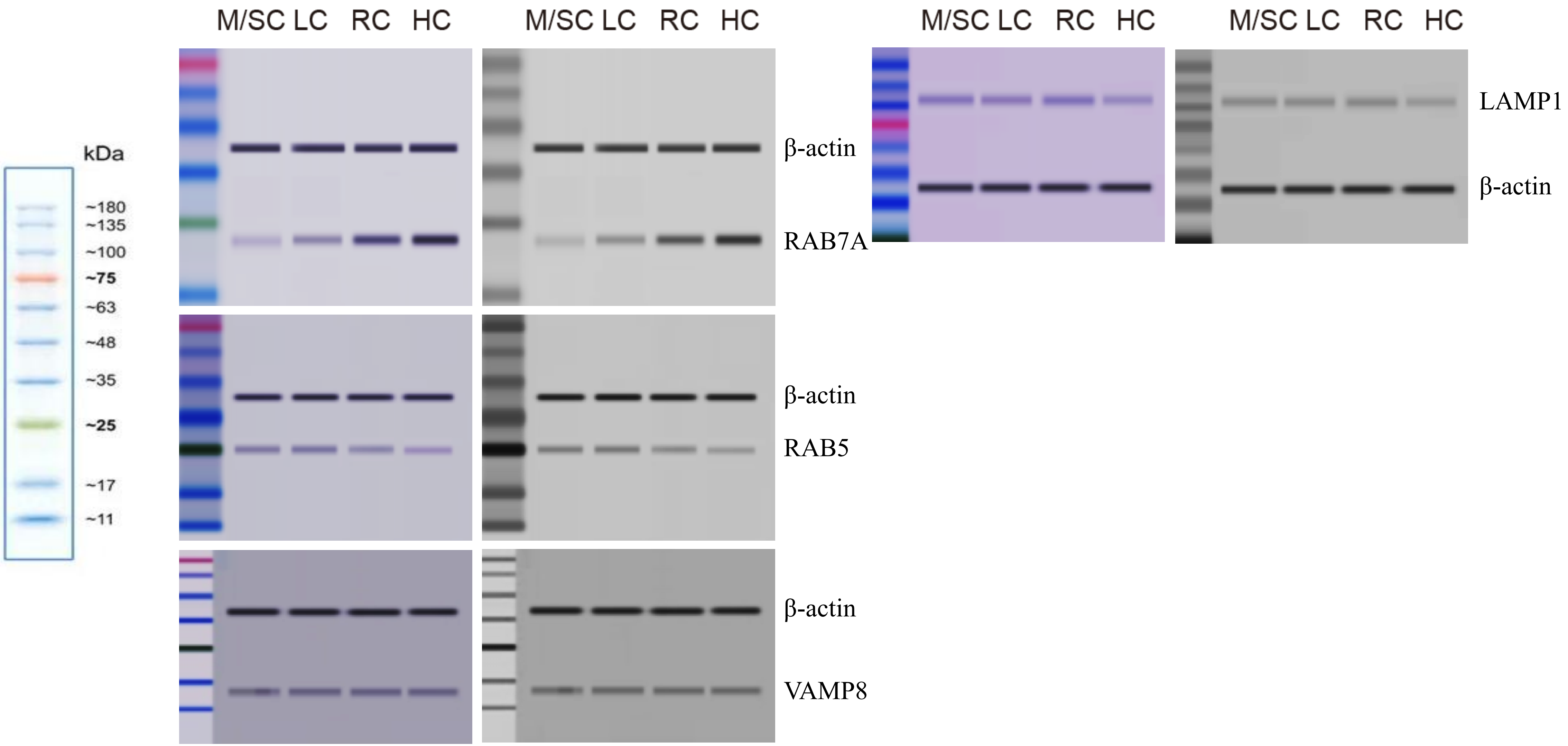

Fig. S6B

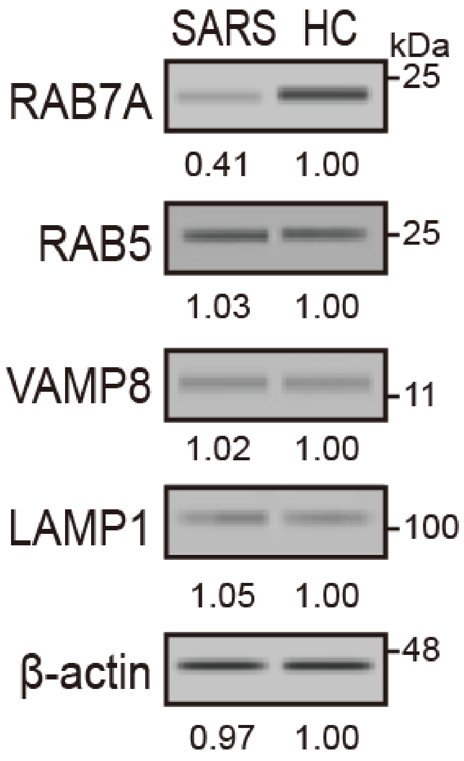

SARS
HC
kDa
RAB7A
25
0.41
1.00
RAB5
25
1.03
1.00
VAMP8
11
1.02
1.00
LAMP1
100
1.05
1.00
β-actin
48
0.97
1.00

RAB7A (23 kDa)/RAB5 (25 kDa)/VAMP8 (15 kDa)/LAMP1 (100-120 kDa)

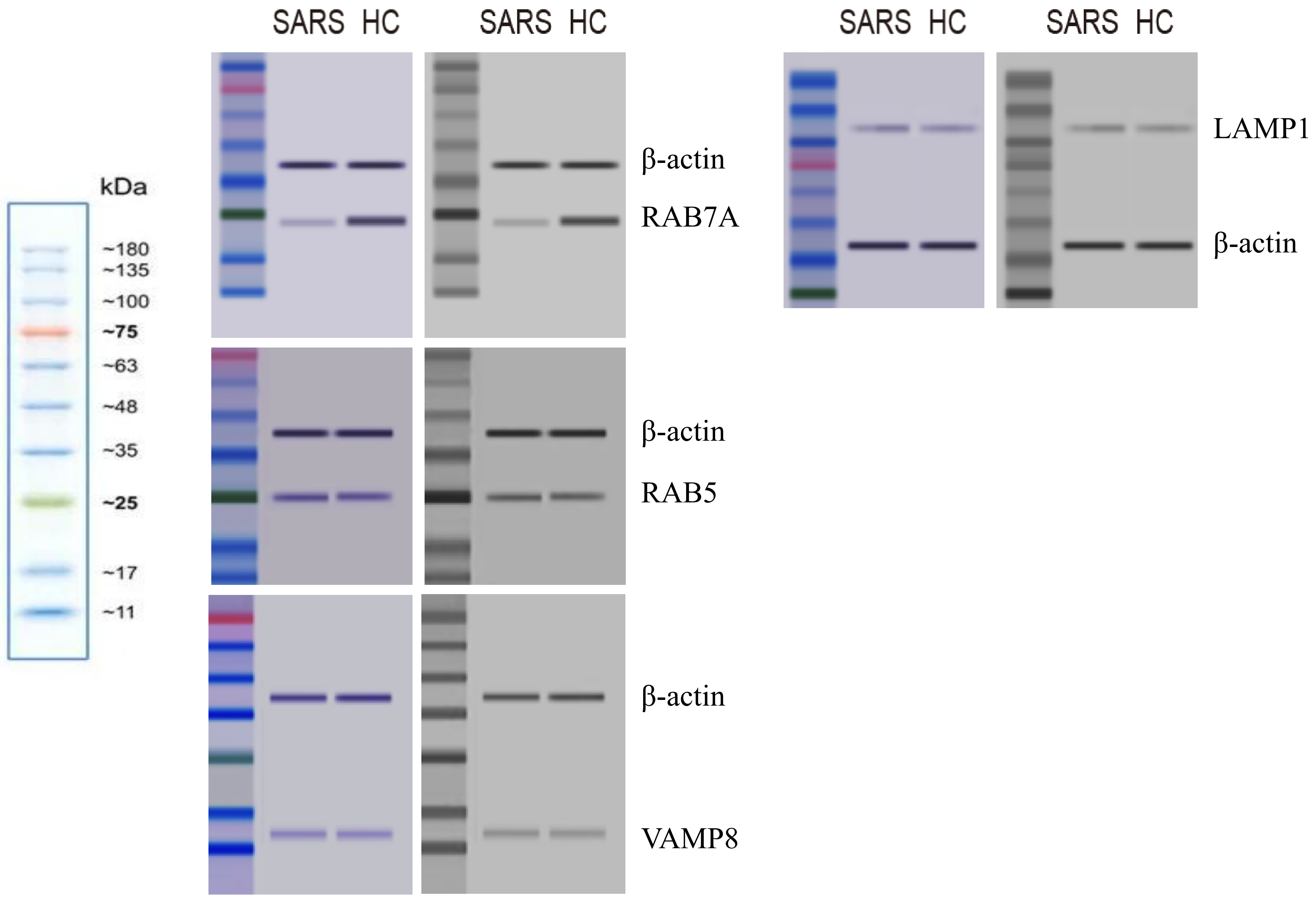

Fig. S6C

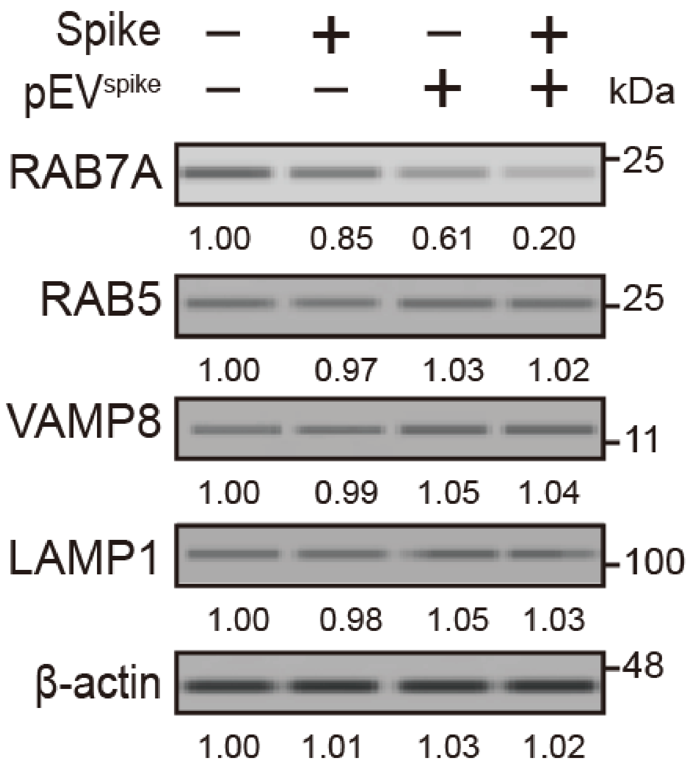

Spike – + – +
pEVspike – – + +
kDa
RAB7A
25
1.00 0.85 0.61 0.20
RAB5
25
1.00 0.97 1.03 1.02
VAMP8
11
1.00 0.99 1.05 1.04
LAMP1
100
1.00 0.98 1.05 1.03
β-actin
48
1.00 1.01 1.03 1.02

RAB7A (23 kDa)/RAB5 (25 kDa)/VAMP8 (15 kDa)/LAMP1 (100-120 kDa)

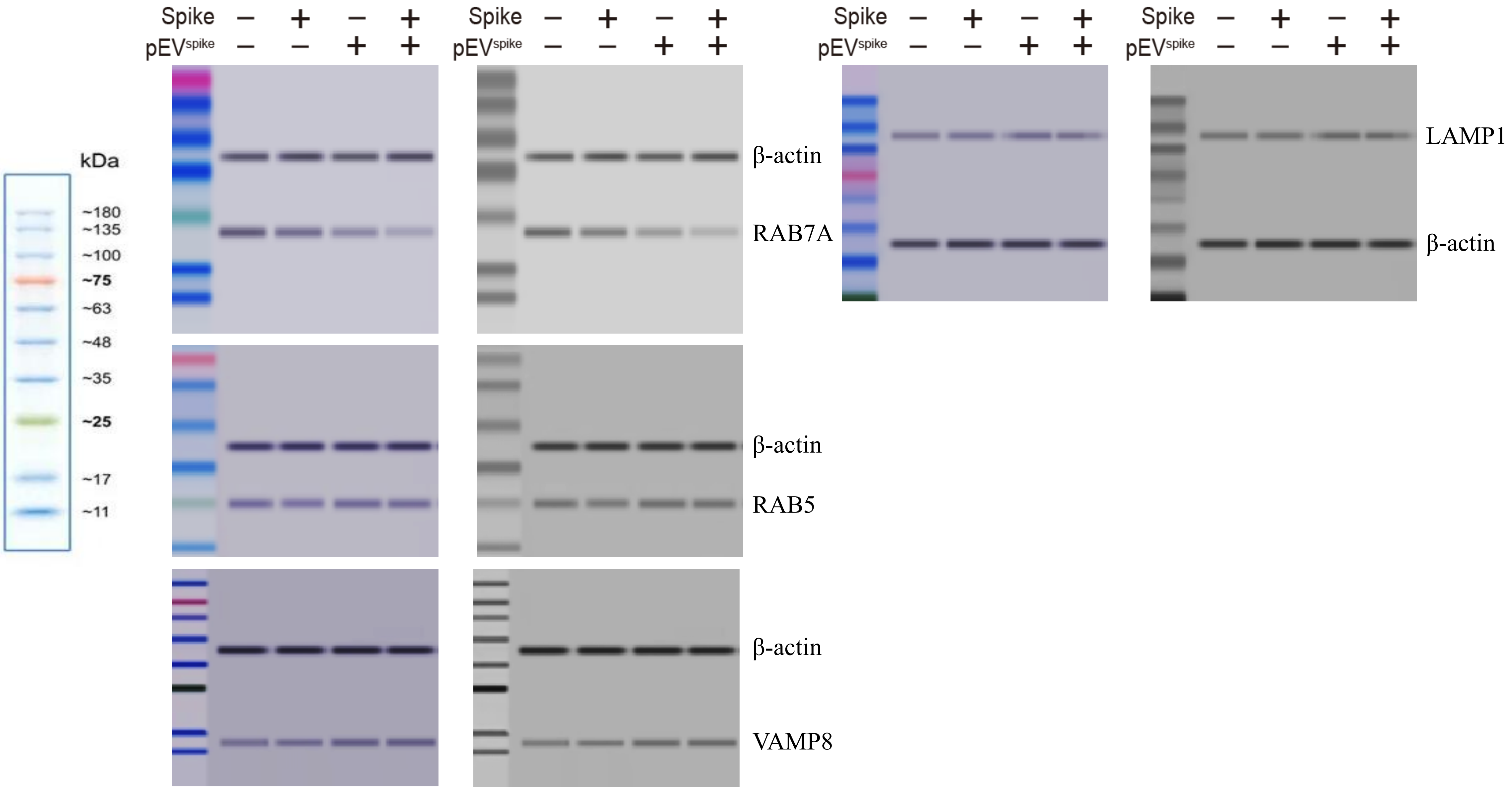

Fig. S8E

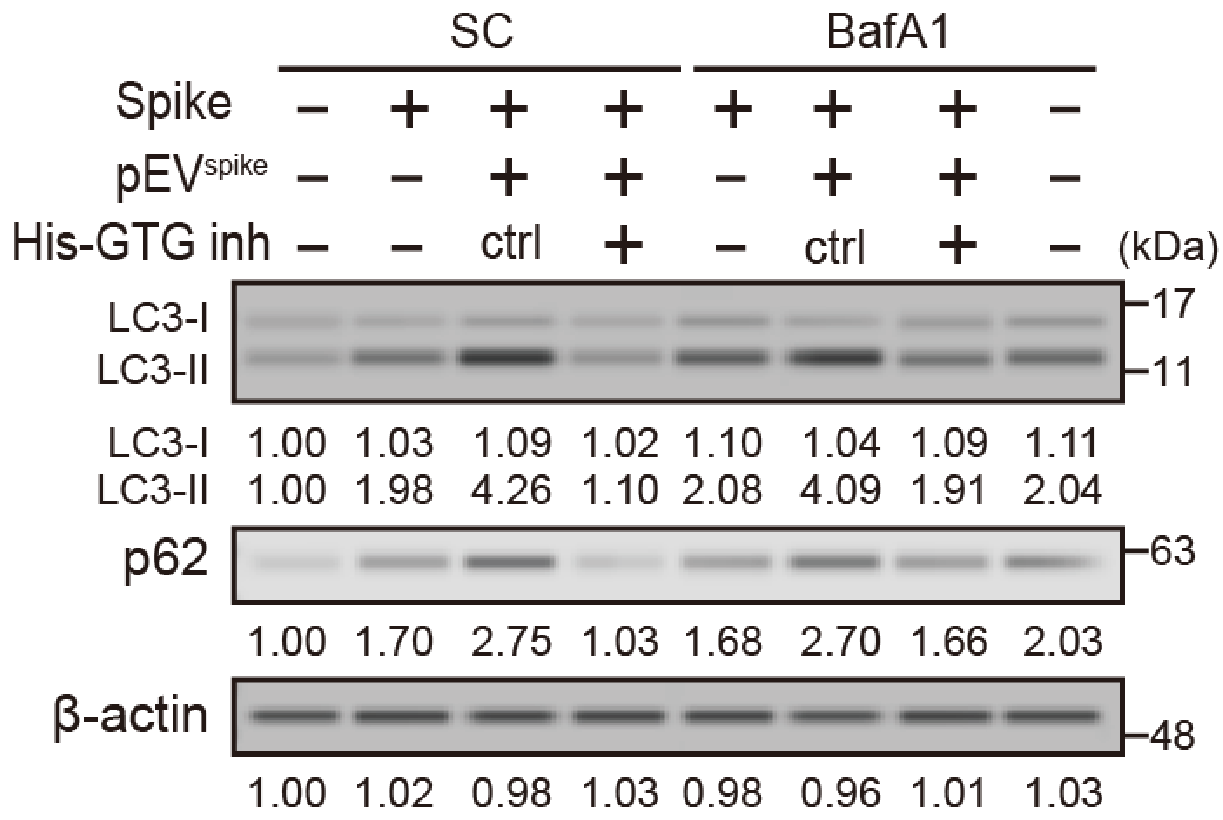
SC
BafA1
Spike – + + + + + + –
pEVspike – – + + – + + –
His-GTG inh – – ctrl + – ctrl + –
(kDa)
LC3-I
LC3-II
17
11
LC3-I 1.00 1.03 1.09 1.02 1.10 1.04 1.09 1.11
LC3-II 1.00 1.98 4.26 1.10 2.08 4.09 1.91 2.04
p62
63
1.00 1.70 2.75 1.03 1.68 2.70 1.66 2.03
β-actin
48
1.00 1.02 0.98 1.03 0.98 0.96 1.01 1.03

LC3 (14, 16 kDa)/p62 (62 kDa)

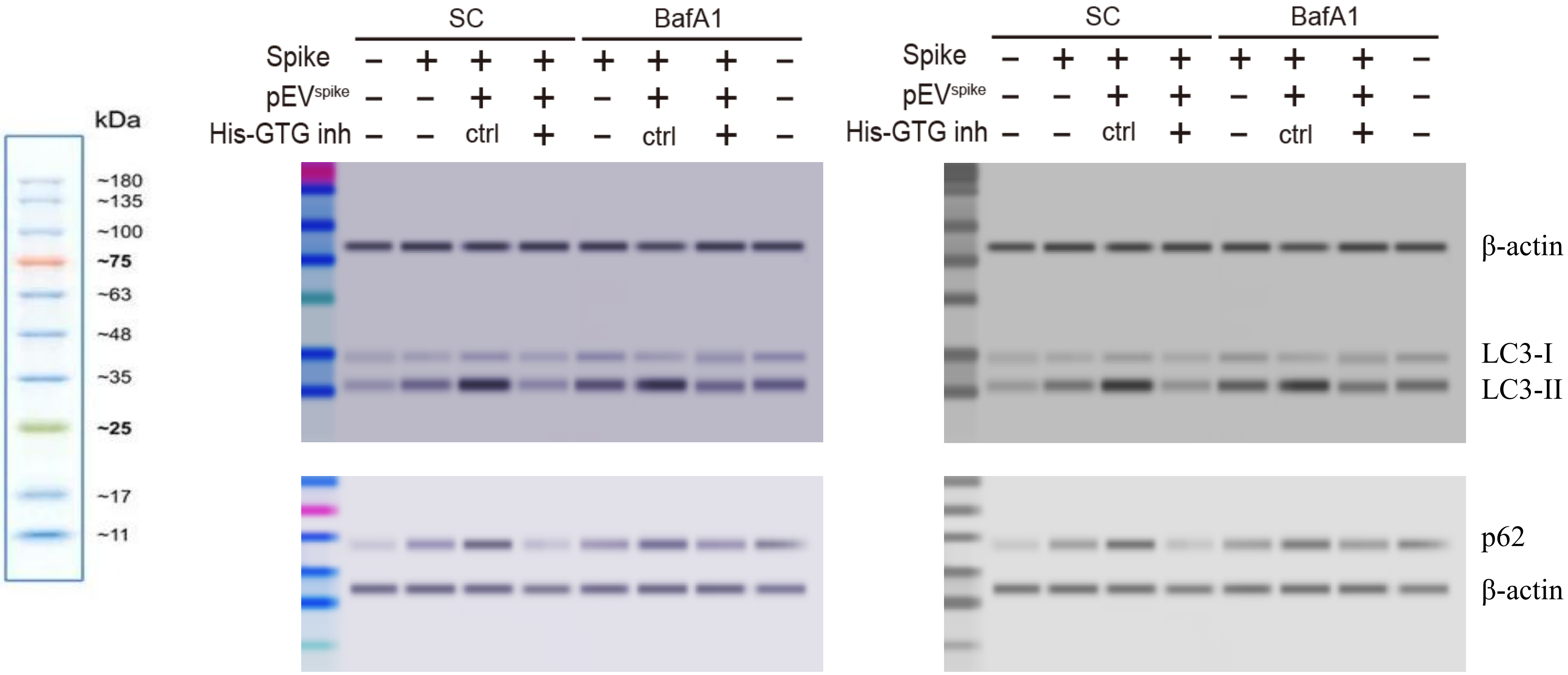

Fig. S9D

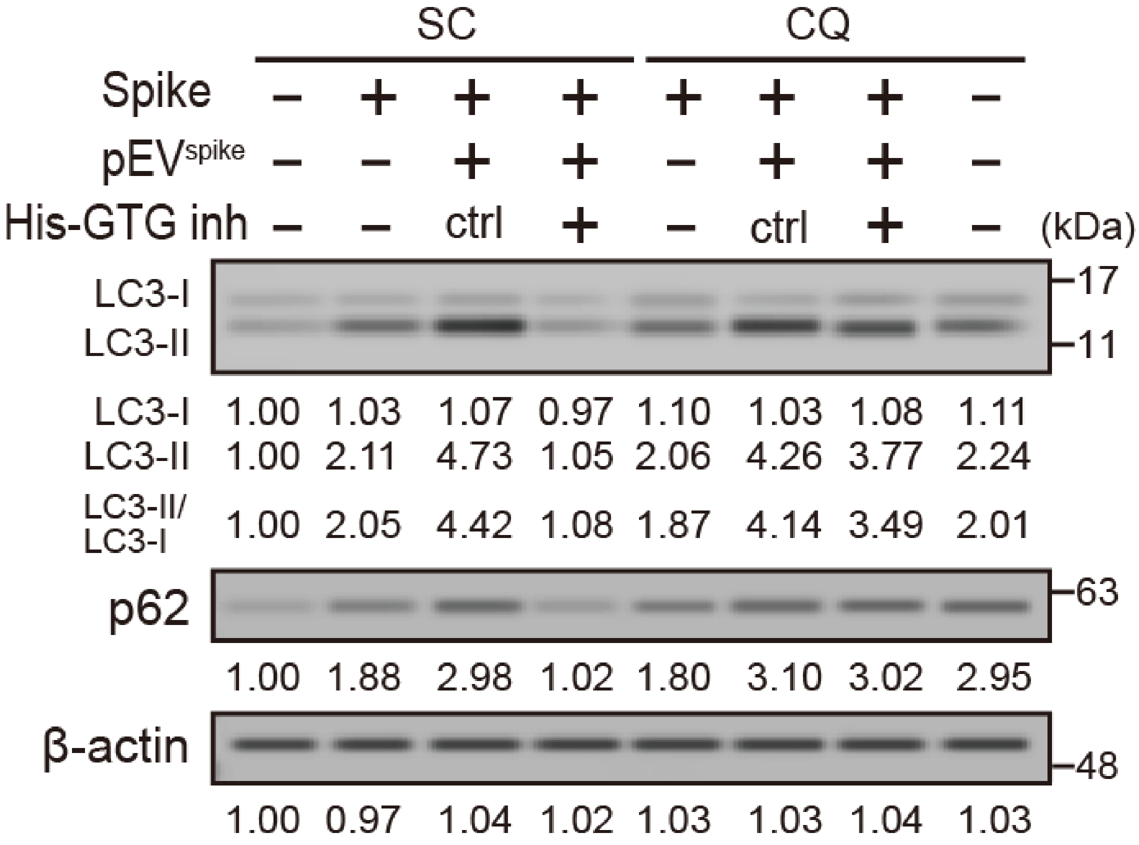

SC
CQ
Spike – + + + + + + –
pEVspike – – + + – + + –
His-GTG inh – – ctrl + – ctrl + –
(kDa)
LC3-I
LC3-II
17
11
LC3-I 1.00 1.03 1.07 0.97 1.10 1.03 1.08 1.11
LC3-II 1.00 2.11 4.73 1.05 2.06 4.26 3.77 2.24
LC3-II/LC3-I 1.00 2.05 4.42 1.08 1.87 4.14 3.49 2.01
p62
63
1.00 1.88 2.98 1.02 1.80 3.10 3.02 2.95
β-actin
48
1.00 0.97 1.04 1.02 1.03 1.03 1.04 1.03

RAB7A (23 kDa)/RAB5 (25 kDa)/VAMP8 (15 kDa)/LAMP1 (100-120 kDa)

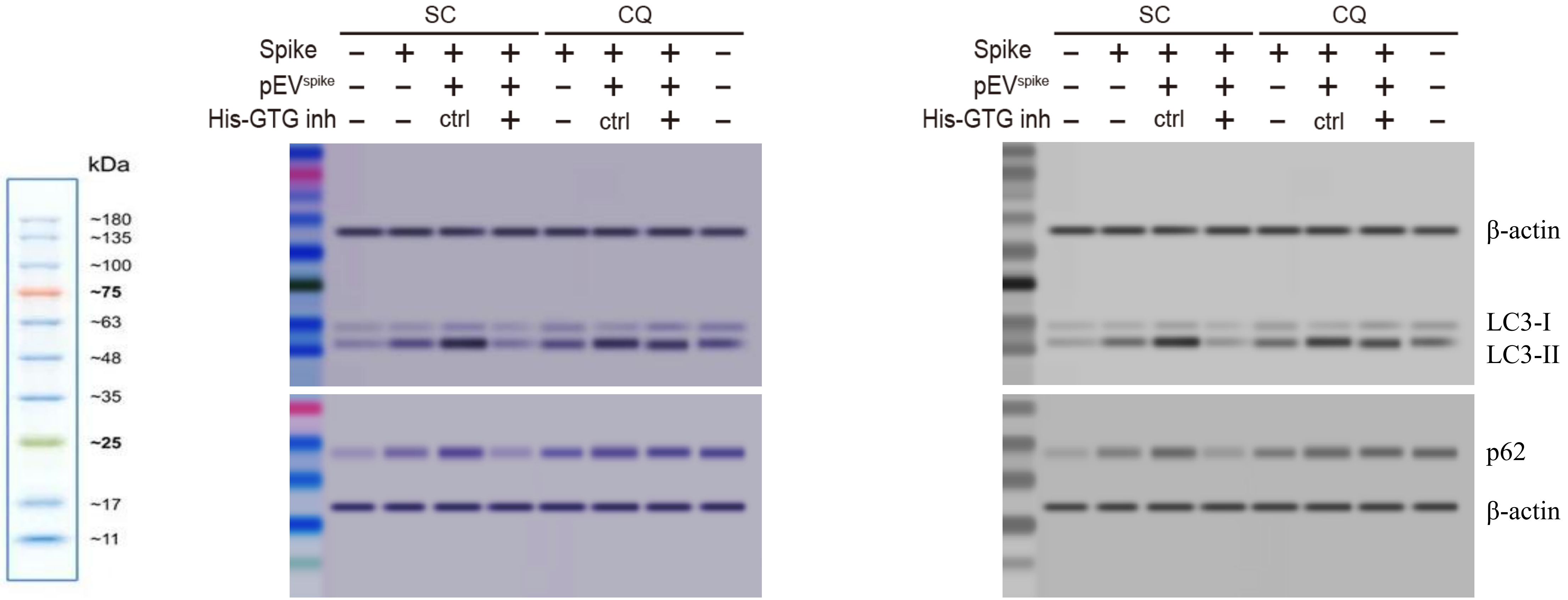

Fig. S10D

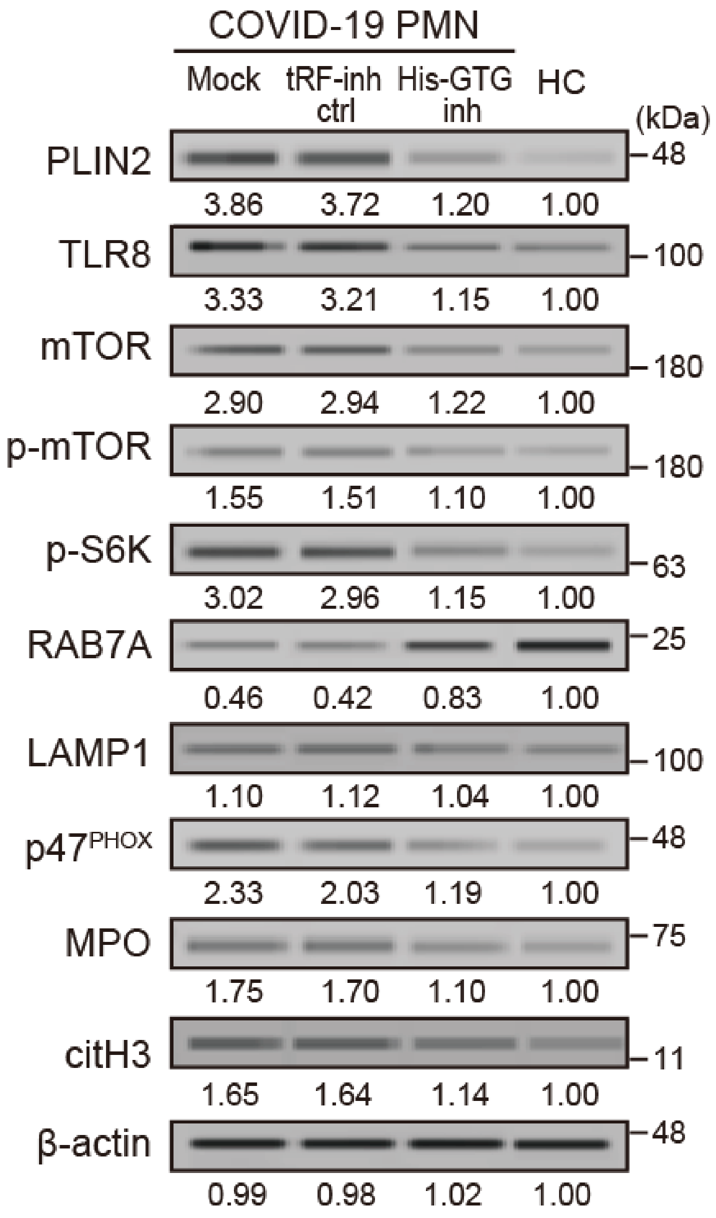
COVID-19 PMN
Mock
tRF-inh ctrl
His-GTG inh
HC
(kDa)
PLIN2
48
3.86 3.72 1.20 1.00
TLR8
100
3.33 3.21 1.15 1.00
mTOR
180
2.90 2.94 1.22 1.00
p-mTOR
180
1.55 1.51 1.10 1.00
p-S6K
63
3.02 2.96 1.15 1.00
RAB7A
25
0.46 0.42 0.83 1.00
LAMP1
100
1.10 1.12 1.04 1.00
p47PHOX
48
2.33 2.03 1.19 1.00
MPO
75
1.75 1.70 1.10 1.00
citH3
11
1.65 1.64 1.14 1.00
β-actin
48
0.99 0.98 1.02 1.00

PLIN2(48 kDa)/TLR8 (110 kDa)/mTOR (289 kDa)/p-mTOR (289 kDa)/p-S6K (70 kDa)

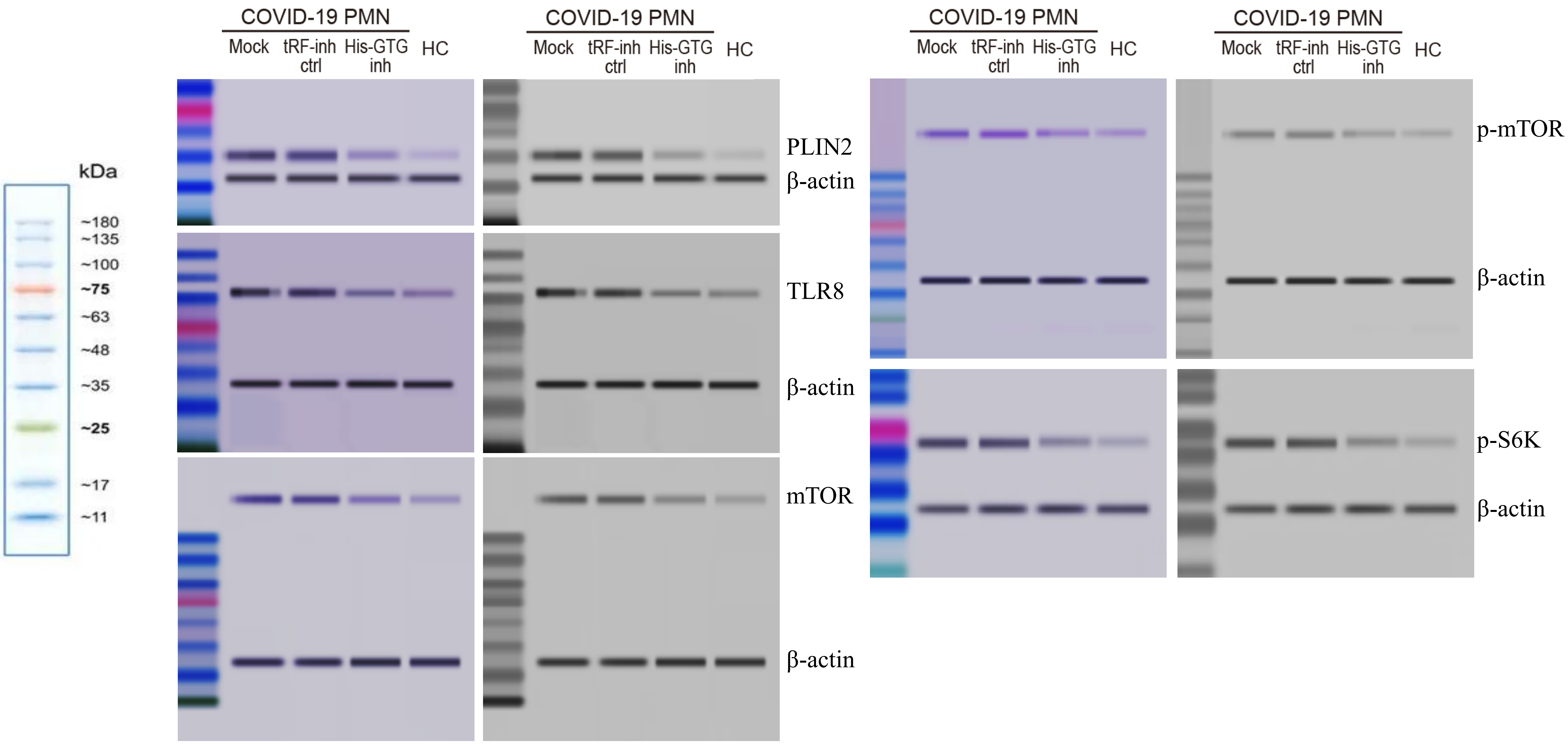

RAB7A (23 kDa)/LAMP1 (100~120 kDa)/p47^{PHOX} (47 kDa)/MPO (62kDa)/citH3 (14 kDa)

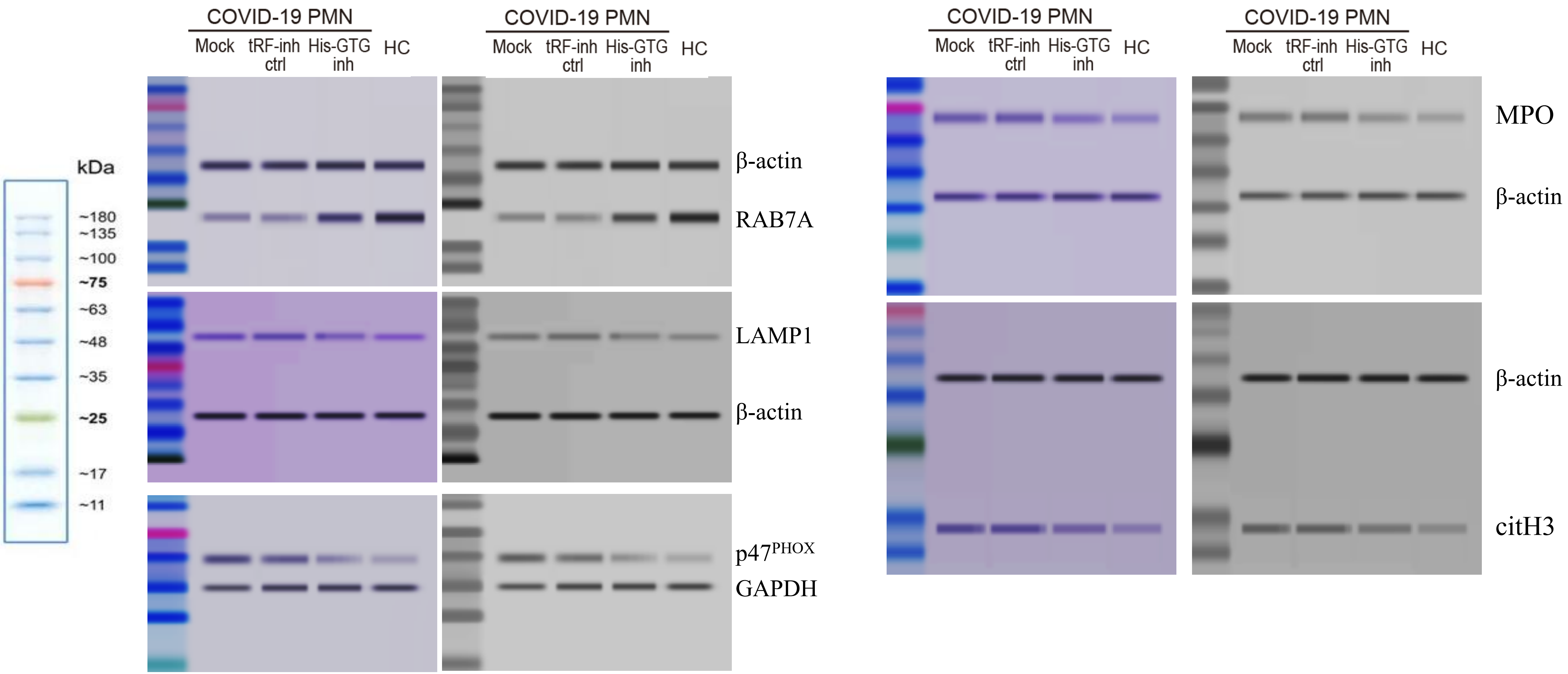

Supplement: Supplementary file 3 — Supporting File 3: advs73502‐sup‐0003‐Additional file 3_Unedited blot and gel images.pdf. [file ADVS-13-e08695-s003.pdf]
